# Design and evaluation of genome-wide libraries for RNA interference screens

**DOI:** 10.1186/gb-2010-11-6-r61

**Published:** 2010-06-15

**Authors:** Thomas Horn, Thomas Sandmann, Michael Boutros

**Affiliations:** 1 German Cancer Research Center (DKFZ), Div. of Signaling and Functional Genomics and University of Heidelberg, Department of Cell and Molecular Biology, Faculty of Medicine Mannheim, Im Neuenheimer Feld 580, D-69120 Heidelberg, Germany; 2 University of Heidelberg, Hartmut Hoffman-Berling International Graduate School for Molecular and Cellular Biology, D-69120 Heidelberg, Germany; 3 University of Heidelberg, CellNetworks Cluster of Excellence, D-69120 Heidelberg, Germany

## Abstract

RNA interference (RNAi) screens have enabled the systematic analysis of many biological processes in cultured cells and whole organisms. The success of such screens and the interpretation of the data depend on the stringent design of RNAi libraries. We describe and validate NEXT-RNAi, a software for the automated design and evaluation of RNAi sequences on a genome-wide scale. NEXT-RNAi is implemented as open-source software and is accessible at http://www.nextrnai.org/.

## Rationale

RNA interference (RNAi) screens have become an important tool for the identification and characterization of gene function on a large-scale and complement classic mutagenesis screens by providing a means to target almost every transcript in a sequenced and annotated genome. RNAi is a post-transcriptional gene silencing mechanism conserved from plants to humans and relies on the delivery of exogenous short double-stranded RNAs (dsRNAs) that trigger the degradation of homologous mRNAs in cells [1,2]. As an experimental tool, RNAi is now widely used to silence the expression of genes in a broad spectrum of organisms [3].

The availability of genome-wide RNAi libraries for cell-based assays and whole organisms has opened new avenues to query genomes for a broad spectrum of loss-of-function phenotypes [4,5]. The number of sequenced genomes is steadily rising, enabling reverse genetic approaches using RNAi in many novel model systems, including, for example, the medically relevant vector *Anopheles gambiae *and species used to study evolutionary aspects of development, such as *Tribolium castaneum*, *Acyrthosiphon pisum *and *Schmidtea mediterranea*. RNAi libraries will facilitate the functional characterization of genes in these species, either through studying smaller subsets of candidates or on a genomic scale.

The design of RNAi reagents is key to obtaining reliable phenotypic data in large-scale RNAi experiments. Several recent studies demonstrated that the degradation of non-intended transcripts (so-called 'off-target effects') and knock-down efficiency depend on the sequence of the RNAi reagent and have to be carefully monitored [6-13]. Based on experimental studies, rules for the design of RNAi reagents have been devised to improve knock-down efficiency and simultaneously minimize unspecific effects.

In invertebrates such as *Caenorhabditis elegans *and *Drosophila*, RNAi can be triggered by long dsRNAs that are intracellularly broken down into short interfering RNAs (siRNAs) [1,14,15]. The design of a long dsRNA therefore needs to take into account both the properties of the target sequence, for example, its sequence complexity, as well as the properties of all siRNAs contained within the long dsRNA, such as their predicted target specificity and efficiency. Because long dsRNAs are often generated by *in vitro *transcription, the design of suitable primer pairs to amplify *in vitro *transcription templates through PCR from genomic DNA or cDNAs must be implemented.

**Figure 1:**
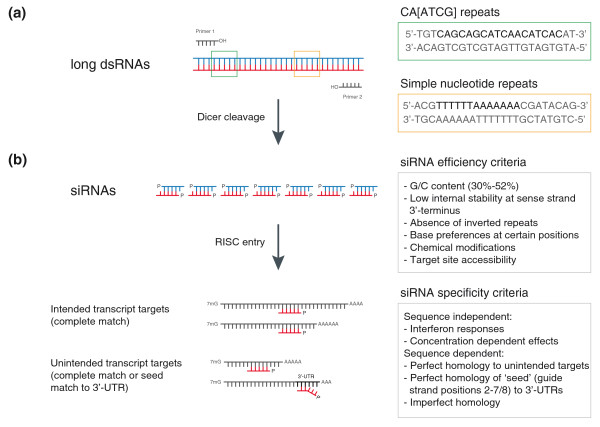
**Quality control parameters for RNAi reagents at different stages of the design pipeline. (a) **Long dsRNAs that have regions of low complexity, for example, CA[ATCG] repeats or simple nucleotide repeats, can exert unspecific and cytotoxic effects. The quality of the primer designs used to synthesize amplicons from DNA sources is crucial, in particular when the synthesis is performed in 96- or 384-well formats where primers should have similar melting temperatures. **(b) **Dicer-mediated cleavage of long dsRNAs leads to the generation of siRNAs of lengths between 19 and 23 nucleotides [29]. The quality of siRNAs depends on their ability to efficiently enter the RNA-induced silencing complex (RISC) and to access the target mRNA. This is influenced by thermodynamic properties, base preferences and chemical modifications. The specificity of siRNAs is influenced by sequence-independent and sequence-dependent features. siRNAs can trigger interferon responses or show concentration-dependent cytotoxic effects, independent of their sequence. Silencing of unintended target transcripts can occur through perfect and imperfect sequence homologies to the siRNA and through 'seed matches' to the transcript 3' UTRs. See text for details.

In contrast, RNAi-mediated silencing in mammalian cells is achieved through siRNAs of 21 to 23 nucleotides [16] to circumvent the activation of an interferon response [17]. Such short dsRNAs can be generated by different methods. For mammalian cells, vectors transcribing short-hairpin RNAs [18-20] or synthetic siRNAs [16] are commonly used. Several recent studies have highlighted favorable sequence characteristics and suitable chemical modifications for these reagents [21-23]. The design of long *in vitro *endoribonuclease-prepared siRNA reagents (esiRNAs) [24] resembles that of long dsRNAs for model organisms. We list several factors that are important for the design of RNAi reagents in Figure 1.

For large-scale functional screens, the design of RNAi reagents is particularly important because the specificity and efficiency of individual RNAi reagents can rarely be validated on a genome-wide scale. The systematic application of design criteria, often on poorly defined gene models, has a direct impact on the expected false positive and false negative rates of phenotypic screens. Previous computational tools are available to design short and long dsRNAs for individual genes [12,25,26]. However, the systematic and reproducible design of RNAi reagents for large sets of genes or even whole genomes using an expanded set of parameters, such as target analysis for all splice isoforms, overlap analysis with SNPs and calculation of seed match frequency, has remained an unresolved issue.

Here we present NEXT-RNAi, a software tool for the design and evaluation of RNAi libraries that can be used for projects with targets ranging from a limited gene set to a whole-genome scale. NEXT-RNAi can process annotations from various sources and thereby provides a powerful RNAi design pipeline for virtually any genome that is available in public databases. NEXT-RNAi can also be used to design independent RNAi reagents to complement existing libraries. To demonstrate its flexibility, we have designed multiple genome-wide RNAi libraries for different organisms, including *Drosophila*, *Anopheles*, *Tribolium *and humans. NEXT-RNAi also offers the opportunity to automatically evaluate and re-annotate existing RNAi libraries by generating user-friendly reports to reflect the regular update of genome annotations.

To validate knock-down efficiency of NEXT-RNAi's reagent designs, we generated two independent sets of long dsRNAs targeting protein and lipid phosphatases expressed in *Drosophila *D.Mel-2 cells and verified transcript knock-down by quantitative real-time RT-PCR.

## Results

### Design of RNAi libraries for genome-scale experiments

RNAi screens rely on the design of large-scale libraries comprehensively covering annotated transcriptomes. The design of RNAi libraries requires the identification of suitable target regions that minimize the potential for off-target effects, increase the silencing capacity and allow an efficient synthesis of the reagents. Often, multiple independent designs that meet these requirements are used to confirm RNAi-induced phenotypes.

**Figure 2:**
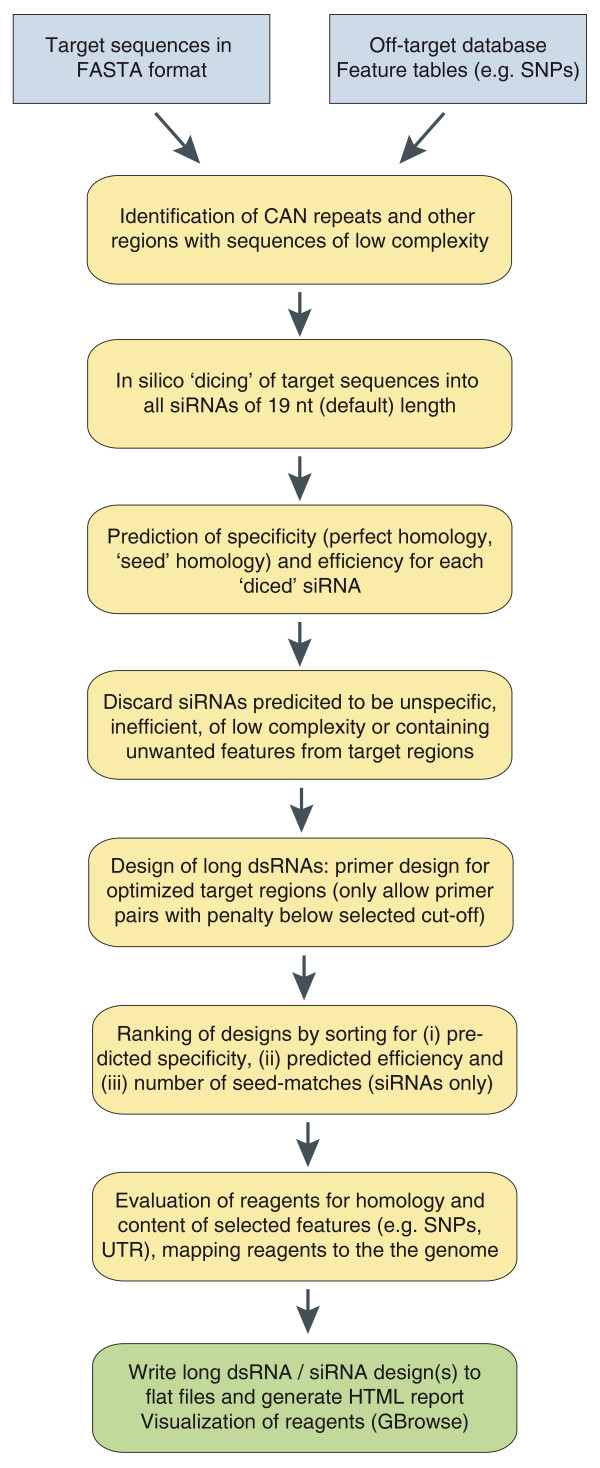
**Overview of the NEXT-RNAi workflow**. NEXT-RNAi requires a defined set of input files in FASTA or tab-delimited formats. First, the program filters the input target sequences for six (default) or more contiguous CAN repeats and for other regions of low complexity (for example, simple nucleotide repeats) using mdust. Sequences are then 'diced' to generate all possible siRNA sequences with a default length of 19 nucleotides (nt) and an offset of 1 nucleotide. Subsequently, each siRNA is mapped to a user-defined off-target database (for example, the whole transcriptome) with Bowtie [37] to determine its specificity. The specificity is set to one if the siRNA targets a single gene or to zero otherwise. In the next step, the predicted efficiency of each 19-nucleotide siRNA is computed. Two methods can be selected, the 'rational' method according to Reynolds *et al*. [9] and the 'weighted' method according to Shah *et al*. [12], assigning each siRNA an efficiency score between 0 and 100. Optionally, the seed complement frequency for each siRNA can be computed for any FASTA file provided (for example, a file containing 3' UTR sequences). siRNAs that did not pass the low-complexity filters, show perfect homology to multiple target genes or do not meet the user-defined cutoffs for efficiency or seed complement frequency are excluded from the queried target sequences. Remaining sequences are used as templates for primer design (with Primer3 [36]) for long dsRNAs or are directly subjected to the final ranking for the design of siRNAs. Designs are ranked by (i) their predicted specificity and (ii) their predicted efficiency and, in the case of siRNA designs, (iii) their calculated seed complement frequency. Sequences can also be evaluated for additional features, such as homology to unintended transcripts, or SNP and UTR contents. Final designs can be visualized using GBrowse [40]. All results are presented in a comprehensive HTML report and are also exported to text files.

**Figure 3:**
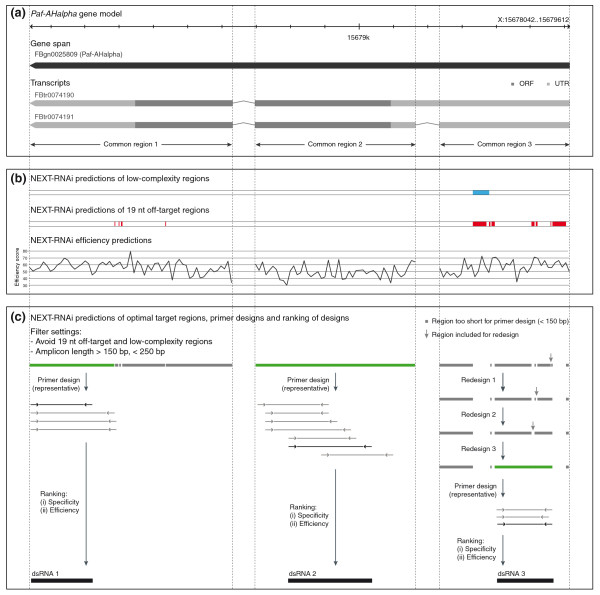
**Example of design and filter methods applied by NEXT-RNAi. (a) **Visualization of the *Paf-AHalpha *gene model and transcripts. Regions labeled as 'common region' serve as input for NEXT-RNAi (ORF = open reading frame, UTR = untranslated region). **(b) **Quality measures computed by NEXT-RNAi for the common regions. Blue and red regions label predicted low-complexity regions (including CAN repeats) and 19-nucleotide off-target regions, respectively. The lower panel shows the predicted siRNA efficiency according to Shah *et al*. [12] (averaged for ten siRNAs). **(c) **NEXT-RNAi predictions of optimal target sites (green) after discarding 19-nucleotide off-target and low complexity regions and regions <150 nucleotides or >250 nucleotides. If available, these regions are directly used as templates for primer designs (left and middle panels). Otherwise, a redesign method is used that connects closest 'optimal' neighbors until a region suitable for primer designs is identified (right panel). Potential dsRNAs are finally ranked by their specificity and efficiency.

Figure 2 illustrates the workflow of NEXT-RNAi for the automated design and evaluation of RNAi reagents (see also Additional file 1), and Figure 3 exemplifies the steps typically performed for the design of a long dsRNA. The input target sequences (Figure 3a) are first analyzed for regions of low complexity that have been shown to exert promiscuous off-target effects [27]. NEXT-RNAi identifies tandem trinucleotide repeats of the type CA[ACGT] (CAN) and can also use the mdust [28] filter program (with default parameters) to find, for example, simple nucleotide repeats or poly-triplet sequences other than CAN (Figure 3b). The function of the intracellular Dicer protein [29] is then simulated by computationally 'dicing' the input target sequences into all possible siRNAs with a (default) length of 19 nucleotides. siRNAs may cause unspecific gene silencing via short stretches of homology with unintended mRNAs [27,30,31] or by a route similar to miRNA-mediated silencing through sequence similarity in positions 2 to 7 or 2 to 8 of the siRNA guide strand to the 3' UTR of unintended transcripts [32,33]. NEXT-RNAi assesses the specificity of siRNAs by mapping them to the transcriptome. An siRNA is considered 'specific' if only isoforms of the same gene are targeted (with perfect homology; Figure 3b). The number of siRNA seed matches (seed complement frequency) is determined by mapping all the unique seeds to a user-defined database containing, for example, 3' UTR sequences. Several criteria can be taken into account to determine the predicted efficiency of an siRNA, including asymmetric thermodynamic properties [8,10], G/C content, structural properties [34] and base preferences at several positions [6,9,11]. NEXT-RNAi implements two scoring methods to assess the predicted siRNA efficiency, here referred to as the 'rational' [9] and 'weighted' [12] methods. Scores range between 0 and 100 (Figure 3b). A previous analysis by Reynolds *et al*. [9] reported that siRNAs with efficiency scores ≥66.7 (on our normalized scale) were efficient silencers in human cells; and Shah *et al*. [12] found that designs with scores ≥63 were efficient. Analysis of 2,431 knock-down validated siRNAs (from Huesken *et al*. [35]) for their predicted efficiency (Additional file 2) shows a good correlation between the normalized inhibitory activity of the siRNAs and the predicted efficiency score (correlation of 0.52 and 0.51 for the 'rational' and 'weighted' methods respectively; *P*-value <2.2e-16).

All quality parameters measured prior to this step, including the prediction of specificity, efficiency and low complexity, are applied as filters on the input sequences to identify optimal RNAi target sites. The set of filters can be further expanded by also including cut-offs on the seed complement frequency and sequence filters on conserved miRNAs seeds (for each siRNA). For the design of long dsRNAs, Primer3 [36] is then used with user-defined settings to design primer pairs required for the PCR during dsRNA synthesis (Figure 3c). In case the optimal target sites identified are too short for designing primers (colored grey in Figure 3c), NEXT-RNAi implements a redesign routine that can be enabled by the user. This routine identifies those optimal target sites that are closest to each other and combines them by including the 'suboptimal' region in between. This step is carried out iteratively until the region is long enough for designing primers (for example, see right panel in Figure 3c).

Long dsRNA or siRNA designs are finally ranked by predicted specificity and predicted efficiency. For the ranking of siRNAs, designs with low seed complement frequency are prioritized. Since long dsRNAs contain many different siRNAs, two efficiency scores are reported: the average efficiency score of all contained siRNAs and the absolute number of efficient siRNAs (efficiency above a user-defined cutoff). The user-defined number of top-ranked designs for each target can be evaluated further by mapping them to the genome (with Bowtie [37] or Blat [38]), by determining the overall homology to other transcripts (with Blast [39]) or by calculating the overlap with other sequence features such as SNPs or UTRs.

NEXT-RNAi outputs design information in a tab-delimited text file and generates a comprehensive HTML report including a graphical display of designs in GBrowse [40] (Additional files 3 and 4). Further, details are available in FASTA, GFF (generic feature file) and AFF (annotation file format) formats for additional sequence analyses and straightforward reagent visualization in any genome browser.

### Application of NEXT-RNAi for RNAi reagent design

We next set out to apply the software to design novel genome-wide RNAi libraries for different organisms, including *Drosophila melanogaster*, *T. castaneum*, *A. gambiae *and *Homo sapiens*.

#### *Drosophila*

Several RNAi libraries for cell-based [41] and *in vivo *RNAi screens (Vienna *Drosophila *RNAi Center (VDRC) [4], Fly stocks of National Institute of Genetics (NIG-Fly) and Transgenic RNAi Project (TRiP) libraries) have been constructed covering almost all genes annotated in the *Drosophila *genome. We used NEXT-RNAi to design multiple independent long dsRNAs targeting all *Drosophila *genes based on the latest genome release (FlyBase [42] release 5.24) in one run. Each design targeted all splice variants of a given target gene. To this end, we computed regions common to all annotated isoforms of the 14,898 coding or non-coding *Drosophila *genes. To further increase the number of potential target sites, we split common regions longer than 700 nucleotides into two sequences of equal length. This resulted in 74,907 common regions overall or an average of five regions per gene to be used as input for NEXT-RNAi. The *Drosophila *transcriptome was used as a database to evaluate the siRNA specificities ('off-target' database). NEXT-RNAi design options were adjusted to exclude low-complexity regions, CAN repeats, 19-nucleotide siRNA matches to unintended transcripts and siRNAs containing miRNA seeds (as predicted by miRBase [43]). The length-window for long dsRNA designs was set to 80 to 250 nucleotides. We included an iterative redesign, as described above, for sequences initially failing to meet these criteria. The best design for each input sequence was further evaluated for homologies (Blast E-value <1e-10) to unintended transcripts and for overlaps with UTRs.

The NEXT-RNAi output is exemplified in Additional files 3 and 4. Summarized results for the designs are presented in Additional file 5. The full report is available on our companion website [44]. In total, 70,149 designs were calculated, covering 99.4% of all annotated genes with at least one dsRNA and 88.7% with multiple independent designs. Eighty-three gene models could not be targeted because of gene-spanning low complexity regions. Each gene model was, on average, targeted by 4.7 independent designs, 90.7% of which lack any perfect homology to any location other than the intended target transcripts of more than 18 nucleotides. In some cases, dsRNAs including 19-nucleotide matches could not be avoided, for example, for paralogous gene families with high sequence similarities or long overlaps (for example, actin or histone families).

#### *Tribolium*

A similar approach was used to generate independent designs for all predicted exons included in the 'official gene set' (available from BeetleBase [45]) of the recently sequenced genome of the red flour beetle, *T. castaneum *[46]. *Tribolium *has become an important model organism for developmental and evolutionary studies, and efficient RNAi through injection of long dsRNAs has been demonstrated [47].The newly designed RNAi reagents covered 99.4% of all predicted gene models (83.2% with multiple independent designs), of which 92.9% lacked any predicted 19-nucleotide off-targets (Additional file 5).

#### *Anopheles*

The mosquito *A. gambiae *is widely studied to analyze the mechanism of innate immunity as a vector for *Plasmodium falciparum*. RNAi by long dsRNAs has been demonstrated *in vitro *and *in vivo *and leads to efficient depletion of mRNAs [48]. Based on VectorBase [49] annotations, we designed RNAi reagents covering 95% of all genes (90.1% of all genes were covered by independent designs). Of all the designs, 89.2% had no unintended 19-nucleotide match in the *Anopheles *transcriptome (Additional file 5).

#### Human genome

RNAi experiments in mammalian systems require the application of either *in vitro*-diced long dsRNAs (esiRNAs) or synthetic siRNAs. Here we designed reagents for both approaches to target all human genes annotated by the National Center for Biotechnology Information (NCBI) RefSeq database [50] (Additional file 5). Regions common to all RefSeq transcripts of the same gene were computed for all human genes and used as target sites for multiple independent esiRNA and siRNA designs per gene. Although both libraries covered almost the entire genome (esiRNAs, 97.8%; siRNAs, 99.9%), siRNA designs allowed a higher coverage and targeted more genes without predicted 19-nucleotide homologies to unintended transcripts (83.4% (siRNA) compared to 73.8% (esiRNAs) of the genome). The mean of predicted efficiency scores for siRNA designs was 84.76 (the 'weighted' method was used with a cutoff of 63), about 39% of the designs have low seed complement frequencies (less than 1,000 seed matches; RefSeq annotated 3' UTRs were used for seed match computation) and about 12% of the siRNAs contain annotated SNPs (from dbSNP [51]), which can interfere with siRNA function.

The complete description and NEXT-RNAi reports of the libraries for different organisms are available at [44]. Similarly, NEXT-RNAi could be applied to other recently sequenced genomes, including *Schmidtea mediterannea *and *Acyrthosiphon pisum*, for which RNAi has become the method of choice for functional experiments.

### NEXT-RNAi for the evaluation of existing RNAi libraries

A challenge for the interpretation of screening experiments is the correct annotation of available RNAi reagents; this includes the assessment of quality control parameters, their mapping to the genome and updating their target information for new genome annotation releases.

NEXT-RNAi enables the re-calculation of specificity, efficiency and other features of libraries of long dsRNAs and siRNAs. As examples, we performed a re-annotation of eight large-scale RNAi libraries designed for the *Drosophila *genome (Ambion, Heidelberg 2 (HD2), Heidelberg Fly Array/*Drosophila *RNAi Screening Center (DRSC) v1.0 [52], DRSC v2.0 [41], OpenBiosystems v1/v2, Medical Research Council (MRC), NIG-Fly and VDRC [4]) using the FlyBase annotations of release 5.24 (Additional file 6). With the exception of the HD2 and DRSC v2.0 libraries, all libraries covered less than 90% of the genome. This might result in part from the fact that they were designed for previous genome releases (release 3 or earlier). A comparison between the libraries showed how the design strategies evolved over time. While the designs of the HD2 and DRSC v2.0 libraries avoided both 19-nucleotide off-target effects (26.6% and 31.1% of all dsRNAs in HD2 and DRSC v2.0, respectively) and CAN repeats (0.5% and 1.8% of all dsRNA in HD2 and DRSC v2.0, respectively), older libraries, including DRSC v1.0 and MRC, contain a significantly higher percentage of dsRNAs with predicted 19-nucleotide off-targets (37.1% and 51.5%, respectively) and CAN repeats (5.3% and 5.4%, respectively). NEXT-RNAi also allows for the assessment of further parameters of the reagents. In this analysis, we computed the number of siRNAs with known miRNA seeds (from miRBase [43]) contained within each long dsRNA. With an average of 1.9, the Ambion library contains the fewest miRNA seeds per dsRNA, potentially because Ambion dsRNAs are rather short (255 nucleotides). Analyzing long dsRNAs for overlaps with UTRs reveals that designs in the DRSC v1.0, MRC, NIG-Fly and VDRC libraries were aimed at targeting open reading frames only (in all of these libraries, less than 8% of the reagent targets predicted UTRs).

**Figure 4:**
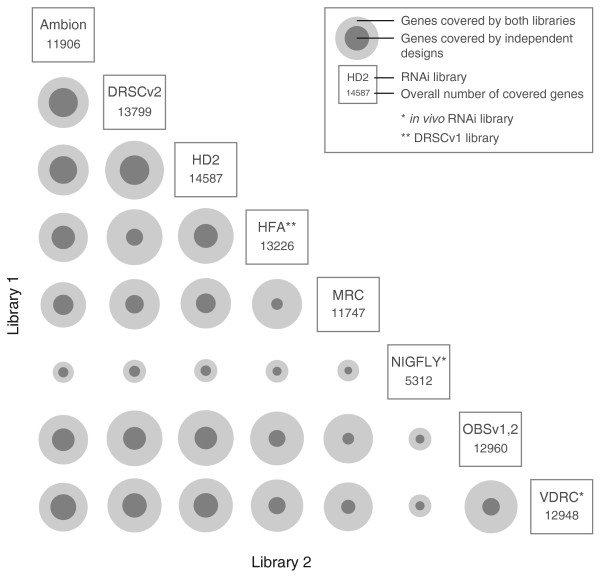
**Pairwise comparison of *Drosophila *RNAi libraries**. Eight *Drosophila *RNAi libraries are compared to identify the number of genes that are targeted by multiple libraries (outer ring, light grey) and the number of genes that are targeted by independent designs (inner ring, dark grey). The reference for the ring sizes is the combination of libraries commonly targeting the most genes (HD2/DRSCv2: 13,197 genes).

An important experimental step during the confirmation of candidate genes from RNAi screens is the validation of phenotypes with independent designs [53]. We used NEXT-RNAi results to identify the number of genes that could be targeted with independent designs through pairwise combinations of all *Drosophila *RNAi libraries (Figure 4; Additional file 7; complete reports are available for download). Pairwise combinations of the Ambion, DRSC v2.0 and HD2 libraries provide the highest number of independent reagents (for example, 6,623 genes covered by HD2 are covered by at least one independent design in DRSC v2.0). Some libraries overlap to a large extent and would be less advisable to use for confirmation screening. For example, combining the DRSC v1.0 and MRC libraries covers only 2,593 genes by independent designs. The analysis done provides also a helpful resource to identify *in vivo *RNAi lines of VDRC and NIG-Fly libraries that can be used for confirmation experiments with a second, non-overlapping dsRNA.

We also re-annotated human siRNA libraries from Ambion (Silencer Select Library) and Qiagen (human druggable v3.0 and human whole genome supplement v1.0), containing 64,781 and 70,308 siRNAs, respectively (Additional file 6). Of all siRNAs in the Ambion and Qiagen libraries, 3.4% and 10.1%, respectively, lacked any annotated target gene in NCBI RefSeq release 40; 84.2% and 92.4% show perfect homology to a single target gene; and 5.7% and 4.1% perfectly match multiple targets. The libraries cover 75% and 65.3% of all currently annotated NCBI and Entrez genes, respectively. About 9% of siRNAs in both libraries contain annotated SNPs (from dbSNP). More than one-third of the siRNAs in the Qiagen library overlap with annotated UTRs in their target transcripts (by at least one base), but only about one-tenth of the siRNAs in the Ambion library do so. Libraries also differ in the mean of predicted siRNA efficiency scores (using the 'weighted' method), with 74.65 for the Ambion and 57.58 for the Qiagen library. Of the Ambion and Qiagen siRNAs, 9.8% and 5.3%, respectively, have low seed complement frequencies (less than 1,000 seed matches in RefSeq annotated 3' UTRs).

### Knock-down validation of NEXT-RNAi designs for *Drosophila *phosphatases

To validate the knock-down efficiency of reagents designed by NEXT-RNAi, we designed two independent long dsRNAs (see Additional file 8 and companion website for details on the design) for all *Drosophila *protein- and lipid-phosphatases expressed in D.Mel-2 cells (Gene Expression Omnibus (GEO) accession [GEO:GSE21283]). We found 49 phosphatases expressed at five or more RPKM (reads per kilobase gene per million reads; Additional file 9). The reagents were synthesized using a two-step PCR procedure followed by *in vitro *transcription [14] with a 100% synthesis success rate.

**Figure 5:**
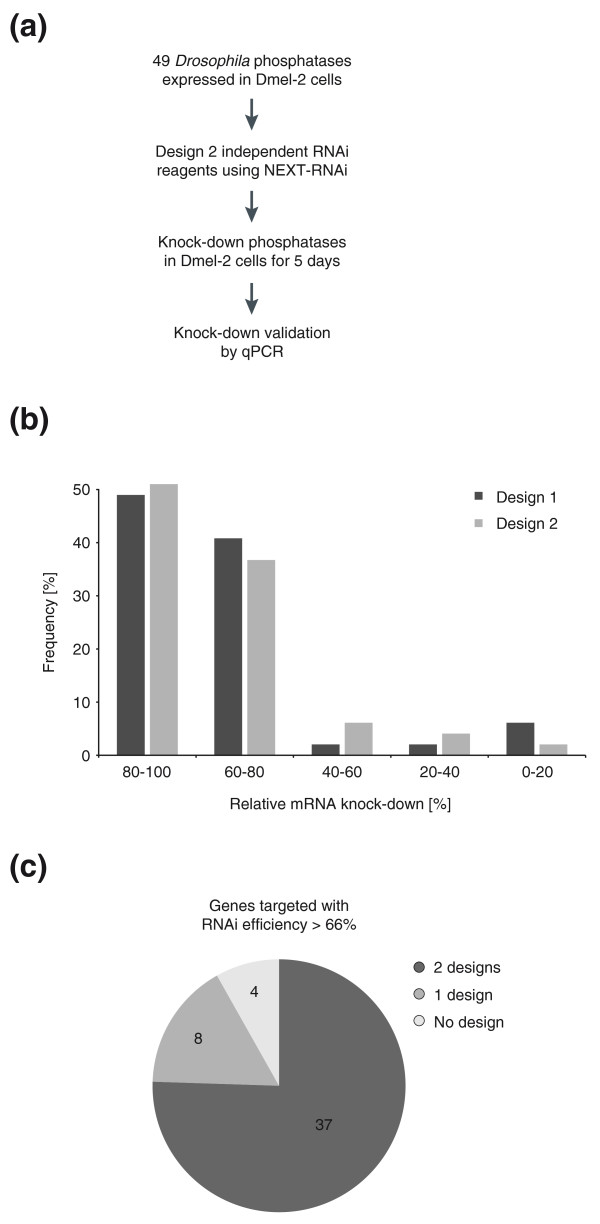
**Knock-down validation of *Drosophila *phosphatases. (a) **Experimental workflow for knock-down validation. **(b) **Frequencies of observed knock-down efficiencies for independent designs. **(c) **The number of genes efficiently silenced (knock-down >66%) by both independent designs, only one or neither design. qPCR, quantitative RT-PCR.

After RNAi knock-down for 5 days (Figure 5a), transcript levels were determined using quantitative RT-PCR. Out of 98 dsRNAs, 87 (88.8%) caused a decrease in mRNA levels of more than 60%; half of the dsRNAs achieved a knock-down exceeding 80% (Figure 5b; Additional file 10). Eleven mRNAs showed little or no knock-down, six of which could not be detected reproducibly in this assay. For 37 of the 49 genes, we found that both independent designs decreased mRNA levels by at least two-thirds. For eight genes, only one design and for four genes, no designs could be validated with this knock-down strategy (Figure 5c).

Overall, our results show that NEXT-RNAi designs efficiently silenced targeted mRNAs. Furthermore, the independent designs led to highly reproducible knock-downs (Pearson correlation coefficient of 0.85), indicating that the observed depletion efficiency depended on the targeted mRNA rather than differences in the NEXT-RNAi designs.

## Discussion

In large-scale RNAi experiments, the design of genome-wide silencing libraries has remained an important problem due to the flux of gene annotation and novel insights into the mechanisms that influence RNAi efficiency and off-target effects. We present an approach for the rapid design of whole-genome RNAi libraries and the re-annotation of already existing reagent collections. The method is flexible, identifies multiple independent reagents per gene model and has been implemented in an organism-independent manner. The design process is fully automated and can use annotations from various sequence- or model-organism databases as input, thereby enabling the design of RNAi reagents for any sequenced (and annotated) organism.

We have designed several independent RNAi libraries for a diverse group of organisms. The automated pipeline yielded designs for more than 95% of all predicted genes in the first round of prediction. All library designs are available as a resource for download from our webpage [44]. We validated the knock-down of 98 long dsRNAs directed against 49 *Drosophila *phosphatases expressed in our tissue culture model and found that approximately 89% of the reagents caused at least 60% mRNA knock-down. The application of a standardized design pipeline for independent designs leads to reproducible knock-downs in our experiments (correlation of 0.85 between the independent designs).

RNAi screens have become a key tool for functional genomic analyses. The interpretation of the increasing number of published data sets obtained through RNAi screens relies heavily on correctly annotated reagents. Phenotypes derived from large-scale screens should be linked to the sequence of the RNAi reagent rather than the gene model because off-target or splice-variant-specific silencing can rarely be excluded. For the correct interpretation of RNAi screens, and also the comparison between different libraries, reagent-to-gene-model linkages must be re-mapped in regular intervals because most genome annotations are still in flux. NEXT-RNAi can be used to rapidly evaluate and re-annotate existing genome-wide libraries. For example, we have applied the algorithm to re-annotate RNAi libraries for *Drosophila *and human cells. Our analysis of eight genome-wide RNAi libraries for *Drosophila *revealed differences in genome coverage and predicted quality (for example, specificity), most likely depending on two factors: the quality of the underlying genome release and the factors known to influence reagent quality at the time of the library design. Further, reagents in these libraries often share target sites, thus preventing an independent confirmation of phenotypes on a genomic scale. The re-annotation of commercially available human libraries revealed that a substantial part of the siRNAs (Ambion library, 15.8%; Qiagen library, 7.5%) either do not target the intended gene or are predicted to silence additional loci, demonstrating that quality control at the level of sequence mapping is crucial for the interpretation of large-scale screens.

Several tools for the design of RNAi reagents exist (including, for example, E-RNAi [25], DEQOR [26], SnapDragon [54], and siR[12], and commercial design tools such as siDESIGN Center (Dharmacon, ThermoScientific), BioPredsi (Qiagen) and siRNA Target Finder (Ambion)). However, these tools can only be used for designing long dsRNAs or siRNAs on a gene-by-gene basis. In contrast to available tools, our method allows for rapid batch design and evaluation of RNAi libraries for complete genomes or for any defined set of genes. In addition, our approach uses multiple parameters to calculate or evaluate designs, including sequence complexity, efficiency and specificity indicators, and allows for further refinement by scoring overlap with SNPs or UTRs. The software pipeline can also be used to obtain multiple independent RNAi designs per gene for independent validation of RNAi phenotypes. Additional strengths of NEXT-RNAi are its speed in designing comprehensive libraries and the generation of HTML reports including a variety of output options.

RNAi screening is being used increasingly in diverse organisms that only recently became amenable to genomic approaches. NEXT-RNAi can be deployed to design RNAi reagents for any sequenced genome to facilitate a better understanding of gene function through improved RNAi tools. This can be of particular utility for emerging model organisms that are suitable for large-scale RNAi studies but lack RNAi libraries. Further, in contrast to various microarray platforms, little attention has been paid to the re-annotation of existing RNAi screening data. We provide a fast and flexible software that accelerates the construction of consistent phenotypic data sets from RNAi screening experiments and helps to functionally annotate genome sequences.

## Materials and methods

### Sequences and databases

NEXT-RNAi requires a defined set of files and parameters as inputs. Sequence input files are provided in FASTA format; feature input files, such as transcript-gene relationships or the locations of SNPs and UTRs, are provided in a tab-delimited format using defined names in the header row. Genome annotations and sequences for *Drosophila *were obtained from FlyBase [42]; *Tribolium *annotations and sequences were downloaded from BeetleBase [45]; *Anopheles *annotations and sequences were downloaded from VectorBase [49]; and all annotations and sequences for the human genome were obtained from the NCBI RefSeq database [50].

### Implementation and availability of the NEXT-RNAi software package

NEXT-RNAi is implemented in Perl. It requires the installation of Bowtie [37] and Primer3 [36]. To utilize all options of NEXT-RNAi, the BLAST [39], BLAT [38], RNAfold [55] and mdust [28] programs are also required. On a Linux server (two Intel Xeon Quad-core 2.00 GHz CPUs, 16 GB RAM) running Ubuntu 9.10 server edition, the design of a genome-wide RNAi library for the *Drosophila *genome with approximately 70,000 constructs took about 4 hours. NEXT-RNAi software, installation packages and instructions for Linux and Mac operation systems and further documentations are accessible via [44]. In addition, a platform-independent virtual machine (running on VirtualBox) with NEXT-RNAi and all dependencies pre-installed is available for download. NEXT-RNAi is used as a command line utility with parameters provided in an options file that allows specification of the design and annotation parameters (Additional file 11). An interactive mode that prompts for all necessary settings has been implemented.

### RNA sequencing of *Drosophila *D.Mel-2 cells

D.Mel-2 cells (Invitrogen, Carlsbad, CA, USA) were grown in Express Five SFM (Invitrogen) supplemented with 20 mM Glutamax I, 100 U/ml penicillin, 100 μg/ml streptomycin. Total RNA was extracted using Trizol (Invitrogen), followed by Rneasy cleanup (Qiagen, Hilden, Germany), including on-column DNAse digest. mRNA was isolated with the MicroPoly(A)Purist kit (Ambion, Austin, TX, USA) and the RNAseq library was prepared according to Illumina's mRNA Sequencing Sample Preparation Guide. Paired-end reads were aligned to the *D. melanogaster *genome using Tophat [56] and RPKM values for each gene calculated with Cufflinks [57] based on the *D. melanogaster *gene annotation release 5.13 obtained from Ensembl. The data have been deposited in NCBI's GEO and is accessible through GEO Series accession number [GEO:GSE21283].

### Validation of RNAi knock-down in *Drosophila *D.Mel-2 cells

Long dsRNAs were synthesized using a two-step PCR procedure followed by *in vitro *transcription as described in [14]. The concentration of each dsRNA was determined by photospectrometry and normalized to 50 ng/μl. We aliquoted 250 ng of each reagent in 384-well plates, and D.Mel-2 cells were added to the plates for an incubation time of 5 days. mRNA knock-down was measured by quantitative real-time PCR of two biological replicates using a SybrGreen assay (quantitative real-time PCR primers were designed using QuantPrime [58]).

### Content of the companion website

The companion website to NEXT-RNAi at [44] contains extensive documentation and enables downloading of the complete software. The website also hosts complete NEXT-RNAi outputs for all pre-designed libraries, library evaluations and other analysis done for this manuscript.

## Abbreviations

bp: base pair; CAN: CA[ACGT] repeats; DRSC: *Drosophila *RNAi Screening Center; dsRNA: double-stranded RNA; esiRNA: endoribonuclease-prepared siRNA; GEO: Gene Expression Omnibus; HD2: Heidelberg 2; miRNA: microRNA; MRC: Medical Research Council; NCBI: National Center for Biotechnology Information; NIG-Fly: Fly stocks of National Institute of Genetics; RNAi: RNA interference; RPKM: reads per kilobase gene per million reads; siRNA: short interfering RNA; SNP: single nucleotide polymorphism; UTR: untranslated region; VDRC: Vienna *Drosophila *RNAi Center.

## Authors' contributions

TH and MB developed the concept. TH wrote the software and performed all calculations presented in the manuscript. TH and TS carried out the experimental validation of RNAi reagents. TH and MB wrote the manuscript.

## Supplementary Material

Additional file 1 **Detailed NEXT-RNAi workflow for the (a) design and (b) evaluation of dsRNAs and siRNAs**. Click here for file

Additional file 2 **NEXT-RNAi predictions of siRNA efficiencies using both the 'rational' and 'weighted' methods for 2,431 siRNAs tested by Huesken *et al*. **[35]. Click here for file

Additional file 3 **NEXT-RNAi summary HTML page for the design of a genome-wide RNAi library for the *Drosophila *genome**. This page provides information about the number of successful designs (here, about 94% of the 74,907 query-sequences could be covered with long dsRNA designs). The 'Links to HTML results' link to detailed reports (Additional file 4) for each design (the full list of links was cut for this figure). 'Links to result files' directly link to NEXT-RNAi output files, such as the tab-delimited result file (the main output file) summarizing all calculations done in one line per design, a FASTA file only containing the final reagent sequences as well as GFF (generic feature file) and AFF (annotation file format) output files for visualization and direct upload of reagents to a genome browser, respectively. Further, links to the user-input text files and to report files (for example, reports about failed designs) are provided. Click here for file

Additional file 4 **Detailed output for a long dsRNA that targets the *Drosophila *gene *csw *(FBgn0000382)**. The box 'dsRNA information' provides information about the primers (for example, sequence, melting temperature, GC content) required for the synthesis. 'Primer pair penalty' is an overall quality score for the primer pair. The lower this score is, the higher is the predicted quality of the primer pair. Further, the full amplicon sequence, its length and location in the genome (in the format chromosome:start..end(orientation)) are presented. The 'Target information' box shows the intended target(s) and transcript(s) as well as other (unintended) targets and transcripts ('NA' means that no target was found). The intended transcripts are those with most siRNA hits (here, all 203 19-nucleotide siRNAs target the 4 isoforms of *csw*). The intended gene is then defined over the intended transcripts. The 'Reagent quality' box shows the overall number of siRNAs (here 19-nucleotide siRNAs) contained within the long dsRNA sequence, the number of siRNAs that are 'On-target' (the intended target) and those that are 'Off-target' or have 'No-target'. Further quality features computed for this run were the number of conserved miRNA seeds ('mirSeed') in this dsRNA, the number of 'Efficient siRNAs' (here equal to the overall number of siRNAs, since the efficiency cutoff was set to 0), the 'Average efficiency score' (mean efficiency score of all siRNAs contained in the long dsRNA), and the number of 'Low complexity regions' and 'CAN' repeats contained in the long dsRNA. Additionally, the overlap to UTRs (this long dsRNA completely overlaps with annotated UTRs) and the sequence homology to all transcripts (here only to the intended target) were analyzed in this run. The 'Genome Browser' box visualizes the long dsRNA in its genomic context. Click here for file

Additional file 5 **Summary statistics of RNAi reagents designed by NEXT-RNAi for different organisms**. NEXT-RNAi was used to design RNAi reagents for all annotated transcripts included in the latest available genome release. CAN = CA[ACGT] repeats; UTR = untranslated region; SNP = single nucleotide polymorphism. Click here for file

Additional file 6 **Summary statistics for *Drosophila *and human RNAi libraries re-annotated by NEXT-RNAi**. CAN = CA[ACGT] repeats; UTR = untranslated region; SNP = single nucleotide polymorphism. Click here for file

Additional file 7 **Raw data for comparison of *Drosophila *RNAi libraries in Figure **4, **including number of genes targeted by each library, number of genes targeted by both the compared libraries and number of genes targeted with independent designs (with no sequence-overlap at all)**. Click here for file

Additional file 8 **Primer sequences and target gene information for the independent long dsRNAs designed against 49 *Drosophila *phosphatases for the knock-down validation study presented in Figure **5. Click here for file

Additional file 9 **RPKM (reads per kilobase gene per million reads) values for 49 *Drosophila *phosphatases from RNA-sequencing of D.Mel-2 cells and knock-downs measured after RNAi with two independent designs by quantitative RT-PCR (Figure **5; **Additional file **10). Click here for file

Additional file 10 **Results for knock-down validation of two independent RNAi reagents against 49 *Drosophila *phosphatases**. Target-genes were sorted for the measured mRNA knock-down of design one. Click here for file

Additional file 11 **Descriptions and default values of design parameters used for NEXT-RNAi version 1.31**. Click here for file
