# Supplementary figures and images for: Design and evaluation of genome-wide libraries for RNA interference screens
Source: Genome Biol. 2010 Jun 15;11(6):R61. doi: 10.1186/gb-2010-11-6-r61 (PMC2911109; doi:10.1186/gb-2010-11-6-r61)

a

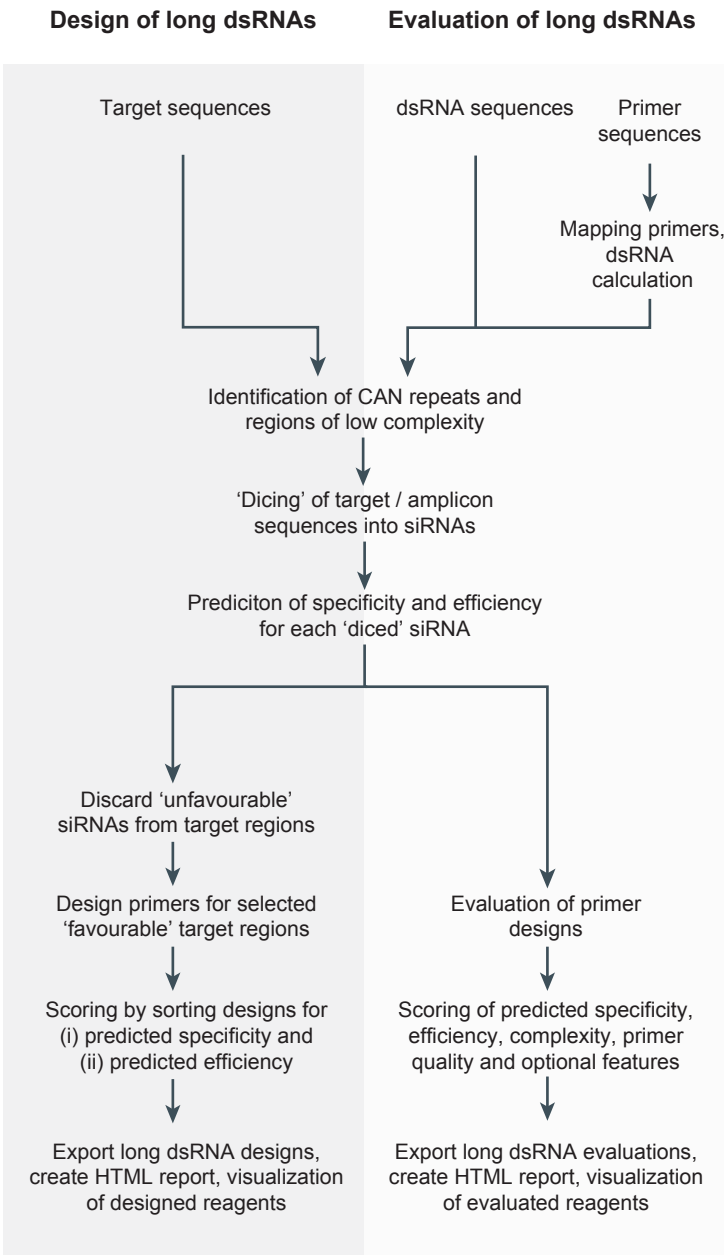

b

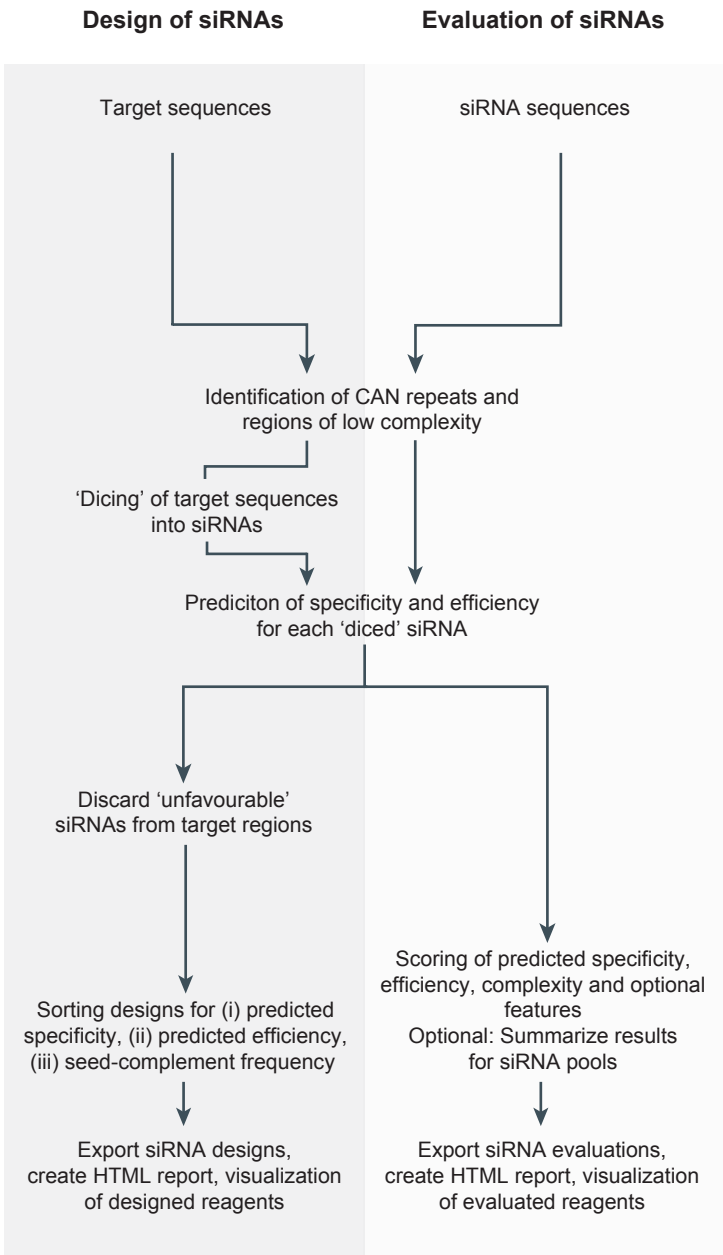

Supplement: Additional file 1 — Detailed NEXT-RNAi workflow for the (a) design and (b) evaluation of dsRNAs and siRNAs. [file gb-2010-11-6-r61-S1.PDF]

Relative mRNA knock-down in Dmel-2 cells after 5 days RNAi

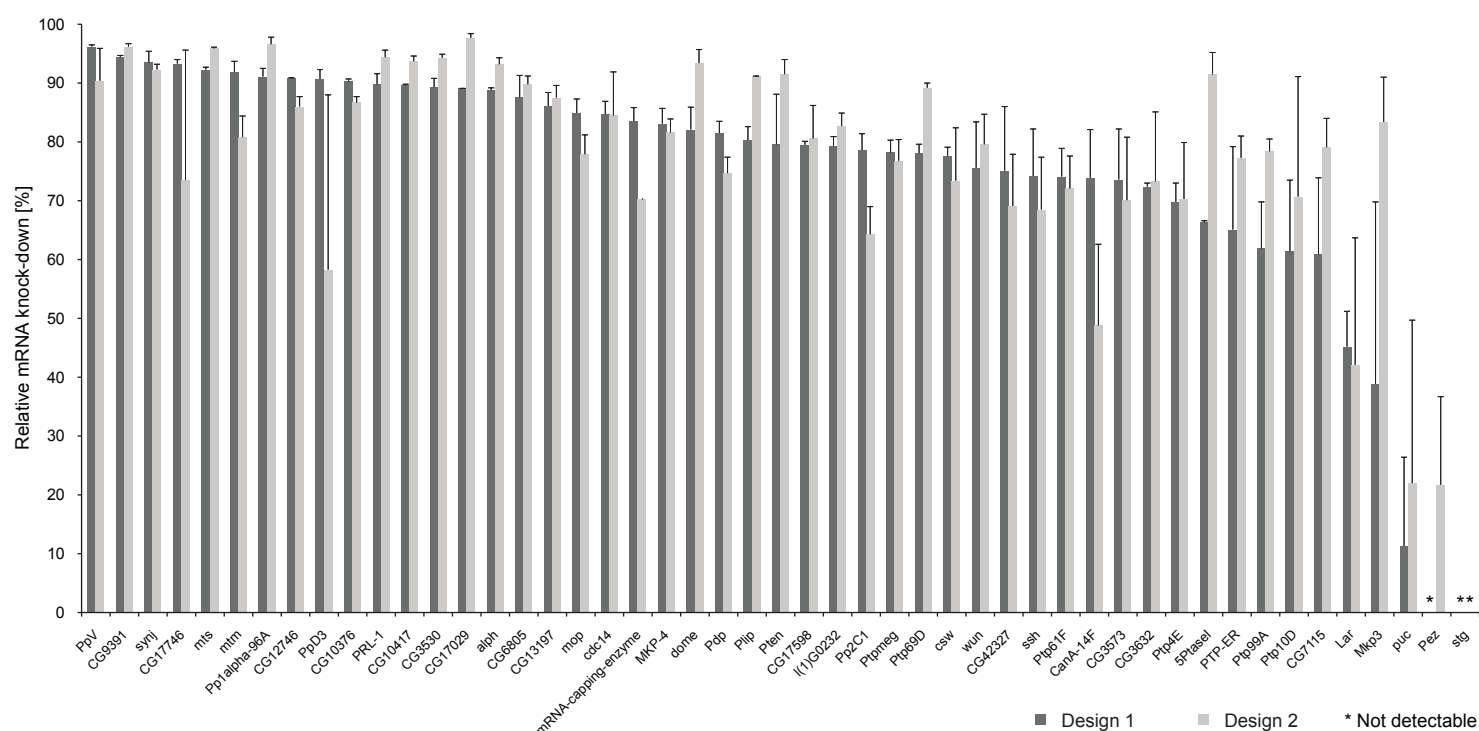

Supplement: Additional file 10 — Results for knock-down validation of two independent RNAi reagents against 49 Drosophila phosphatases. Target-genes were sorted for the measured mRNA knock-down of design one. [file gb-2010-11-6-r61-S10.PDF]
