# Supplementary material for: Design and evaluation of genome-wide libraries for RNA interference screens
Source: Genome Biol. 2010 Jun 15;11(6):R61. doi: 10.1186/gb-2010-11-6-r61 (PMC2911109; doi:10.1186/gb-2010-11-6-r61)
Supplement: Additional file 2 — NEXT-RNAi predictions of siRNA efficiencies using both the 'rational' and 'weighted' methods for 2,431 siRNAs tested by Huesken et al. [35]. [file gb-2010-11-6-r61-S2.PDF]

Additional file 2		NEXT-RNAi efficiency prediction for siRNAs from Huesken *et al.*		
siRNA_ID	siRNA_sense_strand	Normalized_Inhibitory_Activity (Huesken et al.)	NEXT-RNAi efficiency ("weighted" method)	NEXT-RNAi efficiency ("rational" method)
16802	CTAATATGTTAATTGATTT	0.462	51.62	58.33
16803	AATATGTTAATTGATTTAT	0.384	64.57	83.33
16804	GATTTATACAATTCCTTTC	0.514	70.14	66.67
16805	CAATTCCTTTCAATTTTAT	0.364	36.69	50
16807	CAGACCAAAATTAAATAAG	0.522	62.14	41.67
16808	AGACCAAAATTAAATAAGA	0.442	45.5	41.67
16809	ACCAAAATTAAATAAGAAA	0.441	43.62	58.33
16810	CAAAATTAAATAAGAAAGT	0.436	47.57	58.33
16811	TAAGAAAGTTACATAAGAT	0.591	64.75	66.67
16812	AAGTTACATAAGATTCCAT	0.511	45.14	58.33
16814	ACATAAGATTCCATTTGAG	0.555	82.01	66.67
16815	AAGATTCCATTTGAGCATA	0.547	40.38	58.33
16816	CCATTTGAGCATACATAAG	0.440	58.72	50
16817	CATTTGAGCATACATAAGG	0.651	76.71	66.67
16818	ATAAGGCCATGATACTTTA	0.759	51.17	66.67
16819	GCCATGATACTTTAATGTG	0.624	50.72	41.67
16820	TTAATGTGAACCACCATTT	0.858	68.35	100
16821	TGTGAACCACCATTTCTTG	0.849	60.43	66.67
16823	GAACCACCATTTCTTGGAA	0.383	33.81	33.33
16824	ATTTCTTGGAAGAAAGAAG	0.537	90.92	75
16825	TGGAAGAAAGAAGACATCC	0.832	67.72	75
16826	GGAAGAAAGAAGACATCCA	0.684	41.19	50
16827	GAAAGAAGACATCCAAATG	0.736	74.46	50
16828	AAGACATCCAAATGTCCGA	0.830	57.55	66.67
16829	CATCCAAATGTCCGATTCA	0.389	45.23	50
16831	TTCCTGGCCAGTCATCCAG	0.757	69.69	66.67
16832	CCTGGCCAGTCATCCAGTA	0.552	10.79	16.67
16833	AGTCATCCAGTAGACTCTC	0.773	54.14	50
16834	AGACTCTCTCCACTCTTCA	0.707	52.79	66.67
16835	GGAAGGTGATGCTTATATT	0.518	42.45	41.67
16836	TCTACAAAGCTCACATACA	0.753	55.94	66.67
16837	CAACAAGACAGTACATCCT	0.817	44.69	58.33
16838	ACAGTACATCCTAGATTTG	0.794	54.14	58.33
16839	TCCTAGATTTGTGACTGAT	0.753	45.14	66.67
16840	TTTGTGACTGATATGAGCA	0.836	68.53	75
16841	TGTGACTGATATGAGCATT	0.760	46.49	66.67
16842	GCATTTAAGGCTGTCATTT	0.423	45.23	58.33
16843	TTTAAGGCTGTCATTTTCA	0.788	72.39	91.67
16844	TTAAGGCTGTCATTTTCAA	0.871	57.46	66.67
16846	AAGTATAAAAGTTTAGTGT	0.761	62.86	75
16847	TAAAAGTTTAGTGTTCCAT	0.821	70.86	83.33
16848	GTGTTCCATTACCCAATCT	0.720	23.38	50
16849	TGTTCCATTACCCAATCTG	0.863	80.76	75
16850	GTTCCATTACCCAATCTGT	0.471	42.45	50
16851	TGTGCAGTAGACATAGCTA	0.868	52.79	58.33
16852	TGCAGTAGACATAGCTATA	0.776	45.14	58.33
16854	TAGCTATAAGCTCAAGTCA	0.849	48.65	75
16855	ATAAGCTCAAGTCATGTGA	0.870	68.35	83.33
16856	CTCAAGTCATGTGAATTAA	0.544	38.31	58.33
16859	TATAATTTGGGCAACAGCA	0.856	66.1	83.33
16860	ATAATTTGGGCAACAGCAA	0.835	66.1	75
16861	GCAACAGCAAGTTTAAATG	0.746	67.27	50
16862	ACAGCAAGTTTAAATGTAG	0.825	71.85	58.33
16863	ATTGTTTCTCTAAAATGCA	0.849	55.67	58.33
16864	ATGCATTAGGTTGTTCACA	0.754	54.95	58.33
16865	TGCATTAGGTTGTTCACAA	0.830	51.44	66.67
16867	TAACACCATTATCTGATGC	0.928	73.38	75
16868	AACACCATTATCTGATGCA	0.616	60.34	66.67
16869	CATTATCTGATGCATCCAT	0.825	36.42	58.33
16870	TATCTGATGCATCCATTAT	0.808	62.23	83.33
16871	CTGATGCATCCATTATCTG	0.804	52.25	41.67
16873	ATCCATTATCTGATGCATG	0.723	77.52	58.33
16874	CATTATCTGATGCATGGCA	0.597	36.42	58.33
16875	TTATCTGATGCATGGCATA	0.757	66.1	75
16876	ATGCAATAAGGCAGATCCA	0.887	48.65	58.33
16877	GCAATAAGGCAGATCCACA	0.688	45.23	50
16878	CAATAAGGCAGATCCACAG	0.790	73.92	66.67
16881	CGTCCCGTAGTAGTCGTCG	0.627	54.59	33.33
16882	CCCGTAGTAGTCGTCGTAG	0.690	43.79	33.33
16883	GTAGTCGTCGTAGAAGAGG	0.827	65.02	41.67
16884	TAGTCGTCGTAGAAGAGGT	0.893	55.31	66.67
16885	GTCGTAGAAGAGGTCTGAG	0.372	60.52	33.33
16887	AGAGGTCTGAGCCCTCGTC	0.517	52.61	41.67
16888	GTCTGAGCCCTCGTCGGGC	0.336	63.4	33.33
16889	GTCGGGCGCCGGCGCCTTG	0.268	35.52	25
16891	GCGCCGGCGCCTTGGTCTT	0.255	14.93	16.67
16893	TGGTCTTCACGCAGTACTC	0.737	64.93	50
16894	GTCTTCACGCAGTACTCGG	0.590	59.89	41.67

16895	TTCACGCAGTACTCGGCCA	0.652	36.6	50
16896	CGCAGTACTCGGCCAGCGT	0.524	24.37	25
16900	GTCACGCTCCGCGTCCACC	0.534	48.47	25
16902	CCTTGGTCCCCAGGACCTG	0.430	54.95	33.33
16903	GTCCCCAGGACCTGCTTCC	0.584	55.49	41.67
16905	GCTTCCGGATGATGTCTGT	0.349	36.15	41.67
16906	GATGATGTCTGTGTACTCC	0.766	71.31	58.33
16907	TGTGTACTCCCGATCCTTC	0.743	60.43	58.33
16908	GTGTACTCCCGATCCTTCC	0.565	57.1	50
16909	CCCGATCCTTCCCCTTGCT	0.412	0	16.67
16911	TTTCCACTTCCTGTACATC	0.829	73.38	58.33
16913	CCTGTACATCACGGAGGCG	0.364	33.36	25
16914	CATCACGGAGGCGTCCACG	0.794	67.54	41.67
16915	TCACGGAGGCGTCCACGTT	0.552	45.5	50
16916	CGGGCGAGAAGGTGTTGGG	0.459	42.54	25
16917	GTTGGGCTCGTTCAGGAGG	0.682	46.31	25
16918	TCGTTCAGGAGGGAGATCA	0.700	47.39	75
16920	GGAGGGAGATCACACTCAG	0.232	51.08	25
16921	GAGGGAGATCACACTCAGG	0.584	50.45	25
16922	GGGAGATCACACTCAGGAG	0.423	44.15	25
16923	TCACACTCAGGAGAATGGT	0.813	46.49	66.67
16924	CACACTCAGGAGAATGGTC	0.449	45.95	25
16925	CAGGAGAATGGTCCTGACG	0.721	53.24	33.33
16926	AGGAGAATGGTCCTGACGT	0.722	51.44	41.67
16927	CTGACGTTCTGCGTGGGGT	0.578	40.83	33.33
16928	TCTGCGTGGGGTTCCACCT	0.726	42.72	50
16929	CCACCTCTCTGAGGGCAGC	0.393	33.36	16.67
16935	GGTCGTCCACCGGCGGGTG	0.254	33.36	16.67
16936	GTCGTCCACCGGCGGGTGG	0.442	35.52	33.33
16937	CGTAGATGTTAGGGTGCCA	0.251	36.15	33.33
16938	TGTTAGGGTGCCACATCTT	0.367	53.15	75
16939	GTGGAGAGTATGGGTAGTC	0.659	59.26	41.67
16940	GTATGGGTAGTCGATGGGG	0.449	67.9	41.67
16942	GATGGGGAACTTGAGGCGC	0.580	61.24	33.33
16943	ATGGGGAACTTGAGGCGCG	0.511	70.23	41.67
16945	CGCCTTGAAGTAGCCGCCC	0.298	37.5	25
16947	GAAGTAGCCGCCCTCGTAG	0.598	67.54	50
16948	AAGTAGCCGCCCTCGTAGT	0.737	36.6	50
16949	CCTCCCAGTTGTATAGATC	0.417	61.15	33.33
16950	TCCCAGTTGTATAGATCGC	0.740	58.27	58.33
16951	GTTGTATAGATCGCCCTCG	0.727	67.27	58.33
16952	TGTATAGATCGCCCTCGTC	0.643	75.72	66.67
16954	TCGCCCTCGTCCACCAGTG	0.755	54.5	41.67
16955	CGTCCACCAGTGTCACGCG	0.673	33.36	16.67
16957	CCCTCGACCGGCTCTTCCT	0.443	24.37	25
16958	CGGCTCTTCCTGCAGCCCC	0.476	37.5	25
16959	GTCTGTTTTATAGATCCGT	0.683	40.56	50
16960	TTTATAGATCCGTGCAATC	0.924	88.67	83.33
16961	AGATCCGTGCAATCTCTGG	0.820	75.72	58.33
16962	CGTGCAATCTCTGGCACTA	0.624	32.28	25
16963	TGCAATCTCTGGCACTAGG	0.862	50	50
16964	GGGTCATCTGGGTTTGGAT	0.445	31.65	25
16965	GGTCATCTGGGTTTGGATC	0.565	43.44	25
16966	CTGGGTTTGGATCACATAG	0.958	54.23	41.67
16967	GGGTTTGGATCACATAGCA	0.422	25.36	41.67
16968	TTGGATCACATAGCAGTGA	0.952	35.97	75
16969	CATAGCAGTGAACAAATGG	0.945	74.46	58.33
16970	ATAGCAGTGAACAAATGGA	0.984	61.69	66.67
16971	TAGCAGTGAACAAATGGAT	0.961	50.9	75
16972	GCAGTGAACAAATGGATAA	0.236	34.53	58.33
16973	GTGAACAAATGGATAAAAG	0.270	69.96	50
16974	TGGATAAAAGAACTTTAGA	0.642	47.66	58.33
16975	GATAAAAGAACTTTAGAAA	0.306	56.65	58.33
16976	AAAAGAACTTTAGAAATTG	0.806	87.68	66.67
16977	TTTAGAAATTGTTAAAGCA	0.920	65.11	66.67
16978	TTAGAAATTGTTAAAGCAG	0.897	81.03	66.67
16979	ATTGTTAAAGCAGGCGACC	0.845	84.8	66.67
16980	TTGTTAAAGCAGGCGACCA	0.903	58.09	75
16981	TGTTAAAGCAGGCGACCAC	1.002	80.76	75
16982	GGCGACCACTGTGATCTTA	0.556	3.78	25
16983	CGACCACTGTGATCTTAGA	0.433	14.57	25
16985	TGATCTTAGAATATCGAGA	0.981	61.69	75
16986	GAATATCGAGACAAATGCT	0.792	34.17	50
16987	AATATCGAGACAAATGCTG	0.952	90.92	83.33
16988	ATCGAGACAAATGCTGCCA	0.996	53.69	66.67
16989	ACAAATGCTGCCATTACTG	0.974	82.01	66.67
16990	TGCTGCCATTACTGTTAAT	0.576	27.43	50
16991	CTGTTAATATTTGGATGAT	0.320	37.32	41.67
16992	TAATATTTGGATGATAAAT	0.762	62.32	75
16993	TTTGGATGATAAATTCTTG	0.931	84.53	66.67
16994	GATAAATTCTTGTTGTAAA	0.488	51.62	50
16995	ATAAATTCTTGTTGTAAAT	0.744	62.32	66.67

16996	TAAATTCTTGTTGTAAATG	0.700	76.26	66.67
16997	AATTCTTGTTGTAAATGCA	0.833	62.32	58.33
16998	ATTCTTGTTGTAAATGCAA	0.684	55.67	58.33
16999	CTTGTTGTAAATGCAACCT	0.866	44.69	58.33
17000	TTGTTGTAAATGCAACCTT	0.830	57.55	83.33
17001	GTTGTAAATGCAACCTTAG	0.809	67.81	58.33
17002	CAACCTTAGGTGGTTTGAA	0.644	48.74	41.67
17003	ACCTTAGGTGGTTTGAAGG	0.945	71.22	58.33
17004	GGTGGTTTGAAGGGGTAGT	0.631	31.74	33.33
17005	GTGGTTTGAAGGGGTAGTC	0.876	56.47	41.67
17006	TTGAAGGGGTAGTCTGTAG	0.949	77.88	83.33
17007	TGAAGGGGTAGTCTGTAGG	0.999	84.26	66.67
17008	TAGTCTGTAGGAAAATGAA	0.850	55.31	66.67
17009	GTCTGTAGGAAAATGAATT	0.623	49.64	50
17010	CTGTAGGAAAATGAATTGT	0.762	40.56	66.67
17011	TGTAGGAAAATGAATTGTC	0.923	80.76	75
17012	GTAGGAAAATGAATTGTCA	0.554	51.53	50
17013	AAAAGAATACACCGCCTTG	1.045	91.46	75
17014	AGAATACACCGCCTTGATA	0.995	44.51	58.33
17015	CTTGATATGGGCTGTCATT	0.800	45.23	50
17016	TTGATATGGGCTGTCATTA	0.977	61.6	83.33
17017	GGGCTGTCATTAGGTCCCA	0.486	14.93	25
17018	GCTGTCATTAGGTCCCATA	0.444	34.53	50
17019	TGTCATTAGGTCCCATAAT	0.641	46.49	66.67
17020	GGTCCCATAATTGTGGCTT	0.520	40.83	41.67
17021	GTCCCATAATTGTGGCTTG	0.352	54.23	41.67
17022	TCCCATAATTGTGGCTTGC	0.758	61.06	66.67
17023	CCCATAATTGTGGCTTGCC	0.602	50.72	33.33
17024	TAATTGTGGCTTGCCAATG	1.003	88.67	83.33
17025	TTGTGGCTTGCCAATGAAA	0.853	40.38	58.33
17026	TGTGGCTTGCCAATGAAAC	0.949	75.36	58.33
17027	TGGCTTGCCAATGAAACAT	0.907	37.95	66.67
17028	GGCTTGCCAATGAAACATA	0.447	12.68	41.67
17029	GCCAATGAAACATATCATC	0.661	50.18	50
17030	CAATGAAACATATCATCCC	0.950	76.71	58.33
17031	ATATCATCCCCAACTGGAC	0.883	88.67	66.67
17032	TATCATCCCCAACTGGACC	0.937	73.38	58.33
17033	CCCCAACTGGACCTGCAGA	0.329	6.29	16.67
17034	CCCAACTGGACCTGCAGAA	0.500	23.83	41.67
17035	GACCTGCAGAACATTGTGC	1.003	47.84	41.67
17036	ACCTGCAGAACATTGTGCT	1.015	53.69	58.33
17037	CCTGCAGAACATTGTGCTG	0.878	52.07	25
17038	CTTTGTTCAACAATGGCCG	0.765	80.22	58.33
17039	GTTCAACAATGGCCGAAAC	0.616	50.09	41.67
17040	CAATGGCCGAAACTCTTTT	0.492	42.72	58.33
17041	ATGGCCGAAACTCTTTTCT	0.778	57.19	66.67
17042	TGGCCGAAACTCTTTTCTC	0.827	67.36	50
17043	CGAAACTCTTTTCTCATAT	0.463	42.45	58.33
17044	AAACTCTTTTCTCATATTC	0.787	78.24	66.67
17045	ACTCTTTTCTCATATTCTC	0.836	69.06	58.33
17046	CTCTTTTCTCATATTCTCG	0.735	67.18	58.33
17047	TCTTTTCTCATATTCTCGT	0.842	46.76	75
17048	ATTCTCGTTTGTTTTCCTG	0.840	88.31	66.67
17049	TTTGTTTTCCTGATAAAGC	0.804	88.31	75
17050	TTTCCTGATAAAGCTGTGC	0.838	84.26	83.33
17051	TCCTGATAAAGCTGTGCTG	0.775	67.18	66.67
17052	GATAAAGCTGTGCTGCCTG	0.751	77.97	50
17053	AAAGCTGTGCTGCCTGGCT	0.714	55.67	50
17054	AGCTGTGCTGCCTGGCTAT	0.712	38.58	50
17055	GCTGTGCTGCCTGGCTATT	0.510	10.79	25
17056	TGCTGCCTGGCTATTGGCT	0.759	29.95	41.67
17057	CCTGGCTATTGGCTGGACT	0.687	32.01	25
17058	GGACTGTTAGGATTCGGTT	0.499	35.79	41.67
17059	GACTGTTAGGATTCGGTTC	0.727	60.88	50
17060	TTAGGATTCGGTTCATCCA	0.743	59.44	66.67
17061	AGGATTCGGTTCATCCAGC	0.815	56.29	41.67
17062	GGTTCATCCAGCAGAGACT	0.531	38.4	41.67
17063	TTCATCCAGCAGAGACTGA	0.624	40.38	75
17064	ATCCAGCAGAGACTGAATT	0.608	42.27	58.33
17065	CTGAATTGATGTTAAGATA	0.505	34.53	41.67
17066	TTAAGATAGAAGATACATC	0.847	93.44	75
17067	ATAGAAGATACATCATATG	0.839	80.49	66.67
17068	ATACATCATATGTTGGACT	0.836	53.06	66.67
17069	ATGTTGGACTCCATCGATT	0.744	61.6	66.67
17070	TGTTGGACTCCATCGATTC	0.841	78.51	75
17071	GTTGGACTCCATCGATTCT	0.648	27.52	41.67
17072	TTGGACTCCATCGATTCTG	0.863	73.47	75
17073	GACTCCATCGATTCTGAAG	0.722	63.67	41.67
17074	CTCCATCGATTCTGAAGGA	0.500	16.73	33.33
17075	TCCATCGATTCTGAAGGAT	0.772	42.36	66.67
17076	CGATTCTGAAGGATATCTA	0.479	44.69	58.33
17077	CTGAAGGATATCTAAACAT	0.467	40.56	58.33

17078	TGAAGGATATCTAAACATA	0.635	52.16	58.33
17079	TCTAAACATATGCTACCAT	0.694	46.76	75
17080	TATGCTACCATCAGCATAC	0.757	87.05	83.33
17081	CCATCAGCATACACATTTG	0.626	58.72	50
17082	TCAGCATACACATTTGGAT	0.730	55.04	66.67
17083	GCATACACATTTGGATGAA	0.460	38.94	50
17084	CACATTTGGATGAAACATT	0.635	40.56	58.33
17085	TTTGGATGAAACATTTTGG	0.846	90.56	83.33
17086	GGATGAAACATTTTGGATA	0.482	47.48	50
17087	AAAACCTAACAGTTGGTGG	0.697	94.96	75
17088	AGTTGGTGGTTTATTTGGA	0.618	59.44	58.33
17089	GTTTATTTGGATATTCTTC	0.777	74.19	58.33
17090	TTATTTGGATATTCTTCAG	0.801	84.89	75
17091	TTTGGATATTCTTCAGAAA	0.825	55.67	58.33
17092	TTGGATATTCTTCAGAAAA	0.853	47.66	58.33
17093	TGGATATTCTTCAGAAAAT	0.637	38.58	58.33
17094	TCAGAAAATTCTATTACTA	0.723	51.8	58.33
17095	AAAATTCTATTACTAGTTT	0.475	53.69	66.67
17096	AATTCTATTACTAGTTTAA	0.467	67.36	75
17097	ATTACTAGTTTAAAAGTAC	0.862	87.68	66.67
17098	ATCTTCAAAAGGTGTCCCT	0.781	60.34	75
17099	CTTCAAAAGGTGTCCCTTC	0.798	67.81	58.33
17100	TTCTGGTCCAAATATAACT	0.790	57.55	75
17101	TGGTCCAAATATAACTGCA	0.817	45.14	58.33
17102	GGTCCAAATATAACTGCAT	0.532	34.53	41.67
17103	AAATATAACTGCATTCCAC	0.846	97.75	75
17104	ATATAACTGCATTCCACTG	0.835	73.74	66.67
17105	CATTCCACTGCATGATGTT	0.550	51.89	50
17106	TCCACTGCATGATGTTGTT	0.564	42.36	58.33
17107	GCATGATGTTGTTTTCAGA	0.483	42.45	41.67
17108	GATGTTGTTTTCAGATGGT	0.508	42.45	50
17109	TGTTGTTTTCAGATGGTGC	0.742	82.01	75
17110	TTGTTTTCAGATGGTGCGC	0.728	77.88	75
17111	TTCAGATGGTGCGCCACTG	0.618	74.1	58.33
17112	GCCACTGACACCCACAGGT	0.767	23.83	33.33
17113	CACAGGTGGGTCCTCTTGT	0.611	40.83	41.67
17114	CTCTTGTAACCGCTTGAAA	0.455	44.6	50
17115	TCTTGTAACCGCTTGAAAT	0.782	62.23	66.67
17116	GTAACCGCTTGAAATCCCG	0.851	71.67	41.67
17117	GGAGCCGATGTAGCCACCC	0.581	48.29	25
17118	AGCCGATGTAGCCACCCCG	0.543	56.74	41.67
17119	GATGTAGCCACCCCGCATG	0.677	63.49	50
17120	GCATGGAGCGCTGCACGTT	0.612	35.16	33.33
17121	TTCTGCTCAAACAGCCGCC	0.766	76.35	66.67
17122	AACAGCCGCCGGTTGTTCT	0.612	42.9	50
17123	AGCCGCCGGTTGTTCTGCA	0.676	23.29	25
17124	GCCGCCGGTTGTTCTGCAG	0.641	37.5	16.67
17125	TGTTCTGCAGGACCTCTGC	0.699	71.94	66.67
17126	TTCTGCAGGACCTCTGCGG	0.671	79.14	66.67
17127	CTGCAGGACCTCTGCGGCC	0.664	50.45	33.33
17128	TGCAGGACCTCTGCGGCCT	0.654	41.37	50
17129	TCTGCGGCCTCCTTGTTCA	0.659	49.01	58.33
17130	CTGCGGCCTCCTTGTTCAG	0.686	35.52	16.67
17131	CTCCTTGTTCAGTGGGTCC	0.631	50.45	41.67
17132	CAGTGGGTCCTCGGGGTTG	0.628	57.1	41.67
17133	CTCGGGGTTGGGCTCCAAG	0.645	56.74	25
17134	GGGGTTGGGCTCCAAGAAG	0.613	37.5	25
17135	GGGTTGGGCTCCAAGAAGA	0.561	21.58	33.33
17136	GTTGGGCTCCAAGAAGAGA	0.697	27.52	25
17137	TTGGGCTCCAAGAAGAGAT	0.771	50.9	66.67
17138	AGAAGAGATACTGCAGGCC	0.802	77.97	58.33
17139	TACTGCAGGCCATAAATTA	0.670	58.09	75
17140	GGCCATAAATTATGGAGTT	0.497	21.49	33.33
17141	AAATTATGGAGTTTATCGT	0.822	70.86	75
17142	TGGAGTTTATCGTAAGGAC	0.767	64.93	58.33
17143	GGAGTTTATCGTAAGGACT	0.606	29.5	41.67
17144	GTTTATCGTAAGGACTGGC	0.685	58.99	58.33
17145	AGGACTGGCTTCCAGTCCT	0.541	38.58	33.33
17146	AGGATGTTGAGGCAGACGT	0.804	44.6	58.33
17147	TTGAGGCAGACGTTGCCCT	0.795	45.14	58.33
17148	CGAGGTCAATGTTGGGGTG	0.625	33.36	16.67
17149	GAGGTCAATGTTGGGGTGA	0.654	34.44	41.67
17150	AATGTTGGGGTGATAGACC	0.797	88.31	66.67
17151	TGTTGGGGTGATAGACCAT	0.713	53.15	66.67
17152	TTGGGGTGATAGACCATTG	0.861	71.22	66.67
17153	GGGGTGATAGACCATTGTC	0.618	44.06	25
17154	TAGACCATTGTCTCACACT	0.921	58.09	66.67
17155	CATTGTCTCACACTTCACC	0.817	65.29	50
17156	CATGCGGGTAACCCTGGCC	0.738	63.49	41.67
17157	ATGCGGGTAACCCTGGCCC	0.793	75.99	50
17158	GCGGGTAACCCTGGCCCAC	0.564	40.29	16.67
17159	GTAACCCTGGCCCACCTTA	0.699	30.4	41.67

17160	GCCCACCTTAAAACTGAAC	0.539	28.6	33.33
17161	CCTTAAAACTGAACACAAA	0.511	38.94	50
17162	ACACAAACTTCCCACTCTT	0.851	49.28	58.33
17163	AAACTTCCCACTCTTGTAG	0.873	75.63	75
17164	ACTTCCCACTCTTGTAGAA	0.689	38.22	50
17165	CTTCCCACTCTTGTAGAAG	0.759	74.1	41.67
17166	TCTTGTAGAAGCCCTCATC	0.827	80.76	75
17167	TGTAGAAGCCCTCATCAGG	0.779	78.51	58.33
17168	TAGAAGCCCTCATCAGGAC	0.803	62.95	58.33
17169	CCCTCATCAGGACAGATGA	0.575	25.36	33.33
17170	CCTCATCAGGACAGATGAC	0.589	37.14	25
17171	CATCAGGACAGATGACCAG	0.767	67.27	50
17172	ACAGATGACCAGCTTGAAG	0.770	75.36	58.33
17173	GTCGTCTGGATCTGAGAAG	0.656	56.47	41.67
17174	GTCTGGATCTGAGAAGCTG	0.793	63.67	33.33
17175	GATCTGAGAAGCTGATATC	0.605	70.05	58.33
17176	TCTGAGAAGCTGATATCAC	0.703	78.15	66.67
17177	GTCTTGGGCAGGTTCAGCT	0.718	43.08	50
17178	CTTGGGCAGGTTCAGCTCG	0.719	46.31	33.33
17179	TTGGGCAGGTTCAGCTCGT	0.660	47.66	50
17180	CGTTTATGTCCTTCTGGAT	0.564	36.15	50
17182	GATCCGCAGCTGCGCCGCC	0.717	46.31	25
17183	ATCCGCAGCTGCGCCGCCG	0.699	70.23	41.67
17184	AGCTGCGCCGCCGACGCCT	0.517	38.58	41.67
17185	CCGACGCCTTCTTGCTGCT	0.530	6.65	16.67
17186	GACGCCTTCTTGCTGCTGC	0.610	56.74	33.33
17187	CGCCTTCTTGCTGCTGCCC	0.589	22.57	16.67
17188	GCTGCCCTTGGTGCCGCCC	0.649	33.36	16.67
17191	TTGGTGCCGCCCGCCGACT	0.736	29.95	50
17192	TGGTGCCGCCCGCCGACTC	0.530	46.22	41.67
17193	TGCCGCCCGCCGACTCCTC	0.599	39.57	33.33
17194	CCCGCCGACTCCTCCTCCT	0.417	14.93	16.67
17195	GACTCCTCCTCCTTCTTCT	0.594	44.6	41.67
17196	ACTGGAAGTCTGTGTCCGT	0.857	49.28	41.67
17197	GTCTGTGTCCGTGAGGGCG	0.591	57.1	33.33
17198	CTCATCTTGGGATGAGGAT	0.538	38.31	50
17199	GGGGCTCATCCCCTGAGCT	0.430	6.29	8.33
17200	CCTGAGCTCCCAGGAGCCC	0.363	33.36	25
17201	CTCCCAGGAGCCCCATCCC	0.433	50.45	25
17202	CATCCCCGTCCACCTCTGA	0.295	23.74	33.33
17203	TCTGAGTCCTGCAGCACCT	0.618	42.72	58.33
17204	ATTTCGCAGATTTGGGGAC	0.820	75.99	66.67
17205	TTGCTTCTGGGGTCCACAG	0.754	52.52	50
17206	TCTTGTAGTTGTGGGCACT	0.817	55.94	66.67
17207	CACTGAGGTATTTGAAGTG	0.550	63.13	50
17208	CAGACGTGGCAGAGACAGA	0.683	34.53	33.33
17209	ACATAGACGTGGGAGCTAG	0.667	78.51	66.67
17210	ATAGACGTGGGAGCTAGGA	0.797	59.44	58.33
17211	ACGTGGGAGCTAGGAACCC	0.579	61.15	41.67
17212	CAGAAGAGGCAGAGGATGG	0.532	59.89	50
17213	CTTCTTGTCCAGCTTGGCC	0.650	67.54	41.67
17214	TGTCCAGCTTGGCCGGCAG	0.652	65.29	50
17215	CTACCAGGTGCTTCAGCAG	0.750	61.24	33.33
17216	TACCAGGTGCTTCAGCAGC	0.824	67.45	58.33
17217	ATGACCACCGTGAAGACGC	0.759	76.89	50
17218	GCGTAGGCTGCGCCAAACT	0.609	21.58	33.33
17219	TGATGCCGCTTCACGTTGC	0.724	57.01	58.33
17220	GCTTCACGTTGCGCCTCTC	0.568	40.02	25
17221	TTGCGCCTCTCGGCGGCCG	0.729	52.52	41.67
17222	GGATCATGTACATGAGCAG	0.490	60.97	50
17223	TCATGTACATGAGCAGGCC	0.719	78.51	58.33
17224	AGCAGGTCCATGGCGTTGC	0.711	63.4	58.33
17225	TCCATGGCGTTGCCGATAA	0.627	42.36	66.67
17226	GAAAGTGTAGCACTTGTGC	0.836	77.97	58.33
17227	AAAGTGTAGCACTTGTGCA	0.991	65.74	66.67
17228	GGAGGGCCGAGAAGCACCA	0.651	13.04	25
17229	GGTGGGGAAGAACTGGGTG	0.561	54.59	25
17230	TCCATGGTGGTGATGATGA	0.792	48.65	75
17231	CCATGGTGGTGATGATGAT	0.595	36.15	41.67
17232	ATGATGATGGCTGGAGTGG	0.774	80.67	66.67
17233	GATGATGGCTGGAGTGGTG	0.702	61.24	41.67
17234	TGGTGAGGAAGAAGAGGAG	0.680	67.18	58.33
17235	AGGAGGATGAAGAGGACGA	0.558	47.39	50
17236	AGATGAAGCCTCGGATGGA	0.653	55.94	50
17237	GGCGTGGCTCCCCACGGCA	0.627	14.93	25
17238	ATTTACACACGTTGAAGTC	1.016	91.46	75
17239	CACGTTGAAGTCCTTCAGG	0.845	60.52	33.33
17240	ATAGTCTCATTGTGGAAGG	0.723	82.01	66.67
17241	TTGTGGAAGGTGACAAAGG	0.742	80.67	66.67
17242	TCAATGGCCTCCACCTGCT	0.772	49.37	66.67
17243	CCACCTGCTCACAGCCTCG	0.422	48.29	33.33
17244	TGATCATGGTAGGCACGTT	0.801	53.15	66.67

17245	GGAGGTTTGTGAAGTACAG	0.574	52.07	33.33
17246	CTCGGCCTTCTTCCTCTCT	0.494	12.95	25
17247	CTTCTTCCTCTCTGCATCG	0.564	50.09	41.67
17248	ATCGAGGAACATTAGGCGA	0.779	48.65	58.33
17249	CGAGCCACGTTGTAACACG	0.916	51.08	25
17250	AGCCACGTTGTAACACGGG	0.758	54.5	41.67
17251	TGTGCAGTTGGGGTAGGCT	0.707	49.01	50
17252	CAAAATGCTTCTTGATCTT	0.617	49.1	58.33
17253	GCATATTTGGAGATTCCAT	0.787	42.45	50
17254	GATCATCCTCCTTGTAGCG	0.793	50.09	33.33
17255	TGGAGGTGTGTCTACGCAT	0.768	42.36	50
17256	GACGGTGAGCAGCAGATAC	0.654	50.45	33.33
17257	GTTGTTCCCTGATTTCAAG	0.711	56.38	41.67
17258	ATGGTGGTTCTCCCAAAGC	0.758	71.22	50
17259	GTTCTCCCAAAGCTGTAGG	0.819	58.63	41.67
17260	GCACGATGCCTACGGAGAG	0.758	48.29	25
17262	CAGCAGCCCGATGATGTGC	0.699	35.52	33.33
17264	AAGGACAGGTAGTGCACGG	0.868	70.23	50
17265	GTCCCGGATCTCATCATCC	0.893	56.74	33.33
17266	CGGATCTCATCATCCTTTA	0.247	25.36	50
17268	TGTCCCTTTGGTCAAAGTC	0.895	69.06	58.33
17269	TTGGTCAAAGTCAACGGAG	0.986	74.01	66.67
17270	AGTCAACGGAGCTGGAGAC	0.695	52.61	50
17271	CTGGAGACAGAGGTGAGAC	0.886	59.53	33.33
17272	TGAGACGCTCATACCGGTC	0.990	65.29	58.33
17273	TCATACCGGTCATGGCTGT	0.564	38.22	66.67
17274	GGCTGTCCCCGTGCATAGC	0.732	29.23	16.67
17275	GGAAGTCTGTGTCCGTGAG	0.617	40.02	25
17276	GTGAGGGCGTCGGGTGGCA	0.127	34.53	33.33
17278	CGTCGGGTGGCATCAGCAA	0.397	25.72	25
17279	AACTCCTCATCTTGGGATG	0.530	77.88	58.33
17280	ATCCCCTGAGCTCCCAGGA	0.520	44.87	41.67
17281	GTCCTGCAGCACCTGAGCG	0.462	41.82	25
17282	CCTGAGCGATGTATTTCGC	0.402	43.44	25
17283	AGCGATGTATTTCGCAGAT	0.505	35.7	50
17284	TTCTGGGGTCCACAGTATC	0.738	77.88	58.33
17285	AGTTGTGGGCACTGAGGTA	0.513	53.15	50
17287	TGACGATGGTGATGACCAG	0.670	69.06	50
17288	CGTGCGCATGGTGGAAAAG	0.000	33.36	16.67
17289	GCGCATGGTGGAAAAGAAG	0.397	41.28	25
17290	GAGGATGGGCGCGGCCACC	0.494	50.45	33.33
17292	GGATGGGCGCGGCCACCAC	0.463	54.95	33.33
17294	GGCGCGGCCACCACCTGGT	0.576	17.18	25
17295	GCAGGTAGGCGTAGTAGAG	0.564	51.08	25
17296	ACGATGATGGGGCAGGTGA	0.730	41.37	58.33
17298	GGTGATACTGTAGGTCATG	0.589	54.86	41.67
17299	GTGATACTGTAGGTCATGA	0.602	29.68	50
17300	ACATCATCCAGGCGTAGGC	0.723	74.19	58.33
17301	CCGCTTCACGTTGCGCCTC	0.595	22.57	16.67
17302	CGCTTCACGTTGCGCCTCT	0.357	24.37	41.67
17303	ACGTTGCGCCTCTCGGCGG	0.218	46.22	41.67
17304	GCAGAGCCGGATCATGTAC	0.468	33.36	33.33
17305	GATCATGTACATGAGCAGG	0.731	65.02	50
17306	ATGTACATGAGCAGGCCTG	0.721	82.91	66.67
17307	TGAGCAGGCCTGGGATGCG	0.749	65.29	50
17308	GCAGCAGGTCCATGGCGTT	0.272	25.72	25
17309	CCATGGCGTTGCCGATAAA	0.367	21.22	25
17310	ACCATGAAGATGAGGAAAG	0.727	69.96	66.67
17311	CTTGTGCATGGTTGTCCTG	0.615	50.09	33.33
17312	ATGGTTGTCCTGTTCTCCC	0.659	77.52	50
17313	TTGTCCTGTTCTCCCCAGA	0.680	55.31	58.33
17314	CCTGTTCTCCCCAGAGCGT	0.483	10.79	25
17315	AGCACCACAGCAGCAGGGT	0.585	41.37	41.67
17317	AGAACTGGGTGATGATGGG	0.687	75.72	50
17318	AACTTGTCCATGGTGGTGA	0.677	63.85	83.33
17319	GTGGTGATGATGATGGCTG	0.743	65.56	41.67
17320	AAGAAGAGGAGGATGAAGA	0.584	66.64	75
17321	TGAAGAGGACGACATTGAT	0.492	53.15	66.67
17322	GACGACATTGATGACCAGG	0.604	57.01	33.33
17323	TGATGACCAGGCAGCGCAG	0.661	57.01	50
17324	GCAGCGCAGCCACCAGATG	0.293	33.36	16.67
17325	CACCAGATGAAGCCTCGGA	0.292	32.91	41.67
17326	TGTGCAGGGACTCGCTGCA	0.259	44.96	58.33
17327	CAGCCCTGGCATTTACACA	0.668	37.95	25
17328	GCCCTGGCATTTACACACG	0.521	37.5	25
17329	TGTGGAAGGTGACAAAGGC	0.725	71.85	50
17331	CCAAGAGGCTTCTCATTCA	0.395	36.15	50
17332	CAAGAGGCTTCTCATTCAC	0.693	65.02	50
17333	TCCTTCTCCCGCTTGTAGT	0.720	48.65	58.33
17334	CTCCAGCTTTGTGTAGTAC	0.584	45.59	41.67
17336	GGGGTTGATCATGGTAGGC	0.396	37.5	25
17337	TGGTAGGCACGTTCTCCTT	0.702	42.36	58.33

17339	GGGTAGGCTTCCTCAAAAT	0.278	25.36	41.67
17340	GTAGGCTTCCTCAAAATGC	0.873	65.02	50
17342	GATTCCATTGATGAAGAGG	0.739	74.46	50
17343	CCGCTTCACCAGATCATCC	0.639	28.87	25
17344	TTCACCAGATCATCCTCCT	0.774	58.09	66.67
17345	GAGCAGCAGATACAGGAAG	0.758	41.55	33.33
17346	TTGTTCCCTGATTTCAAGT	0.344	46.67	66.67
17347	CAAGTTGGCAATGGTGGTT	0.342	44.69	58.33
17348	GGCAATGGTGGTTCTCCCA	0.438	21.58	33.33
17349	GAGAAGTTGACAGGCAGCA	0.605	40.56	50
17350	AGAGGACGCCCACAACCAC	0.535	50.36	33.33
17351	GATGTGCCGCTGAAAGGAC	0.651	46.31	33.33
17352	ACCATTGTCCCTTTGGTCA	0.709	48.65	66.67
17353	GTCAAAGTCAACGGAGCTG	0.844	63.13	50
17435	CAGCACACGAATCAAGGAA	0.221	36.69	50
17436	AGCCAGCAGGGCAATCCAG	0.637	39.57	33.33
17439	TAATGTCTCATAATAGGTT	0.663	55.94	66.67
17442	CTCCACAGAGCAACAGCGT	0.607	30.67	33.33
17443	CTCAGGCTTATGGAAGTGC	0.787	48.2	41.67
17445	GCTGGGATGTGAGCAGCTC	0.426	51.08	25
17446	CACATACTCCACCAGCCAT	0.719	23.38	33.33
17450	GTAAGCTTCCCCAAGTTCT	0.882	49.1	58.33
17451	CTTCCCCAAGTTCTGGCAC	0.419	52.61	25
17452	TGTCTCACCCAATTCTCCT	0.705	55.58	66.67
17453	TCTTGCTTGGTACGATCAG	0.547	75.72	66.67
17455	TAATCGACGGCATTGAAAG	0.768	97.75	75
17456	CGACGGCATTGAAAGACAG	0.474	37.14	16.67
17458	TCCCCGATGTTCTGGATGT	0.444	38.49	50
17459	GAATCAGGGAGATCCTCAG	0.606	73.92	50
17461	GGGACCATAAGGCTGATTT	0.419	36.69	50
17462	ACCATAAGGCTGATTTCCC	0.611	74.01	58.33
17463	TTATTGCGGCATTCCTTCA	0.777	51.17	83.33
17464	ACAGTTTGGGCTGGCGGAT	0.409	42.72	50
17465	TGGATGTACTTCTCCCTCT	0.807	42.36	75
17466	CACTTGTTGCTGGCTGGCC	0.737	57.1	41.67
17468	CCTTGTCCAATCTTCATGT	0.705	29.77	50
17469	GAATTGCAGTATGGTCAAT	0.459	34.17	50
17470	GATGTACGGAGAGTAGGTG	0.547	58.63	41.67
17471	GTAGGTGTCCGAACATTCA	0.490	42.45	50
17472	TTATTTTGTTGTCAATGAA	0.978	62.32	75
17473	TGAAAGATGCAAACATCTG	0.753	78.51	75
17474	AGATGCAAACATCTGGGTC	0.719	84.8	58.33
17476	CTTGAGAGGAAGGGCAGGT	0.532	43.71	50
17478	GTTTTGCATTTGCTCCCTG	0.679	63.04	50
17479	TTTGCATTTGCTCCCTGTT	0.492	59.44	66.67
17480	TATCCTGGCTGGGTTGGAT	0.518	65.74	58.33
17481	GTAAATCCTGAGTTCTTCT	0.447	40.47	58.33
17482	TTTCCAGGCTCACCCCAGT	0.371	55.67	58.33
17483	TTCCAGGCTCACCCCAGTT	0.584	44.87	58.33
17484	CAAGGCTTGCAGCCGGGCA	0.564	38.67	33.33
17485	CGTAGGAATGCAAAGGGGA	0.447	38.94	33.33
17486	GGCCCGCAGCCTCTTCAGC	0.374	28.87	8.33
17487	TTCAGCTTGGAGGCACTCT	0.669	55.31	66.67
17488	GTTGGGGAACTGTGGCAAG	0.506	61.24	33.33
17489	GGCAAGTCCTCTGGCAACT	0.717	21.58	33.33
17490	CACAAAGCAGAGGTTAGCC	0.702	67.18	41.67
17491	CAGAGGTTAGCCTCTTGAG	0.523	60.88	33.33
17493	AGTCATCAGTCTCTGGTAA	0.288	37.86	58.33
17495	TGGCCCATAGACCCCAGAA	0.632	34.71	41.67
17496	AGGAGGTGGGAGCGGCACC	0.390	61.15	41.67
17497	TGCCTGGTGGAGTTGCTCC	0.621	60.79	58.33
17498	TCCAGCACGCTCCGACATG	0.608	63.94	50
17500	TGCACTTAGAGACGTAGAG	0.683	67.18	66.67
17502	TGTGAGGGCAAACCCAAAT	0.722	48.74	75
17503	TGTGATAATAAAGGCATGG	0.725	74.1	83.33
17504	AAGGCATGGAATTGGGGCT	0.576	50.9	50
17505	CTGGGATCAGCCTGGGTCT	0.751	27.88	33.33
17507	CCTACTGCATCTTGGTCAA	0.579	36.15	41.67
17508	AAAATTTTCTCCTTCAGTC	0.908	94.96	75
17509	AAGGGCTGGCACCATCCCT	0.837	44.87	41.67
17510	CCATCCCTGGCTTTAGAAG	0.464	50.09	25
17511	ACTGGCATAATGCCGACAG	0.730	74.1	50
17512	GTGATGTTGAACTCCTGCA	0.625	40.56	58.33
17513	AATGTCTGCAGCACACGAA	0.402	61.69	75
17515	GAGGTGATCTCTGGCTCCC	0.497	53.24	33.33
17516	ATCGGCAGAAGTTCCGGGC	0.709	72.48	50
17517	AGTGCTTCACAATGCCATT	0.605	46.49	50
17519	GACTGCTGCAGCGGCGGGG	0.618	59.35	41.67
17520	TCCGGCATGGCCTCTCATC	0.630	57.28	41.67
17521	ATGGCCTCTCATCCACCTC	0.744	67.45	41.67
17522	CAGCTCCCCAACTAGGATC	0.666	37.77	33.33
17523	GCTTCCATCATCCATGCCC	0.544	59.98	41.67

17524	TCCACCAGCCATTTGGCAT	0.695	51.44	50
17526	ATCCATGGGTTGGCAGTGA	0.578	48.65	58.33
17527	AATCGACGGCATTGAAAGA	0.527	44.51	50
17528	GAAAGACAGGAGGTGATAG	0.631	63.04	50
17529	GGATGTGCCTCATATCCTG	0.467	58.72	33.33
17530	GGAGGCATGAGTGAGCTGG	0.644	53.33	41.67
17531	CCTACGTTCTGAATCAAGA	0.291	42.45	41.67
17532	TTTCTCTGCCGGTTGTCCT	0.670	65.74	66.67
17533	AACAGGTGGGACCATAAGG	0.625	77.88	50
17535	GATCCTTTCCAGGAGATCA	0.441	42.45	50
17536	TTTCCAGGAGATCACAAAG	0.658	82.01	66.67
17537	AGGAGATCACAAAGGCTGG	0.471	64.93	50
17538	GATCACAAAGGCTGGCAAT	0.485	45.23	50
17539	TCTTGGTCTTATTGCGGCA	0.693	53.15	66.67
17541	GATGTACTTCTCCCTCTGG	0.345	50.09	41.67
17542	CTCTGGTCATTATCTAAAC	0.514	60.88	41.67
17544	CAGTGGAAAGTACATCAGA	0.479	41.1	41.67
17545	ATTCAACAGCCTGATCTTG	0.557	67.09	66.67
17547	GGTCTCCAGGAATCTTGAG	0.646	37.14	25
17549	GCTTTATCAAAGTTTTGCA	0.460	44.69	58.33
17550	GGATGACAAACGCCTCATA	0.449	23.47	41.67
17551	GCCTCATAATCTGCAAACA	0.385	25.36	33.33
17552	TGCAAACATCTGAGTGAAA	0.424	27.43	58.33
17553	AACTTCCCGGATCTGAATG	0.747	69.24	58.33
17554	TTCTTCTTCATCACACTGA	0.752	57.55	91.67
17555	ACTTTGAGATCCTTATTGC	0.662	84.8	75
17556	TCCTTATTGCTGCTGGGGT	0.649	48.65	58.33
17557	ACGCACTTCCAACTTTTCC	0.608	64.57	58.33
17558	CTTAAGAAGCTCGTAGGAA	0.598	58.18	58.33
17559	TTAAGAAGCTCGTAGGAAT	0.654	68.88	75
17560	GAAGCTCGTAGGAATGCAA	0.508	29.77	41.67
17561	AGCCAGCAATGTTCCCATT	0.436	20.77	41.67
17562	GCCAGCAATGTTCCCATTC	0.453	50.72	33.33
17563	GCAATGTTCCCATTCCTCT	0.536	42.45	58.33
17564	CAATGTTCCCATTCCTCTT	0.500	49.1	50
17565	GCCCGCAGCCTCTTCAGCT	0.549	24.01	16.67
17566	CCAATTTGTTGGGGAACTG	0.679	58.72	50
17567	CAATGAAGTGGTTGTCAAT	0.487	51.89	50
17568	AGCTTTGACCGGTCATCCA	0.677	42.36	50
17569	ATCTAAGAAATGCAGGAGA	0.513	57.55	75
17573	TTTGACAGGAAAGTCAAAT	0.537	70.77	83.33
17574	TATTGGCCCATAGACCCCA	0.667	53.42	66.67
17575	CCAGGAGGTGGGAGCGGCA	0.249	25.72	25
17576	TGTATATGTAGCTCTCAAG	0.742	77.97	83.33
17577	CATAGGAGTTGAAGCGCTG	0.499	74.46	41.67
17578	TGCAGTTTGGTCACAGGAG	0.805	64.93	50
17579	CAGGAGTGTCTTCACCATC	0.829	54.23	33.33
17580	ACTCAGCATTGTGCATGTG	0.696	54.14	50
17581	ATTGTGCATGTGGTAGAGG	0.683	73.38	58.33
17582	GCATTGCACTGCAGATCTG	0.563	43.79	33.33
17583	CTTGCTAGTCACCTCTTCA	0.686	51.53	50
17584	ATGTGAGGGCAAACCCAAA	0.734	55.31	58.33
17585	ATGTCCGAGAGCCATCCTC	0.559	76.35	58.33
17586	GGCTCCCTGGGATCAGCCT	0.574	6.65	16.67
17587	TGGGTCTTGAATGCCAGCC	0.758	56.74	58.33
17588	ATTTGAATGTTCTTCTCAA	0.547	71.4	58.33
17589	CATAATGCCGACAGCTCGT	0.541	49.1	50
17590	CGGTCTCCGTGTCCAGTCC	0.604	29.23	25
17592	AGAGTATTCTTCAGCTAGG	0.639	69.06	58.33
17593	AGTATTCTTCAGCTAGGTC	1.016	67.09	58.33
17594	ATTCTTCAGCTAGGTCAGC	0.701	67.09	58.33
17595	CTTCAGCTAGGTCAGCCCG	0.613	46.31	33.33
17596	TCAGCTAGGTCAGCCCGAA	0.490	45.5	50
17598	TAGGTCAGCCCGAAGCGGG	0.623	70.23	58.33
17599	GGTCAGCCCGAAGCGGGTG	0.600	33.36	25
17600	TCAGCCCGAAGCGGGTGCT	0.376	30.04	50
17601	GCCCGAAGCGGGTGCTCAG	0.517	40.29	16.67
17603	CGAAGCGGGTGCTCAGGCT	0.463	32.37	33.33
17604	GAAGCGGGTGCTCAGGCTG	0.371	61.24	33.33
17605	GTCATTCACCAGTGCTATG	0.809	45.95	41.67
17606	TCATTCACCAGTGCTATGA	0.775	58.18	91.67
17607	CATTCACCAGTGCTATGAG	0.771	63.04	41.67
17609	GAGGGACTGGATTACTTGG	0.614	39.3	25
17610	AGGGACTGGATTACTTGGT	0.873	37.95	58.33
17612	CTGGATTACTTGGTCGGTT	0.666	37.95	41.67
17613	GATTACTTGGTCGGTTTTG	0.562	71.67	66.67
17614	ATTACTTGGTCGGTTTTGG	0.846	94.96	75
17615	ACTTGGTCGGTTTTGGTTG	0.870	82.01	66.67
17616	TGGTCGGTTTTGGTTGCTG	0.600	71.22	50
17618	CGGTTTTGGTTGCTGGCTT	0.417	31.65	33.33
17619	GTTTTGGTTGCTGGCTTCC	0.730	71.67	58.33
17620	TGGTTGCTGGCTTCCAGTT	0.718	27.43	50

17622	TTTCGGCACTAATTACTGG	0.828	73.38	66.67
17623	CACTAATTACTGGCAGACA	0.517	38.31	50
17624	ACTAATTACTGGCAGACAG	0.525	75.72	66.67
17625	TAATTACTGGCAGACAGAC	0.910	73.74	75
17626	AATTACTGGCAGACAGACC	0.743	88.67	75
17627	TACTGGCAGACAGACCTGC	0.527	61.42	66.67
17630	CCTGCCCCTTTTCGTCGAT	0.320	10.79	16.67
17631	TGCCCCTTTTCGTCGATGT	0.645	35.7	50
17632	CCTTTTCGTCGATGTTTGG	0.594	43.79	50
17633	CGTCGATGTTTGGGTGATA	0.570	29.5	33.33
17634	ATGTTTGGGTGATAGATCT	0.731	55.31	75
17635	TTTGGGTGATAGATCTTTG	0.828	88.31	75
17636	GGGTGATAGATCTTTGTTT	0.501	33.9	50
17637	ATAGATCTTTGTTTTAAAT	0.655	47.03	50
17638	TTTTAAATGTGATCTTCGG	0.834	91.46	83.33
17639	TGATCTTCGGTGGTTTGAA	0.659	59.44	66.67
17640	TCGGTGGTTTGAATGGGTA	0.718	42	58.33
17642	TTGAATGGGTACTCTGCTG	0.900	77.88	75
17648	TCTGAAGGCTCCCTTATCA	1.099	52.79	75
17649	CTCCCTTATCATATGGAGG	0.812	60.52	33.33
17650	ATATGGAGGGTTGTCAGGA	0.697	75.18	66.67
17652	GGAACAATAAGCCCTTGCC	0.794	63.76	41.67
17655	TAAGCCCTTGCCAAGTCAA	0.619	44.51	58.33
17656	AAGCCCTTGCCAAGTCAAT	0.587	48.65	50
17657	AGCCCTTGCCAAGTCAATA	0.753	42	41.67
17660	GCCAAGTCAATAAATTAGC	0.462	50.18	50
17662	AATTAGCTTCATCAACCTG	0.967	73.74	66.67
17663	TTAGCTTCATCAACCTGGA	1.115	59.44	66.67
17667	ATCAACCTGGATGTTACGG	0.614	69.24	58.33
17670	TGAGCTGTCCCCGGTACCC	0.719	65.29	50
17671	GAGCTGTCCCCGGTACCCT	0.772	34.17	33.33
17672	GCTGTCCCCGGTACCCTCT	0.650	10.79	33.33
17673	CCCCGGTACCCTCTTCCTG	0.747	43.79	16.67
17674	TTCCTGTTCTTTCAGCGCC	0.985	71.22	58.33
17675	TTCTTTCAGCGCCTCCTCC	0.919	65.47	66.67
17676	CAGCGCCTCCTCCGTGGCG	0.668	35.52	16.67
17677	AGCGCCTCCTCCGTGGCGT	0.719	38.22	33.33
17678	CGTATTTCTGGATGTACTC	0.776	58.72	50
17679	GGATGTACTCTTTAATTTT	0.705	35.16	41.67
17680	TGTACTCTTTAATTTTCTG	0.786	63.31	50
17681	TAATTTTCTGCTTGTATTC	0.931	84.89	83.33
17682	TTTTCTGCTTGTATTCTTC	0.951	94.96	83.33
17683	TTCTGCTTGTATTCTTCTG	0.777	77.88	66.67
17684	TCTGCTTGTATTCTTCTGG	1.039	77.61	75
17685	CTTGTATTCTTCTGGTCGG	0.864	65.02	50
17686	GTATTCTTCTGGTCGGTGG	0.825	71.67	58.33
17687	TCTGGTCGGTGGAGGTACA	0.957	27.79	41.67
17688	CGGTGGAGGTACATGGCTG	0.614	53.24	25
17689	GGTACATGGCTGCAGCGTC	0.553	54.95	33.33
17690	ACATGGCTGCAGCGTCACC	0.910	57.01	41.67
17691	ATGGCTGCAGCGTCACCAT	0.866	44.87	41.67
17693	TGAGAGGATCTATGGGGTT	0.494	46.49	66.67
17694	GAGGATCTATGGGGTTAGG	0.669	39.3	25
17695	GATCTATGGGGTTAGGATA	0.502	42.45	50
17696	GGGGTTAGGATAGGCCAAT	0.362	23.74	33.33
17697	AGGATAGGCCAATAACTGA	0.666	42.36	66.67
17698	AGGCAGGAAGGACTCAAAT	0.482	42	50
17699	GGAAGGACTCAAATATATT	0.416	45.23	41.67
17700	GAAGGACTCAAATATATTG	0.842	52.34	50
17701	GGACTCAAATATATTGGTA	0.486	38.58	41.67
17702	CAAATATATTGGTAAGATC	0.783	67.9	50
17703	AAATATATTGGTAAGATCA	0.684	65.11	75
17704	TATATTGGTAAGATCATAG	1.100	93.44	91.67
17705	TAAGATCATAGAGAGCTGT	0.958	46.76	83.33
17706	TCATAGAGAGCTGTCCAAG	0.810	84.8	75
17707	CATAGAGAGCTGTCCAAGT	0.860	49.1	50
17708	GTTTGATTAATTACATCTA	0.465	47.57	50
17709	TGATTAATTACATCTAGAC	1.058	76.98	75
17710	ACATCTAGACACACAGTTC	0.984	78.51	66.67
17711	ATCTAGACACACAGTTCCT	1.001	58.09	66.67
17712	AGACACACAGTTCCTGACG	0.999	71.85	50
17715	ATGGAAAATTTTATTCATG	0.640	76.53	50
17716	TTTTATTCATGAATCCTAT	1.064	68.62	83.33
17717	TTTATTCATGAATCCTATA	0.679	47.39	66.67
17718	ATTCATGAATCCTATAGAT	0.953	55.67	58.33
17719	ATCCTATAGATGGAGATTT	0.964	50.9	75
17720	TGGAGATTTGAAAGGGTAT	0.992	42.36	66.67
17722	ATTTGAAAGGGTATTTATC	1.016	93.97	58.33
17723	ATTTATCAGGTAGGTCCAC	0.779	73.74	66.67
17724	TTTATCAGGTAGGTCCACT	1.084	75.18	83.33
17726	GGTAGGTCCACTCTAACTT	0.642	44.69	50
17727	GTAGGTCCACTCTAACTTT	0.500	27.52	41.67

17728	ACTCTAACTTTCCATACTC	0.836	71.85	58.33
17729	ATACTCCGCCTTCATATGG	1.016	67.09	66.67
17730	TCCGCCTTCATATGGTGTT	0.678	37.95	58.33
17731	CTTCATATGGTGTTCCTTG	0.995	74.1	58.33
17732	GGTGTTCCTTGTGGTCCAT	0.340	14.57	25
17733	TGTTCCTTGTGGTCCATAA	0.917	53.15	75
17734	GTGGTCCATAAAACTTCAC	0.638	41.55	41.67
17735	TGGTCCATAAAACTTCACT	0.955	53.69	66.67
17736	TCCATAAAACTTCACTACA	0.756	45.14	66.67
17738	AAAACTTCACTACAAATTC	0.967	84.89	66.67
17740	CCTCCCAGGATCGTAACCT	0.841	37.05	33.33
17741	CTCCCAGGATCGTAACCTC	0.573	50.45	25
17742	CCCAGGATCGTAACCTCAT	0.777	28.15	25
17745	AACCTCATGTTTACTCTCG	0.980	74.01	66.67
17746	CCTCATGTTTACTCTCGAT	0.711	29.5	41.67
17747	CTCATGTTTACTCTCGATG	0.702	69.42	58.33
17748	TCATGTTTACTCTCGATGA	0.715	53.15	75
17750	TTTGGCAGTAAATCGTGTA	0.929	64.48	75
17751	GGCAGTAAATCGTGTAGGC	0.678	50.72	25
17752	TAAATCGTGTAGGCCTCTG	0.838	88.67	83.33
17753	GTAGGCCTCTGCTTGAGCT	0.895	30.04	25
17754	CTGCTTGAGCTGGGTCTTG	0.758	50.45	41.67
17755	TGGGTCTTGGATATTTGGT	0.839	42	58.33
17756	GGGTCTTGGATATTTGGTT	0.489	33.9	41.67
17757	TATTTGGTTCATTTAGAAG	0.888	91.19	75
17758	CATTTAGAAGTTCCTGTAT	0.521	49.1	66.67
17759	AGTTCCTGTATTCCTAATA	0.719	55.4	66.67
17760	TGTATTCCTAATAGGATCT	0.904	40.47	83.33
17761	GTATTCCTAATAGGATCTG	0.744	58.99	58.33
17763	TAGGATCTGTTTGATTGTG	0.874	56.29	58.33
17764	CTGTTTGATTGTGATGGCT	0.601	38.31	50
17765	GTTTGATTGTGATGGCTGG	0.560	71.67	58.33
17768	TGGCCTCCAGTCCTTGTCC	0.859	45.86	41.67
17769	TCCAGTCCTTGTCCTCCTC	0.705	46.22	41.67
17771	TCTAAGATGGACAGGCACA	0.760	55.94	75
17772	AAGATGGACAGGCACACTG	0.801	80.13	66.67
17773	AGATGGACAGGCACACTGT	0.592	55.94	58.33
17774	GGACAGGCACACTGTCCCC	0.478	48.29	33.33
17775	GGCACACTGTCCCCGAAGG	0.511	29.23	16.67
17776	TCCCCGAAGGGTACACATT	0.596	38.49	41.67
17777	CCCGAAGGGTACACATTCG	0.451	37.5	33.33
17778	GGGTACACATTCGGGTGAA	0.496	28.15	33.33
17779	TACACATTCGGGTGAAATA	0.789	55.31	66.67
17780	ATTCGGGTGAAATAATGGT	0.883	61.69	66.67
17782	GAAATAATGGTGGTTCGAA	0.591	58.18	58.33
17783	AAATAATGGTGGTTCGAAT	0.810	72.39	75
17784	AATGGTGGTTCGAATTTAC	0.892	82.01	66.67
17785	TGGTGGTTCGAATTTACAT	0.750	48.65	58.33
17786	GTGGTTCGAATTTACATTT	0.539	18.97	50
17787	TTCGAATTTACATTTTGGT	0.864	53.42	66.67
17788	TGGCGAAGATGGATAATCA	0.590	44.78	58.33
17789	CGAAGATGGATAATCATCT	0.527	44.69	58.33
17790	ATGGATAATCATCTTTGAA	0.652	53.96	50
17792	CATCTTTGAAAAGCATCCG	0.873	67.27	58.33
17795	AAAAGCATCCGTAGTTTAA	0.502	68.88	75
17796	AGCATCCGTAGTTTAAACA	0.681	35.97	58.33
17798	ATCCGTAGTTTAAACAAGC	0.922	74.01	50
17799	CGTAGTTTAAACAAGCCTC	0.732	60.97	50
17800	GTAGTTTAAACAAGCCTCC	0.825	67.27	66.67
17801	TAAACAAGCCTCCTTCCCA	0.780	75.18	75
17803	CAAGCCTCCTTCCCACGGA	0.524	38.67	33.33
17805	TCCTTCCCACGGAGTCCCT	0.721	23.65	50
17807	TTCCCACGGAGTCCCTTTC	0.893	54.77	58.33
17808	TCCCACGGAGTCCCTTTCT	0.631	31.92	58.33
17809	CCCACGGAGTCCCTTTCTT	0.456	27.88	25
17810	CACGGAGTCCCTTTCTTTC	0.531	60.52	33.33
17811	GAGTCCCTTTCTTTCCTGG	0.549	52.25	33.33
17812	TCCCTTTCTTTCCTGGAAT	0.839	42	58.33
17813	TTCCTGGAATGGCGCACTC	0.726	67.45	58.33
17815	GCACTCCCAGTTCATGAGG	0.722	33.36	25
17816	CACTCCCAGTTCATGAGGT	0.588	29.68	25
17817	ACTCCCAGTTCATGAGGTT	0.345	49.28	50
17818	CAGTTCATGAGGTTCATCG	0.782	72.21	66.67
17820	TTCATGAGGTTCATCGTGC	0.844	86.96	83.33
17821	AGGTTCATCGTGCCATCGG	0.818	63.94	50
17822	GGTTCATCGTGCCATCGGG	0.550	54.95	33.33
17823	GTTCATCGTGCCATCGGGA	0.488	30.04	33.33
17824	GACAGCCACGAAACCAAAT	0.516	23.38	33.33
17825	GCCACGAAACCAAATGGGT	0.569	28.15	25
17826	CCACGAAACCAAATGGGTG	0.854	61.15	25
17827	GAAACCAAATGGGTGGTCT	0.798	58.18	58.33
17828	TTCTTATCGCGCTCGCCAG	0.824	74.1	66.67

17829	TTATCGCGCTCGCCAGCAT	0.713	47.39	58.33
17830	GCCAGCATGCTTCTTGGCC	0.572	53.24	25
17831	GCCATGGGACCCTCAGCCC	0.457	44.15	33.33
17832	CCATGGGACCCTCAGCCCC	0.428	54.95	33.33
17833	ATGGGACCCTCAGCCCCTC	0.450	52.52	33.33
17834	GGCCCCAGGGTCGGTGGAG	0.440	40.29	16.67
17835	AGGGTCGGTGGAGGAAGCT	0.494	31.92	41.67
17836	GTGGAGGAAGCTTCAGTGC	0.585	50.45	41.67
17837	AAGCTTCAGTGCCACTGGC	0.579	52.52	41.67
17838	GTGCCACTGGCCAGGGCCC	0.430	35.52	16.67
17839	ACTGGCCAGGGCCCGACCG	0.415	50.36	33.33
17840	GGCCAGGGCCCGACCGGCT	0.430	14.93	25
17841	GCCCGACCGGCTTCGGCCC	0.364	22.57	8.33
17842	CCCGACCGGCTTCGGCCCT	0.491	0	16.67
17843	TTCGGCCCTGCCGCTGGGC	0.420	52.52	41.67
17844	CGTGGATCTCTGTGAGCAG	0.548	48.29	25
17845	ATCTCTGTGAGCAGACGGG	0.701	76.35	58.33
17846	GTGAGCAGACGGGCCCGAG	0.550	59.89	33.33
17847	ACGGGCCCGAGCTGCATAC	0.470	41.82	41.67
17848	CGGGCCCGAGCTGCATACT	0.498	0	8.33
17849	GGGCCCGAGCTGCATACTC	0.534	37.5	16.67
17850	CCCGAGCTGCATACTCCTC	0.567	22.57	16.67
17851	CTGCATACTCCTCGTAGTT	0.400	34.44	41.67
17852	ATACTCCTCGTAGTTCTCC	0.563	73.38	66.67
17853	TCGTAGTTCTCCAAGAGCA	0.778	42.36	66.67
17854	GTAGTTCTCCAAGAGCAGG	0.707	50.09	33.33
17855	TAGTTCTCCAAGAGCAGGC	0.760	80.13	75
17856	TCTCCAAGAGCAGGCGGCC	0.694	68.08	50
17857	AGGCGGCCCGCCTCCTCGT	0.443	17	25
17858	CCGCCTCCTCGTTGAGTGC	0.485	22.57	16.67
17859	CCTCGTTGAGTGCAGACTC	0.715	48.29	25
17860	CTCGTTGAGTGCAGACTCG	0.706	50.45	41.67
17861	GAGTGCAGACTCGGGGTTA	0.395	37.32	41.67
17862	GACTCGGGGTTAGGGTGGA	0.394	34.53	33.33
17863	GGGTGGATCAGCAGGCACT	0.557	26.62	33.33
17864	GTGGATCAGCAGGCACTTG	0.635	35.52	33.33
17865	GATGGTCAGCAGTACGTGT	0.593	27.52	33.33
17866	ATGGTCAGCAGTACGTGTC	0.855	76.26	58.33
17867	GGATGCCCAGCTCAGCCGT	0.523	17.45	25
17868	CCTCTTGAGCACGTTGACG	0.631	54.59	33.33
17869	TCTTGAGCACGTTGACGCA	0.745	53.15	58.33
17870	CTTGAGCACGTTGACGCAG	0.678	46.31	33.33
17871	GTTGACGCAGATCTCGCCA	0.856	44.96	41.67
17872	TCTCGCCATTGGCGCCCAC	0.544	50.36	41.67
17873	CATTGGCGCCCACGTTCGG	0.449	52.97	33.33
17874	CCACGTTCGGGTGGAAGAT	0.316	25.72	25
17875	CGTTCGGGTGGAAGATCTT	0.604	36.15	33.33
17876	TCTTGGTCAGGAAGTAGCC	0.917	75.72	58.33
17877	AGGAAGTAGCCCTTGGGTG	0.815	67.45	50
17878	GGAAGTAGCCCTTGGGTGG	0.547	57.73	41.67
17879	AGTAGCCCTTGGGTGGGGA	0.489	40.74	41.67
17880	AGCCCTTGGGTGGGGAGGC	0.463	54.5	33.33
17881	GGGGAGGCAGGGAAGTCCT	0.552	14.93	25
17882	CAGGGAAGTCCTTCCCCAG	0.404	53.24	25
17883	AGGGAAGTCCTTCCCCAGC	0.539	54.5	41.67
17884	TTCCCCAGCAGGAGTTTCA	0.540	53.69	66.67
17885	GAGTTTCATGCGGAACAGA	0.423	23.38	41.67
17886	CATGCGGAACAGACCTCCA	0.585	38.67	33.33
17887	GACCTCCAGCATATGGGGT	0.499	19.24	25
17888	CCAGCATATGGGGTCCCCT	0.365	32.01	25
17889	CATATGGGGTCCCCTCAGG	0.252	67.9	50
17890	ATATGGGGTCCCCTCAGGG	0.465	91.19	58.33
17891	GGGTCCCCTCAGGGCCCTC	0.446	29.23	16.67
17892	TCAGGGCCCTCGATGGTGA	0.359	34.08	50
17893	CAGGGCCCTCGATGGTGAC	0.451	35.52	16.67
17894	TGAGGTCCTCCTCGTTGGG	0.540	50.36	41.67
17895	GAGGTCCTCCTCGTTGGGA	0.357	19.24	25
17896	AGGTCCTCCTCGTTGGGAA	0.416	44.87	41.67
17897	GTCCTCCTCGTTGGGAAAG	0.339	35.52	25
17898	CCTCGTTGGGAAAGACCTT	0.530	29.5	25
17899	TGGGAAAGACCTTGATGCC	0.573	61.06	50
17900	GGAAAGACCTTGATGCCAT	0.565	45.23	50
17901	GACCTTGATGCCATCGGGT	0.664	34.17	33.33
17902	CCTTGATGCCATCGGGTGG	0.472	54.95	41.67
17903	TGATGCCATCGGGTGGGTC	0.617	63.31	50
17904	GCCATCGGGTGGGTCTGCG	0.515	50.45	33.33
17905	GGGTGGGTCTGCGGTCAGT	0.436	21.58	25
17906	TGCGGTCAGTGTCGTCACC	0.887	39.57	33.33
17907	TCCGAATCGGAGGGTGAAC	0.611	54.5	50
17908	CCGAATCGGAGGGTGAACT	0.553	15.2	33.33
17909	CGGAGGGTGAACTCTTCGG	0.626	46.4	33.33
17910	GAGGGTGAACTCTTCGGCA	0.599	34.17	25

17911	GGGTGAACTCTTCGGCATT	0.516	28.15	25
17913	CTTCGGCATTCTTTCTGAA	0.577	33.81	33.33
17914	ATTCTTTCTGAACAGCTCC	1.077	82.01	75
17915	AACAGCTCCGGATTCTGTG	0.978	84.17	50
17916	ACAGCTCCGGATTCTGTGT	0.854	31.56	41.67
17917	AGCTCCGGATTCTGTGTCA	0.548	42.36	50
17918	GCTCCGGATTCTGTGTCAG	0.796	54.59	25
17919	GTCAGCAGGTCAGCGAGGT	0.586	37.32	33.33
17920	GCAGGTCAGCGAGGTCCAT	0.438	10.79	16.67
17921	AGGTCAGCGAGGTCCATCC	0.805	63.4	58.33
17922	GGTCAGCGAGGTCCATCCG	0.716	33.36	25
17924	AGCGAGGTCCATCCGCAGG	0.818	54.5	41.67
17925	GCGAGGTCCATCCGCAGGG	0.597	46.4	33.33
17926	GGTCCATCCGCAGGGGCTC	0.559	48.29	25
17927	CCATCCGCAGGGGCTCCCT	0.601	32.37	33.33
17928	GGGGCTCCCTGATATTCGG	0.500	22.57	8.33
17929	TGATATTCGGTCTATTCAC	0.899	75.72	75
17930	CGGTCTATTCACCAGCACA	0.590	28.15	25
17931	CTATTCACCAGCACATTGA	0.691	54.14	75
17932	GCACATTGAGGGCCTCCAG	0.482	48.29	33.33
17933	ACATTGAGGGCCTCCAGGA	0.742	52.16	58.33
17935	CCTCCAGGACTTGGCAAGT	0.318	25.72	25
17936	TGGCAAGTCTTGGTGCAAG	0.896	64.57	50
17937	TCTTGGTGCAAGGCTTCCA	0.777	55.4	66.67
17938	TGGTGCAAGGCTTCCAGTT	0.778	45.14	50
17939	CTTCCAGTTCTCACTGCTG	0.749	65.02	33.33
17940	TCCAGTTCTCACTGCTGAT	0.829	42.36	58.33
17941	CCAGTTCTCACTGCTGATG	0.657	39.39	33.33
17942	CAGTTCTCACTGCTGATGA	0.613	44.6	58.33
17943	TCTCACTGCTGATGATGGG	0.740	69.06	58.33
17944	GATGATGGGCAGGCAAATC	0.713	65.02	41.67
17946	CTCGTCCACGTTGGGGTGG	0.676	35.52	33.33
17947	TCGTCCACGTTGGGGTGGT	0.649	41.37	50
17948	CGTCCACGTTGGGGTGGTA	0.374	10.79	16.67
17949	TCCACGTTGGGGTGGTAGA	0.530	38.58	50
17950	TGGGGTGGTAGATCTTGGT	0.908	37.95	50
17951	GGGGTGGTAGATCTTGGTT	0.436	25	25
17952	GTGGTAGATCTTGGTTGTG	0.458	54.23	41.67
17953	GTAGATCTTGGTTGTGAAT	0.510	27.52	41.67
17954	GATCTTGGTTGTGAATTTG	0.859	65.02	58.33
17955	TTGTGAATTTGATCATGGG	0.952	80.67	66.67
17957	TTGATCATGGGAGGCTTGA	0.867	58.09	83.33
17958	TGATCATGGGAGGCTTGAA	0.712	53.15	66.67
17959	GGAGGCTTGAACGGATACT	0.537	31.74	33.33
17960	AGGCTTGAACGGATACTCC	0.846	64.57	50
17961	ACGGATACTCCGGCGGGAA	0.430	34.71	41.67
17962	CGGATACTCCGGCGGGAAG	0.381	29.23	25
17963	ACTCCGGCGGGAAGCTGAT	0.523	42.72	41.67
17964	GGGAAGCTGATGCGCAGGT	0.709	8.9	33.33
17965	GGAAGCTGATGCGCAGGTT	0.516	32.37	33.33
17966	TGCGCAGGTTGAAGGCTTT	0.698	35.7	50
17968	GTAGGGAGGTTGGTCGGGT	0.640	47.75	33.33
17969	GGGAGGTTGGTCGGGTAGG	0.556	44.15	25
17970	GAGGTTGGTCGGGTAGGAG	0.635	56.74	33.33
17971	GGTTGGTCGGGTAGGAGGA	0.495	32.37	33.33
17972	TTGGTCGGGTAGGAGGAGA	0.799	44.87	58.33
17973	GTCGGGTAGGAGGAGAGCG	0.737	50.45	25
17974	GGTAGGAGGAGAGCGTGCC	0.625	59.98	41.67
17975	TAGGAGGAGAGCGTGCCAC	0.969	75.99	66.67
17976	AGGAGAGCGTGCCACACCA	0.895	38.58	41.67
17977	GAGCGTGCCACACCAGGAC	0.742	52.7	33.33
17979	CAGGACATTGGCATCATCG	0.971	63.31	41.67
17980	AGGACATTGGCATCATCGC	1.072	64.93	41.67
17981	GGACATTGGCATCATCGCT	0.873	29.5	33.33
17982	GGCATCATCGCTGGACAGG	0.776	46.94	33.33
17983	CGCTGGACAGGTTCCGCAG	0.761	46.94	25
17984	CTGGACAGGTTCCGCAGGT	0.738	30.67	33.33
17985	ACAGGTTCCGCAGGTATGG	0.883	65.29	50
17986	ATTTCTCTTTGTGTTGGAT	0.691	57.46	58.33
17987	TTCTCTTTGTGTTGGATGA	0.930	55.31	75
17989	CTCTTTGTGTTGGATGACG	0.863	60.88	50
17990	TTGTGTTGGATGACGCCCT	1.071	57.55	66.67
17991	CCCTTCTGAATCAGATCAG	0.732	50.18	50
17992	TGAATCAGATCAGGGATTT	0.810	55.94	83.33
17993	TCAGGGATTTCCACTGCCA	0.714	49.28	50
17994	AGGGATTTCCACTGCCAGC	0.485	54.5	41.67
17996	ACTGCCAGCCATGGACCCA	0.352	45.5	41.67
17997	CTGCCAGCCATGGACCCAG	0.605	52.7	33.33
17998	TGGACCCAGCCCCAGAGCC	0.699	46.22	41.67
18000	AGCCCCAGAGCCATGAGAT	0.538	41.01	33.33
18001	AGCCATGAGATGAGCTAGT	0.538	37.95	58.33
18002	CCATGAGATGAGCTAGTCC	0.617	65.02	41.67

18003	TCCAAATTTGGGCACATTC	0.679	64.93	75
18004	ATTTGGGCACATTCCTGGC	0.772	88.67	58.33
18005	ATTCCTGGCCCACAAAGGT	0.569	59.44	50
18007	ACAAAGGTTTGAAATGATC	0.988	75.72	66.67
18008	AAGGTTTGAAATGATCCGT	1.060	50.9	66.67
18009	AGGTTTGAAATGATCCGTC	1.227	73.47	66.67
18010	GTTTGAAATGATCCGTCAG	0.657	74.46	50
18011	TTGAAATGATCCGTCAGGC	1.173	84.17	75
18012	CCGTCAGGCATATTTTGCC	0.999	56.47	33.33
18013	TGCCACCCCTGTACATCTT	0.623	20.77	41.67
18014	CACCCCTGTACATCTTTGC	1.081	56.47	41.67
18015	CCTGTACATCTTTGCTGTC	0.501	37.14	33.33
18016	TGTACATCTTTGCTGTCTT	1.126	59.44	66.67
18017	ATCTTTGCTGTCTTTCCAT	0.699	61.6	66.67
18018	CTTTGCTGTCTTTCCATCC	1.026	71.67	58.33
18019	TCTTTCCATCCAGCTCAGG	1.201	60.79	58.33
18020	CATCCAGCTCAGGAACTGC	0.801	61.24	41.67
18021	CCAGCTCAGGAACTGCAAT	0.616	20.86	16.67
18022	GAACTGCAATTTCTGGGGC	0.617	56.38	33.33
18023	AACTGCAATTTCTGGGGCA	0.449	58.09	58.33
18024	TGCAATTTCTGGGGCAGTA	0.522	42.36	66.67
18026	TGGGGCAGTAGTAGGATAT	0.782	40.74	66.67
18027	CAGTAGTAGGATATGTGAT	0.692	44.6	50
18028	ATATGTGATAGGAATGTCA	1.146	68.35	83.33
18029	GATAGGAATGTCAAACTCG	0.957	74.46	58.33
18030	TCAAACTCGATGTCAAACT	1.216	55.4	83.33
18031	GATGTCAAACTCATATTTC	0.669	74.1	58.33
18032	ATGTCAAACTCATATTTCA	0.753	54.32	58.33
18033	AACTCATATTTCAGGAGGT	0.435	55.31	58.33
18034	CAGGAGGTCATGGATATAC	0.960	56.47	58.33
18035	GGAGGTCATGGATATACCA	0.314	14.57	25
18037	ATATACCAGCATTTTCCAA	0.568	57.46	66.67
18038	TTTCCAAACCACCGAGTTC	0.977	84.8	75
18039	CCAAACCACCGAGTTCCTT	0.322	27.52	33.33
18040	AACCACCGAGTTCCTTCCT	0.618	33.72	41.67
18041	GTTCCTTCCTTGTTGGACT	0.835	48.74	41.67
18042	CTTCCTTGTTGGACTCCAG	0.663	65.02	33.33
18043	TGGACTCCAGTCGGAACCA	0.719	23.65	41.67
18044	TCGGAACCAATCGTTGTCA	0.761	29.32	66.67
18045	GGAACCAATCGTTGTCAGC	0.780	61.51	33.33
18046	CGTTGTCAGCATTCTTGTT	0.433	21.22	33.33
18047	TTGTCAGCATTCTTGTTGT	0.864	61.6	75
18048	CATTCTTGTTGTTCTCCAC	0.689	71.67	50
18049	GTTGTTCTCCACATACCGG	0.723	56.38	33.33
18051	TCTCCACATACCGGATAAG	0.784	56.38	58.33
18052	ACATACCGGATAAGGGACT	0.746	40.47	66.67
18053	CATACCGGATAAGGGACTG	0.788	71.67	41.67
18054	ATACCGGATAAGGGACTGA	0.889	61.69	75
18055	TACCGGATAAGGGACTGAT	0.991	53.69	66.67
18056	CTGATATTCCTCCTTCAGT	0.752	44.6	50
18057	ATATTCCTCCTTCAGTCGC	1.066	73.74	66.67
18058	TATTCCTCCTTCAGTCGCT	0.931	66.1	75
18059	ATTCCTCCTTCAGTCGCTG	1.005	73.38	41.67
18060	TCCTTCAGTCGCTGCACCC	0.859	63.94	58.33
18062	AGTCGCTGCACCCACAACT	0.804	44.96	50
18064	TCACGATCTCGGGGTCCGG	0.566	65.29	50
18065	TTTCTTTTCTATGCCTACT	1.016	59.44	75
18066	CTTTTCTATGCCTACTAGC	1.060	71.67	58.33
18067	TACTAGCTGACTGGCCTTC	0.919	65.2	75
18068	CCTTCCTTTTCTGTGTTGA	0.655	42.45	50
18069	TGTTGAGTTGTGTACTCTG	0.830	75.72	66.67
18070	TTGTGTACTCTGGAGTCAC	1.080	80.67	66.67
18071	GTGTACTCTGGAGTCACCA	0.803	44.6	41.67
18072	GTCACCAGCCTCTGGTAGA	0.635	37.32	33.33
18073	TCACCAGCCTCTGGTAGAT	0.970	46.49	50
18074	CTCTGGTAGATTATCAAGC	1.010	69.42	50
18075	AGATTATCAAGCATCTCTT	0.994	61.69	75
18076	GATTATCAAGCATCTCTTC	0.859	56.74	58.33
18077	TTATCAAGCATCTCTTCCT	1.130	71.13	83.33
18078	ATCAAGCATCTCTTCCTCA	0.916	46.67	66.67
18079	CAAGCATCTCTTCCTCATC	0.951	65.02	41.67
18080	GCATCTCTTCCTCATCAGC	0.738	43.79	25
18081	TTTCTGTCTTGCATGCTTC	0.971	88.31	83.33
18082	CTGTCCACTGTCTGGCATT	0.775	41.1	33.33
18083	GTCCACTGTCTGGCATTCT	0.950	31.65	41.67
18084	TCTGGCATTCTTGAGGAAG	0.760	71.85	58.33
18086	ATTATATTTAAATTCTGAG	0.859	93.44	75
18087	TTAAATTCTGAGGATATGT	1.140	62.32	83.33
18088	AGCCATGAGCGGGTCATCA	0.756	38.22	50
18089	CCATGAGCGGGTCATCAGG	0.835	54.95	33.33
18090	CATGAGCGGGTCATCAGGG	0.912	52.61	33.33
18091	ATGAGCGGGTCATCAGGGT	1.007	51.53	50

18092	GGGTCATCAGGGTTGGGTT	0.527	27.88	25
18093	TGGGTTCTGACATGAGCAG	1.045	45.59	50
18094	GGTTCTGACATGAGCAGCT	0.794	38.4	41.67
18095	GCAGCTGAATAGAGGTCAA	0.492	29.5	33.33
18096	TAGAGGTCAACACAGTTGC	1.145	80.13	83.33
18097	GGTCAACACAGTTGCGATG	0.848	39.39	33.33
18098	AACACAGTTGCGATGTTGA	1.026	61.6	66.67
18099	ACAGTTGCGATGTTGAGGG	1.062	77.61	58.33
18100	GGGATGGTCTCCAAGCACC	0.804	44.15	33.33
18101	GGATGGTCTCCAAGCACCT	0.656	32.37	33.33
18102	GATGGTCTCCAAGCACCTT	0.605	27.52	25
18103	GTCTCCAAGCACCTTTTGG	0.849	69.96	41.67
18104	AGCACCTTTTGGTGGCAAT	0.438	42.36	50
18105	CTTTTGGTGGCAATTTGAG	0.685	77.97	58.33
18106	TGGTGGCAATTTGAGAACA	0.533	27.43	50
18107	GGCAATTTGAGAACATCCA	0.719	27.61	50
18108	CAATTTGAGAACATCCAGA	0.808	57.64	66.67
18109	GAGAACATCCAGACAAATC	0.750	63.67	50
18110	AACATCCAGACAAATCCTT	0.799	42.63	66.67
18111	CATCCAGACAAATCCTTCC	0.961	67.27	58.33
18112	GACAAATCCTTCCAGCAGA	0.878	38.31	50
18113	AATCCTTCCAGCAGAATCA	0.954	61.69	75
18114	AATCAATGTTTGGATGATA	0.674	55.67	58.33
18115	TGTTTGGATGATAAATTGG	1.013	75.72	83.33
18116	TTTGGATGATAAATTGGAG	1.071	88.31	66.67
18117	GGATGATAAATTGGAGTGA	0.568	38.4	58.33
18118	AAATTGGAGTGAGAAATCG	1.068	88.67	83.33
18119	ATTGGAGTGAGAAATCGGA	1.093	61.69	66.67
18120	AAATCGGATCTGAGGAGGT	1.004	66.1	66.67
18121	TCGGATCTGAGGAGGTTCA	1.051	23.02	58.33
18122	CGGATCTGAGGAGGTTCAA	0.565	25.36	33.33
18124	GAGGTTCAAATGGGTACCT	0.631	18.97	41.67
18125	TTCAAATGGGTACCTCTCA	0.874	55.31	83.33
18126	CAAATGGGTACCTCTCAGG	0.785	73.92	58.33
18127	ATGGGTACCTCTCAGGAAT	1.141	51.44	50
18128	ACCTCTCAGGAATGATAAC	0.754	50	41.67
18129	CTCTCAGGAATGATAACTT	0.794	46.85	50
18130	ATGATAACTTCTAGCTTAA	0.899	54.32	66.67
18131	AAAACACCTTTCTCATAAG	0.925	73.74	58.33
18132	CCTTTCTCATAAGGTGTGT	0.747	36.15	58.33
18133	GTGTTGGCTCCACCTAATA	0.574	38.31	50
18134	CTCCACCTAATATTTGAGC	0.871	47.84	41.67
18135	CACCTAATATTTGAGCTCG	0.900	57.01	50
18136	ACCTAATATTTGAGCTCGC	0.716	64.93	58.33
18137	TAATATTTGAGCTCGCAGG	1.021	90.92	91.67
18138	ATATTTGAGCTCGCAGGTC	1.008	88.67	75
18139	TCGCAGGTCATCCATTTGG	0.953	60.52	66.67
18140	GTCATCCATTTGGTCTTTA	0.632	29.68	50
18141	GGTCTTTATCTTGCCAACA	0.758	29.5	41.67
18142	CTTGCCAACATGTGATGCC	0.873	70.05	41.67
18143	TGCCAACATGTGATGCCTG	0.908	49.64	41.67
18144	ACCATTTTGTTTTCTTTGG	1.013	64.93	66.67
18145	TTTGTTTTCTTTGGATTCT	1.057	55.67	75
18146	TTTCTTTGGATTCTTGTTA	1.146	64.21	83.33
18147	TTCTTGTTACATGTTCGCA	1.039	61.6	75
18148	TCTTGTTACATGTTCGCAC	1.252	84.26	75
18149	TTGTTACATGTTCGCACAT	0.887	40.38	66.67
18150	AAAAGAATTATCCGGTGGT	0.967	71.13	75
18151	AAAGAATTATCCGGTGGTC	0.964	82.01	66.67
18152	TATCCGGTGGTCGTCTCTT	0.827	65.74	58.33
18153	TGGTCGTCTCTTTTCCTTG	1.098	71.22	66.67
18154	GGTCGTCTCTTTTCCTTGC	1.121	37.14	25
18155	TTTCCTTGCAGCTGGAAAG	1.030	84.26	75
18156	TCCTTGCAGCTGGAAAGCA	1.103	27.43	50
18157	AGCTGGAAAGCATGCTAAT	0.675	45.14	50
18158	GGAAAGCATGCTAATAATG	0.932	43.79	41.67
18159	AAAGCATGCTAATAATGCT	0.848	59.44	58.33
18160	AATGCTAAGACAAACTGAT	1.070	64.48	66.67
18161	ATGCTAAGACAAACTGATT	1.042	51.44	58.33
18162	CTAAGACAAACTGATTGGA	0.850	36.42	50
18163	AGACAAACTGATTGGACTG	1.215	71.85	58.33
18164	CTGATTGGACTGAGAGCGC	1.088	57.1	41.67
18165	GAGAGCGCTGGGGACCAGT	0.931	34.53	33.33
18166	GCGCTGGGGACCAGTCTTC	0.805	39.75	41.67
18167	CGCTGGGGACCAGTCTTCT	0.884	27.88	33.33
18168	GGGGACCAGTCTTCTGTTA	0.647	3.78	25
18169	GGACCAGTCTTCTGTTAGA	0.733	29.5	33.33
18170	AGTCTTCTGTTAGAATGGA	0.641	31.56	50
18171	TCTTCTGTTAGAATGGATA	0.839	61.69	75
18172	CTGTTAGAATGGATAAACA	0.662	44.6	50
18173	TTAGAATGGATAAACAGAT	0.966	57.91	75
18174	TAGAATGGATAAACAGATA	0.859	51.53	66.67

18175	TGACCATTGCTATAAACAT	0.962	46.49	58.33
18176	TTGCTATAAACATGAGGAT	1.043	50.9	75
18177	GCTATAAACATGAGGATGA	0.788	41.19	66.67
18178	TATAAACATGAGGATGAAC	1.054	73.74	75
18179	TAAACATGAGGATGAACAG	1.126	88.67	75
18180	AACATGAGGATGAACAGGA	1.083	60.34	75
18181	ACATGAGGATGAACAGGAA	0.730	53.15	58.33
18182	ATGAGGATGAACAGGAATA	1.040	60.34	66.67
18183	GGATGAACAGGAATATTTT	0.535	45.23	50
18184	TGAACAGGAATATTTTCAC	0.820	84.26	75
18185	AACAGGAATATTTTCACCA	1.151	66.64	66.67
18186	ACAGGAATATTTTCACCAG	1.078	71.85	50
18187	AGGAATATTTTCACCAGTA	0.944	45.14	58.33
18188	GAATATTTTCACCAGTAAA	0.912	45.32	50
18189	TTCACCAGTAAACATGACC	1.032	82.91	75
18190	GACCTGAGGAGAGTCAAAA	0.950	42.99	50
18191	CTGAGGAGAGTCAAAAGGA	0.784	41.1	41.67
18192	GAGAGTCAAAAGGATATCG	0.857	48.2	50
18193	AGAGTCAAAAGGATATCGA	0.747	57.82	66.67
18194	AGTCAAAAGGATATCGACT	0.814	55.58	58.33
18195	CAAAAGGATATCGACTACT	1.235	51.35	66.67
18197	TATCGACTACTAAATTTAA	0.799	40.74	58.33
18198	ATCGACTACTAAATTTAAA	0.880	51.17	50
18199	GACTACTAAATTTAAATAG	0.835	59.35	58.33
18200	CTAAATTTAAATAGAAGTT	0.961	47.57	58.33
18201	TTAAATAGAAGTTGAAATT	0.975	67.36	83.33
18202	TTCCCCTTCATATAAGGTA	1.111	50.9	66.67
18203	ATATAAGGTACCTGGTGCA	0.780	68.35	83.33
18204	TAAGGTACCTGGTGCACCT	0.932	62.23	58.33
18205	TACCTGGTGCACCTTCCAT	0.861	54.95	58.33
18206	CTGGTGCACCTTCCATGTC	0.949	35.52	25
18207	GGTGCACCTTCCATGTCTA	0.389	20.86	16.67
18208	CATGTCTACAATCCACTGT	1.104	44.69	66.67
18209	ATGTCTACAATCCACTGTG	1.152	82.91	66.67
18210	TGTCTACAATCCACTGTGT	0.993	31.56	66.67
18211	TCTACAATCCACTGTGTAA	0.926	55.94	75
18212	TACAATCCACTGTGTAATT	1.118	40.38	66.67
18213	CACTGTGTAATTGAATTTT	0.768	36.78	50
18214	GTGTAATTGAATTTTGAAC	0.736	65.65	50
18215	ATTGAATTTTGAACACTCT	1.091	55.67	66.67
18216	TTGAATTTTGAACACTCTT	1.135	51.53	66.67
18217	AATTTTGAACACTCTTCTC	1.156	88.67	75
18218	CTCTTCTCATTTAAGGTCA	0.750	38.31	58.33
18219	TCTTCTCATTTAAGGTCAT	0.689	38.22	58.33
18220	CTCATTTAAGGTCATTCCA	0.964	38.31	50
18221	TTTAAGGTCATTCCAGGAG	1.003	90.92	91.67
18222	TTAAGGTCATTCCAGGAGG	0.993	88.67	75
18223	AATATGTATTCGTTTGCCA	1.100	72.39	75
18224	TGTATTCGTTTGCCAGATA	0.916	38.22	66.67
18225	ATTCGTTTGCCAGATATAT	0.959	59.44	66.67
18226	ATTCGTCGGAATATAAGAG	0.988	75.63	58.33
18227	CTGCCAATGCCAAAGTCAG	0.764	57.01	25
18228	GGATGACAATCAGTACAGC	0.624	50.09	33.33
18229	CAGTACAGCTGACCCACCA	0.834	37.32	41.67
18231	GGTTGTCTCTTGGCTGGTG	0.558	40.02	25
18232	TTGGCTGGTGGCCCTGGAT	0.760	44.87	50
18234	ACATCTGGTGAAGTAGACA	0.731	59.44	58.33
18235	GAAGTAGACAACCTTCTCT	0.788	50.99	66.67
18236	ACTCTGCTGCTGGGCCCGG	0.474	50.36	41.67
18237	CTGCTGCTGGGCCCGGCGC	0.353	35.52	25
18238	TTCTTAGCTACAGGTTGGG	0.785	80.13	83.33
18240	AGGTTGGGTAGGTTGATGA	0.479	50.9	75
18241	TGGGTAGGTTGATGAAAGG	0.562	58.27	58.33
18246	TGGCCGTAGCACCCTGGAA	0.751	31.92	41.67
18247	GGTATATCATCTTGTAAAT	0.476	32.37	41.67
18248	TTGTAAATCAGTTAGTGAA	0.675	56.56	75
18249	AGTGGTTTAAGTCTGACTC	0.887	77.61	58.33
18252	TTCCAGATGAATTGACTTC	0.753	76.26	83.33
18253	TCCAGATGAATTGACTTCT	0.796	44.6	75
18254	CCAGATGAATTGACTTCTG	0.605	52.07	41.67
18255	CAGATGAATTGACTTCTGC	0.544	69.96	58.33
18256	CTGCCTTAAAGCTTATTTG	0.560	62.77	50
18257	GTTCTTTGGCTTCTTGGTC	0.567	71.31	50
18258	TCTTTGGCTTCTTGGTCAG	0.731	75.72	75
18260	GCTTCCAGATTTTAAAGGC	0.504	61.51	41.67
18261	TTCCAGATTTTAAAGGCAA	0.705	51.44	66.67
18262	TCCAGATTTTAAAGGCAAC	0.552	64.93	58.33
18267	GCAAGTGCTCTTCTTTCCT	0.707	42.45	41.67
18269	AACCTATGGCTCCCTCTCC	0.610	67.45	58.33
18270	TGGCTCCCTCTCCTTTTGT	0.417	27.07	50
18271	CCCTCTCCTTTTGTTGGCA	0.287	16.73	16.67
18276	TGATGGCTAATAATGCTGT	0.547	46.76	75

18277	TGGCTAATAATGCTGTCCT	0.748	47.03	66.67
18278	TAATGCTGTCCTTATACTC	0.914	88.67	75
18280	AGGATGATGGCCTGAGATG	0.647	67.72	50
18281	ATGATGGCCTGAGATGCTC	0.641	77.88	58.33
18282	TGGCCTGAGATGCTCAAAC	0.726	66.82	50
18283	CCTGAGATGCTCAAACAGA	0.560	32.28	41.67
18286	CCCACTTCAAATCGACCAT	0.563	27.61	41.67
18288	CACTTCAAATCGACCATTA	0.463	41.1	58.33
18290	AATCGACCATTAGCCGTTA	0.840	44.51	58.33
18293	TAATGCTTGGTGGTTTCAT	0.681	72.39	75
18295	CATGGGATACTCTGGTGGC	0.536	64.03	33.33
18296	ACTATCCGCCCGTGATAAA	0.518	38.22	58.33
18297	ACTCCTCCATCAAAATCGG	0.480	54.14	41.67
18300	GGCTGCGCATGGTAATGAT	0.552	25.36	25
18302	GAGCTGTCTGTTTGAACAT	0.646	31.65	41.67
18303	CTGTCTGTTTGAACATTTC	0.766	60.88	50
18304	TCTGTTTGAACATTTCGGG	0.927	77.61	75
18305	TTTGAACATTTCGGGATTT	0.738	44.51	75
18306	TTTCGGGATTTTGTTTGTA	0.661	65.74	66.67
18307	TTCGGGATTTTGTTTGTAC	0.739	80.31	66.67
18308	CGGGATTTTGTTTGTACTG	0.701	41.28	33.33
18309	TTGTTTGTACTGATTTGCT	0.778	61.6	75
18310	TTGTACTGATTTGCTACTA	0.879	55.31	75
18311	TGTACTGATTTGCTACTAC	0.921	82.01	75
18312	ACTGATTTGCTACTACAGC	0.874	75.36	58.33
18313	GATTTGCTACTACAGCATC	0.655	56.74	50
18314	TTTGCTACTACAGCATCCT	0.921	64.48	75
18315	TTGCTACTACAGCATCCTG	0.930	56.29	58.33
18316	TGCTACTACAGCATCCTGT	0.899	50.9	83.33
18317	CTACAGCATCCTGTGGATC	0.676	56.38	33.33
18318	AGCATCCTGTGGATCATCT	0.680	33.72	58.33
18319	CATCCTGTGGATCATCTGG	0.667	65.02	41.67
18320	ATCCTGTGGATCATCTGGC	0.749	79.77	58.33
18321	TGTGGATCATCTGGCTCTG	0.825	69.06	50
18322	ATCTGGCTCTGCAGCTGCC	0.818	59.17	41.67
18323	TGGCTCTGCAGCTGCCAAT	0.850	34.17	58.33
18324	CTCTGCAGCTGCCAATAGT	0.620	41.1	33.33
18325	TAGTGCTTGCAATGACAAT	0.854	55.31	66.67
18326	AGTGCTTGCAATGACAATA	0.892	48.74	58.33
18327	TGACAATAATACCGTGCGG	0.633	69.06	66.67
18328	ATACCGTGCGGAGAGTCAT	0.857	59.44	50
18329	TGCGGAGAGTCATTGCAGC	0.871	60.79	41.67
18330	GGAGAGTCATTGCAGCTGC	0.474	48.29	41.67
18331	GAGAGTCATTGCAGCTGCC	0.576	42.18	25
18332	GAGTCATTGCAGCTGCCCA	0.442	40.83	33.33
18333	ATTGCAGCTGCCCATTGAT	0.484	59.44	58.33
18334	GCTGCCCATTGATCTTTCA	0.573	14.57	33.33
18335	CTGCCCATTGATCTTTCAG	0.792	63.31	33.33
18336	TCTTTCAGGATATCCAAAC	0.871	80.76	83.33
18337	CTTTCAGGATATCCAAACA	0.587	49.1	50
18338	TTCAGGATATCCAAACAAA	0.789	58.09	66.67
18339	TATCCAAACAAATAGCCCC	0.812	87.05	75
18340	ATCCAAACAAATAGCCCCT	0.710	53.69	66.67
18341	AATAGCCCCTGTGACGGAA	0.555	51.17	50
18342	TGACGGAACTAATATTAGG	0.749	71.85	58.33
18343	ACGGAACTAATATTAGGAT	0.532	29.32	50
18344	AACTAATATTAGGATGCCA	0.616	55.31	66.67
18345	TATTAGGATGCCATATTTT	0.673	68.62	83.33
18346	TAGGATGCCATATTTTAGT	0.760	57.19	75
18347	CATATTTTAGTGATAAACC	0.812	74.19	50
18348	TATTTTAGTGATAAACCGG	0.897	91.46	83.33
18349	ATTTTAGTGATAAACCGGA	0.730	68.35	83.33
18350	ATTAAATGGGTATGTTTCT	0.723	62.32	75
18351	TAAATGGGTATGTTTCTGG	0.724	97.21	100
18352	ATGGGTATGTTTCTGGTAT	0.763	57.73	58.33
18353	TTTTATCTCTAGTTGGTAT	0.785	53.69	75
18354	TCTAGTTGGTATCTTCCTC	0.793	82.01	66.67
18355	TAGTTGGTATCTTCCTCCT	0.754	55.31	75
18356	AGTTGGTATCTTCCTCCTT	0.681	53.15	58.33
18357	TTGGTATCTTCCTCCTTCA	0.827	48.65	75
18358	CTTCCTCCTTCATATGGTG	0.695	50.09	33.33
18359	CTCCTTCATATGGTGTGTC	0.642	47.84	41.67
18360	TCCTTCATATGGTGTGTCT	0.810	45.14	75
18361	CCTTCATATGGTGTGTCTG	0.728	65.02	41.67
18362	TTCATATGGTGTGTCTGGA	0.739	61.6	75
18363	CATATGGTGTGTCTGGAGG	0.669	77.97	50
18364	TGGTGTGTCTGGAGGTCCT	0.746	38.58	50
18365	TGTCTGGAGGTCCTGCTAT	0.698	52.79	66.67
18366	GTCTGGAGGTCCTGCTATT	0.561	41.1	33.33
18367	TCTGGAGGTCCTGCTATTT	0.654	46.49	66.67
18368	AGGTCCTGCTATTTCTCCT	0.694	48.65	50
18369	GGTCCTGCTATTTCTCCTC	0.548	54.32	33.33

18370	CTATTTCTCCTCTTAATTC	0.742	63.04	58.33
18371	TCCTCTTAATTCTGTAAAA	0.476	38.58	50
18372	TGTAAAATTCTCATCTACA	0.895	58.45	66.67
18373	GTAAAATTCTCATCTACAA	0.551	45.32	50
18374	TAAAATTCTCATCTACAAG	0.913	91.19	75
18375	AAAATTCTCATCTACAAGA	0.844	49.64	66.67
18376	AAATTCTCATCTACAAGAT	0.813	62.32	66.67
18377	TTCTCATCTACAAGATCTA	0.857	57.55	75
18378	CTCATCTACAAGATCTACT	0.882	46.85	58.33
18379	TCATCTACAAGATCTACTT	0.889	58.18	75
18380	CTACAAGATCTACTTTAAT	0.458	44.96	41.67
18381	GGTCTTTGTCCCATATGCT	0.531	35.79	41.67
18382	GTCTTTGTCCCATATGCTG	0.517	60.88	50
18383	GTCCCATATGCTGGCCAAG	0.520	50.45	25
18384	GCCAAGTGTGTCCACCACC	0.522	44.15	33.33
18385	ACCACCTGCTCAGTCAGAG	0.468	67.45	41.67
18386	CTCAGTCAGAGCAGAGATG	0.427	48.2	33.33
18387	CGAGGAGACTGGACAGCCA	0.674	25.72	25
18388	TGATGGAGGGCTGGGTGGC	0.608	74.73	58.33
18389	TGGAGGGCTGGGTGGCTGA	0.512	38.58	58.33
18390	GGTGGCTGATCCTGGGAGT	0.447	25.72	25
18391	GCTGATCCTGGGAGTAGTG	0.464	33.36	25
18392	GATCCTGGGAGTAGTGACT	0.646	44.69	41.67
18393	CTGGCCCAAGACAAGGGCA	0.370	12.95	16.67
18394	AAGACAAGGGCAAGGGTCA	0.539	58.09	58.33
18395	TCACAGGCCATCTACTTGA	0.671	48.74	83.33
18396	TTGTCGAACTTCTTATGGC	0.886	86.96	66.67
18397	TATGGCTGTAGAGCTTGTT	0.766	61.69	75
18398	CCAGTCACACAGGAAGGAG	0.475	51.08	33.33
18399	GGAGCCCACCCCGATGGAG	0.385	33.36	16.67
18400	GAGGTCAGAGCAGTGGCCA	0.570	36.96	33.33
18401	GCAGTGGCCAGATTGATGA	0.431	38.04	50
18402	GGGAATGAGACTGAATTTC	0.397	50.18	58.33
18403	GAATGAGACTGAATTTCCC	0.726	77.97	50
18404	GAGACTGAATTTCCCTGCC	0.515	60.88	33.33
18405	CCATGCACGATAACATCGA	0.574	41.19	50
18406	ATTCGAATCCCATAGGCTT	0.760	62.23	58.33
18407	GATGAGAGTTCGAGTGGTG	0.647	67.81	41.67
18408	GAGTTCGAGTGGTGGTAGT	0.436	38.31	41.67
18409	GTAGTGCCGTTTATCTTGT	0.506	33.81	50
18410	TATCTTGTAATACTTGGCA	0.768	67.99	83.33
18411	ATCTTGTAATACTTGGCAA	0.567	61.6	66.67
18412	GGCAAACCTGAAGTTGTAG	0.570	39.3	41.67
18413	AGCCTGAGGAGGCAGGGTC	0.619	59.53	50
18414	TTGCACTCTGATTCAGACA	0.809	48.65	66.67
18415	ACAAGTCCAGGTCACAGTT	0.386	38.22	50
18416	GTCCAGGTCACAGTTCCAG	0.703	58.99	41.67
18417	GTTCCAGTTGATGATGACT	0.337	42.45	41.67
18418	ATGATGACTCCAATGACAC	0.590	86.96	66.67
18419	GTTCTGTGAAGTTCTCTCC	0.617	67.27	66.67
18420	GTTCTCTCCTGCCTTCTCA	0.323	48.74	50
18421	TCCTGCCTTCTCAACAATG	0.614	50	50
18422	CAACAATGAAACCCAGCCT	0.649	44.69	58.33
18423	ATGGGTCAGAGTCCTGATC	0.813	58.54	41.67
18424	ATGTGCAATGCTTGAGGTA	0.723	58.09	58.33
18425	TCACTCTTCTGGCTTGCAA	0.728	52.79	66.67
18427	GAACTTGAACTTGGGGTAG	0.628	65.02	58.33
18428	TGGATGCTGTTCTTGATGA	0.537	33.72	66.67
18429	GATGAGGATGGTGAAATTT	0.236	42.45	58.33
18430	CCCAGAAAATGGTTGTCAG	0.403	57.01	33.33
18431	GAAAATGGTTGTCAGAAGT	0.335	49.1	58.33
18433	GTTCCATCCTCCACCGGGC	0.484	61.24	33.33
18434	GACACCTCGCAGGTCTTGG	0.687	63.4	41.67
18435	ATAGGGTACACAGTGCCCT	0.550	67.99	58.33
18436	AGTGCCCTGTGCGAATCCC	0.660	50.36	33.33
18437	TGCGAATCCCATTGCCTTG	0.644	58.27	58.33
18438	CATTGCCTTGCATGTCCAG	0.552	56.74	33.33
18439	CCTTGCATGTCCAGCTGTC	0.353	57.73	33.33
18440	TCTGAATGGCAGGTAGAGC	0.794	75.36	58.33
18441	GAATGGCAGGTAGAGCTGT	0.423	34.17	41.67
18442	TGGCAGGTAGAGCTGTGAA	0.517	42	50
18443	ATGTTCCCAAGGTCTGGGA	0.504	42.63	58.33
18444	AAGGGGTAACCTCGATCCT	0.768	48.65	41.67
18445	TCGATCCTGGTGATGATGC	0.785	56.29	58.33
18446	GATGATGCTGACTACACTG	0.784	65.02	50
18447	GGGGCTTTACGTATTCCTC	0.349	47.57	16.67
18448	GTCCCACACTTTGTCTTCC	0.695	45.59	25
18449	TCCCACACTTTGTCTTCCG	0.646	61.06	50
18450	TGGTGATCCCCTTGACTTT	0.662	42.36	66.67
18451	CTTGACTTTGGTGATGATG	0.715	65.02	50
18452	TGATGATGGAGCTCTCCGG	0.542	74.19	66.67
18453	CCGGTCTCGCTGTCCTGGT	0.495	14.93	25

18454	GTCTCGCTGTCCTGGTAGC	0.605	42.18	25
18455	CTGGTAGCTTTTCTGCACG	0.805	60.52	33.33
18456	TGGTAGCTTTTCTGCACGA	0.742	27.43	58.33
18457	TTCTGCACGATGAAGACGT	0.852	60.34	75
18458	GAAGTAAAGCAGGATGAGA	0.613	45.23	50
18459	CAGGCGCCGATTCCGCACC	0.693	37.77	25
18460	TAGCCTAGTCCATGCTCTA	0.738	51.44	58.33
18461	TCCATGCTCTAGCTGTTTC	1.003	56.29	66.67
18462	ATGCTCTAGCTGTTTCTAT	0.918	54.95	66.67
18463	TGCTCTAGCTGTTTCTATG	0.953	74.01	58.33
18464	CTAGCTGTTTCTATGGCTT	0.904	48.74	41.67
18465	GCTGTTTCTATGGCTTGGG	0.602	54.32	41.67
18466	TGTTTCTATGGCTTGGGCT	0.917	59.44	75
18467	GTTTCTATGGCTTGGGCTT	0.611	51.89	50
18468	TTTCTATGGCTTGGGCTTC	0.903	82.01	83.33
18469	TTCTATGGCTTGGGCTTCG	0.841	77.88	75
18470	TCTATGGCTTGGGCTTCGT	0.775	53.15	66.67
18471	TATGGCTTGGGCTTCGTTG	0.940	88.31	66.67
18472	TTGGGCTTCGTTGGTCTTC	0.854	71.22	58.33
18473	CTTCGTTGGTCTTCCACTG	0.780	65.02	33.33
18474	GGTCTTCCACTGCTCCGCT	0.439	17.09	25
18475	GTCTTCCACTGCTCCGCTA	0.402	19.6	33.33
18476	CTGCTCCGCTACATCATTT	0.432	23.02	41.67
18477	TGCTCCGCTACATCATTTG	0.642	67.18	75
18478	GCTCCGCTACATCATTTGC	0.498	37.14	25
18479	CGCTACATCATTTGCTAAT	0.342	30.4	50
18480	GCTACATCATTTGCTAATG	0.302	58.72	41.67
18481	CTACATCATTTGCTAATGG	0.779	56.38	50
18482	CATCATTTGCTAATGGATC	0.613	71.31	50
18483	ATCATTTGCTAATGGATCA	0.702	55.31	75
18484	TGCTAATGGATCATCTGGA	0.859	50.9	75
18485	TGGATCATCTGGATTGGGA	0.784	51.44	66.67
18486	GGATCATCTGGATTGGGAG	0.587	65.02	33.33
18487	GATAGCAGAACTGTGCGGA	0.635	60.43	50
18488	ATAGCAGAACTGTGCGGAT	0.542	59.44	58.33
18489	CAGAACTGTGCGGATCTGC	0.863	57.1	50
18490	ATCTGCAGTGCTGGGGACC	0.622	76.89	50
18491	CTGCAGTGCTGGGGACCAC	0.594	50.45	33.33
18492	GCAGTGCTGGGGACCACTT	0.482	10.79	25
18493	GGGGACCACTTATCTTTCA	0.345	3.78	33.33
18494	GACCACTTATCTTTCAAAA	0.355	37.95	41.67
18495	CTTATCTTTCAAAATATCT	0.571	45.32	58.33
18496	TTATCTTTCAAAATATCTA	0.806	70.86	75
18497	TATCTTTCAAAATATCTAG	0.856	80.49	83.33
18498	TTTCAAAATATCTAGACAT	0.759	60.7	75
18499	CTAGACATATTCTTCCCAA	0.528	51.53	50
18500	TTCTTCCCAACTTGTCTAC	0.940	65.2	83.33
18501	TTCCCAACTTGTCTACATT	0.938	57.73	58.33
18502	CCAACTTGTCTACATTAGG	0.694	58.72	41.67
18503	TTGTCTACATTAGGATGAT	0.464	58.09	66.67
18504	CTACATTAGGATGATAAAT	0.492	38.67	41.67
18505	TAGGATGATAAATTTTGGT	0.816	53.42	66.67
18506	AAATTTTGGTCATGAAACG	1.000	88.67	75
18507	AATTTTGGTCATGAAACGT	0.921	66.1	75
18508	GTCATGAAACGTACTTTAG	0.795	63.67	58.33
18509	TTAGGGGCTGCCATTGGGT	0.640	61.96	58.33
18510	GGCTGCCATTGGGTATTCT	0.447	16.73	25
18511	GGGTATTCTTCTGGAAGGA	0.369	25.36	41.67
18512	TATTCTTCTGGAAGGAATA	0.830	66.1	75
18513	CTTCTGGAAGGAATAGTTC	0.875	71.31	58.33
18514	CTGGAAGGAATAGTTCAAG	0.848	62.77	50
18515	TGGAAGGAATAGTTCAAGT	0.961	48.65	66.67
18516	GAAGGAATAGTTCAAGTTT	0.436	45.23	50
18517	GGAATAGTTCAAGTTTAAA	0.299	38.67	41.67
18518	AGTTCAAGTTTAAAAGTCC	0.872	78.51	66.67
18519	AAAAGTCCCTCCCTCAAAG	0.706	80.04	58.33
18520	GAATCCTGAGGGCCAGCAA	0.502	45.32	41.67
18521	GAGGGCCAGCAATGACCAC	0.541	35.52	16.67
18522	GGGCCAGCAATGACCACAT	0.471	17.18	25
18523	GCCAGCAATGACCACATGA	0.506	28.15	33.33
18524	CCAGCAATGACCACATGAA	0.395	34.53	41.67
18525	CAGCAATGACCACATGAAA	0.398	31.65	41.67
18526	GCAATGACCACATGAAAAT	0.471	41.19	58.33
18527	ACATGAAAATAACGGGCGT	0.624	55.94	58.33
18528	ATGAAAATAACGGGCGTTG	0.871	82.91	83.33
18529	GGGCGTTGCTCTCATCTGG	0.658	37.5	25
18530	TGCTCTCATCTGGTTCGGC	0.826	52.52	41.67
18531	CTCTCATCTGGTTCGGCTT	0.883	38.31	33.33
18532	ATCTGGTTCGGCTTTGATG	0.847	84.17	58.33
18533	TGGTTCGGCTTTGATGCCA	0.816	42.36	58.33
18534	CGGCTTTGATGCCAGGAAC	0.782	37.5	25
18535	GCTTTGATGCCAGGAACTG	0.614	61.51	41.67

18536	TTTGATGCCAGGAACTGGT	0.974	61.69	83.33
18537	TGATGCCAGGAACTGGTTC	0.360	67.09	58.33
18538	GATGCCAGGAACTGGTTCT	0.607	47.48	50
18539	TTTTCTTACACAGCGGGCA	0.943	68.35	83.33
18540	CTTACACAGCGGGCAACTT	0.790	34.17	33.33
18541	TTACACAGCGGGCAACTTT	0.939	62.23	83.33
18542	GGGCAACTTTTCTTTTAAA	0.435	10.07	25
18543	ACTTTTCTTTTAAATTCTC	0.564	57.01	50
18544	TTTTCTTTTAAATTCTCCA	0.742	70.86	75
18545	AAATTCTCCATTTCTATCT	1.020	64.57	83.33
18546	TTTCTATCTTCCCTCCATT	0.859	65.74	75
18547	TTCCCTCCATTCTTTCGCA	0.727	40.02	50
18548	CCCTCCATTCTTTCGCAGC	0.638	46.94	25
18549	CCTCCATTCTTTCGCAGCA	0.344	29.5	25
18550	TCTTTCGCAGCATCAACAT	1.042	53.15	75
18551	CTTTCGCAGCATCAACATT	0.935	34.17	41.67
18552	CAGCATCAACATTAGCAGG	0.975	39.3	33.33
18553	AGCATCAACATTAGCAGGT	0.939	47.39	66.67
18554	AACATTAGCAGGTGAGTCT	0.811	60.34	83.33
18555	GCAGGTGAGTCTCCATTAG	0.719	52.07	33.33
18556	TGAGTCTCCATTAGGGTCT	0.898	48.74	83.33
18557	AGGGTCTGCCAGCATAGAA	0.610	35.7	41.67
18558	CCAGCATAGAAATGACACT	0.476	31.74	41.67
18559	AGCATAGAAATGACACTAA	0.540	44.6	75
18560	ATAGAAATGACACTAATCA	0.785	67	75
18561	GACACTAATCATGATGGTT	0.654	41.1	41.67
18562	ACACTAATCATGATGGTTT	0.669	57.82	75
18563	ACTAATCATGATGGTTTCC	0.544	60.79	66.67
18564	ATGATGGTTTCCACAGTGT	0.893	55.31	75
18565	GATGGTTTCCACAGTGTGG	0.861	65.02	41.67
18566	ATGGTTTCCACAGTGTGGA	0.853	57.19	66.67
18567	GGTTTCCACAGTGTGGATA	0.412	29.77	50
18568	TCCACAGTGTGGATAGGGA	0.621	48.65	50
18569	CCACAGTGTGGATAGGGAG	0.629	48.29	33.33
18570	GTGTGGATAGGGAGCCAGC	0.640	59.89	33.33
18571	GGGAGCCAGCGTTCCTCTG	0.425	29.23	16.67
18572	AGCCAGCGTTCCTCTGGCT	0.378	17	33.33
18573	GCCAGCGTTCCTCTGGCTT	0.359	27.88	25
18574	CAGCGTTCCTCTGGCTTTT	0.326	31.65	33.33
18575	GCGTTCCTCTGGCTTTTCA	0.359	16.73	33.33
18576	TCCTCTGGCTTTTCATAAC	0.493	64.93	58.33
18577	TCTGGCTTTTCATAACCAT	0.392	46.49	58.33
18578	CTGGCTTTTCATAACCATA	0.318	31.65	33.33
18579	TTTTCATAACCATACTTAT	0.693	62.32	75
18580	TTTCATAACCATACTTATC	0.877	81.03	66.67
18581	CCATACTTATCTTCCCCAG	0.550	58.72	50
18582	TACTTATCTTCCCCAGGCT	0.786	55.31	75
18583	TATCTTCCCCAGGCTCATG	0.760	67.09	58.33
18584	ATCTTCCCCAGGCTCATGA	0.764	48.92	66.67
18585	GGCTCATGAAGAATAGAAA	0.295	33.9	41.67
18586	AATAGAAATGCACACATCA	0.748	68.88	75
18587	TAGAAATGCACACATCACC	0.947	80.13	83.33
18588	AGAAATGCACACATCACCA	0.850	59.44	66.67
18589	TTTTATCAACATTTGGGTG	0.731	80.04	75
18590	TCAACATTTGGGTGCCAGA	0.734	53.15	66.67
18591	AACATTTGGGTGCCAGATT	0.734	55.31	66.67
18592	GTGCCAGATTTCTGTAATG	0.501	54.23	33.33
18593	ATTTCTGTAATGAATTTCA	0.616	64.57	75
18594	TAATGAATTTCATTTTAGG	0.696	93.97	66.67
18595	ATGAATTTCATTTTAGGAG	0.879	82.64	66.67
18596	GAATTTCATTTTAGGAGGT	0.395	34.17	50
18597	ATTTTAGGAGGTCGGAGGG	0.624	88.67	66.67
18598	GAGGTCGGAGGGGATAATC	0.847	50.45	33.33
18599	AGGTCGGAGGGGATAATCT	0.842	48.65	50
18600	CACCTTCATAAAGTGTATC	0.660	47.84	41.67
18601	ACCTTCATAAAGTGTATCT	0.787	47.39	75
18602	TAAAGTGTATCTGGAGGGC	0.752	88.67	75
18603	GTGTATCTGGAGGGCCAAT	0.361	23.38	33.33
18604	GGAGGGCCAATAATAAGGA	0.447	23.11	33.33
18605	GGCCAATAATAAGGACTTC	0.876	41.28	41.67
18606	GCCAATAATAAGGACTTCC	0.710	52.97	58.33
18607	TAAGGACTTCCCATCGGTA	0.931	50.81	58.33
18608	GGACTTCCCATCGGTAGAG	0.683	35.61	33.33
18609	ACTTCCCATCGGTAGAGAT	0.677	38.22	50
18610	CCATCGGTAGAGATCATTG	0.867	65.02	50
18611	ATCGGTAGAGATCATTGTC	1.032	74.01	50
18612	CGGTAGAGATCATTGTCAT	0.405	34.44	41.67
18613	TTGTCATCTATTAAACCTG	0.975	80.13	75
18614	CATCTATTAAACCTGCAGA	0.606	50.99	58.33
18615	TCTATTAAACCTGCAGAAA	0.517	55.94	75
18616	TTAAACCTGCAGAAAAGCC	0.833	73.74	75
18617	GAAAAGCCTTCCACTGGAT	0.654	34.17	50

18618	CACTGTCTGGCTATCCTGT	0.552	29.68	33.33
18619	CTGTCTGGCTATCCTGTCG	0.780	57.1	41.67
18620	TGTCGTGTTCTGCTCTGTT	0.769	52.79	58.33
18621	TCGTGTTCTGCTCTGTTGG	0.888	71.22	58.33
18622	GTTGGTCAAATACTGAGTG	0.871	58.63	41.67
18623	TGGTCAAATACTGAGTGGC	0.909	69.96	66.67
18624	TCAAATACTGAGTGGCTAT	0.887	55.94	83.33
18625	GCTTCCAACCAGAGGATCC	0.598	57.73	41.67
18626	TCCAACCAGAGGATCCGCA	0.758	32.19	58.33
18627	CAGAGGATCCGCAGGGTTG	0.563	59.89	41.67
18628	GGATCCGCAGGGTTGCAGT	0.711	38.67	33.33
18629	GGGTTGCAGTCTGTCAAAA	0.503	16.73	33.33
18630	CTGTCAAAAGGGAACAAAT	0.478	41.1	41.67
18631	TGTCAAAAGGGAACAAATA	0.452	49.28	66.67
18632	AAAAGGGAACAAATAGACA	0.860	72.39	66.67
18633	AGGGAACAAATAGACAGCA	0.734	23.02	50
18634	ACAAATAGACAGCAAAACC	0.948	78.51	66.67
18635	ATAGACAGCAAAACCTTTG	0.924	87.05	83.33
18636	GCAAAACCTTTGAAATAGT	0.343	21.22	41.67
18637	AAATAGTCAAAGCGGGACT	0.567	68.35	83.33
18638	ATAGTCAAAGCGGGACTCC	0.692	84.8	66.67
18639	AGTCAAAGCGGGACTCCAG	0.720	68.08	50
18640	GGGACTCCAGTTGTCTTTA	0.438	16.73	33.33
18641	ACTCCAGTTGTCTTTAAGG	0.589	75.36	50
18642	GGATGTCCAGACAGATGAC	0.438	43.79	25
18643	TGTCCAGACAGATGACTCC	0.756	71.31	66.67
18644	CAGACAGATGACTCCCTGA	0.490	38.31	41.67
18645	AGACAGATGACTCCCTGAC	0.834	74.1	58.33
18646	AGATGACTCCCTGACTGTT	0.585	38.22	50
18647	ATGACTCCCTGACTGTTGA	0.747	46.67	58.33
18648	TGACTCCCTGACTGTTGAT	0.705	31.56	58.33
18649	ACTCCCTGACTGTTGATGT	0.699	52.79	58.33
18650	CCCTGACTGTTGATGTTGC	0.661	39.3	25
18651	CTGTTGATGTTGCAGTGAT	0.697	41.1	50
18652	AGTGATAGATTCTGGTGCG	0.734	71.85	58.33
18653	GATAGATTCTGGTGCGGAA	0.425	49.1	50
18654	GATTCTGGTGCGGAAAGTA	0.492	49.1	50
18655	TCTGGTGCGGAAAGTAACC	0.914	69.06	50
18656	GGTGCGGAAAGTAACCTTT	0.507	31.74	50
18657	CGGAAAGTAACCTTTGGTG	0.588	56.47	50
18658	AACCTTTGGTGGCTTAAAT	0.642	54.95	58.33
18659	TGGTGGCTTAAATGGATAA	0.287	29.68	66.67
18660	GCTTAAATGGATAATCTGA	0.382	38.94	58.33
18661	CTGATGAAAATGTGATATC	0.576	65.92	58.33
18662	TGAAAATGTGATATCCAGA	0.688	59.44	75
18663	AAAACACACCACCTTCATA	0.501	57.46	58.33
18664	ACACCACCTTCATATACAG	0.368	54.14	41.67
18665	CAGAACCCGGTGGACCAAG	0.684	42.18	33.33
18666	TGGACCAAGTATAGTTGAT	0.595	45.14	58.33
18667	GACCAAGTATAGTTGATCT	0.528	37.95	50
18668	TATAGTTGATCTCCATTCA	0.905	66.1	83.33
18669	ATTCATAAATGTTATCTCC	0.758	81.03	66.67
18670	TCATAAATGTTATCTCCTT	0.520	52.16	66.67
18671	TATCTCCTTTAGGCCCAGC	0.672	67.09	58.33
18672	TCCTTTAGGCCCAGCACTG	0.732	63.94	58.33
18673	AGGCCCAGCACTGCAATTA	0.473	40.74	50
18674	CCAGCACTGCAATTAGGAG	0.511	43.44	16.67
18675	GCACTGCAATTAGGAGGAG	0.343	37.14	25
18676	CACTGCAATTAGGAGGAGG	0.626	63.67	33.33
18677	ATTAGGAGGAGGATCAAGG	0.816	100	75
18678	ATCAAGGGTTATTTCAGCT	0.820	61.6	66.67
18679	GGTTATTTCAGCTAGCTCC	0.805	60.97	66.67
18680	TATTTCAGCTAGCTCCTTC	0.885	97.75	91.67
18681	TTCAGCTAGCTCCTTCTGA	0.874	61.6	75
18682	CAGCTAGCTCCTTCTGAAT	0.423	31.65	41.67
18683	AGCTAGCTCCTTCTGAATT	0.712	33.72	50
18684	TTCTGAATTCTTTTAGCAC	0.815	86.96	66.67
18685	ACTAGTGGATAACTTAGCA	0.828	59.44	58.33
18686	GCAGTGGTTTTGCTAGAGA	0.359	35.79	41.67
18687	GTGGTTTTGCTAGAGAGTT	0.524	31.65	41.67
18688	GTTTTGCTAGAGAGTTTGG	0.698	56.74	58.33
18689	TTTGCTAGAGAGTTTGGTG	0.842	91.1	66.67
18690	TTGCTAGAGAGTTTGGTGT	0.901	57.19	83.33
18691	CTGGGTGGCAGAAGGTTTT	0.321	33.9	41.67
18692	TCTTTCCTCTTGTTCTTCA	0.893	44.51	75
18693	TCCTCTTGTTCTTCAGGCT	0.890	42.36	58.33
18694	TGGAGCGGCTGGGTCTCGC	0.692	67.45	50
18695	CGGCTGGGTCTCGCTGGTC	0.547	37.5	25
18696	CGCATCTGAACTGCCACTG	0.530	46.4	41.67
18697	GTCCTTCGGGCGGCTGAGG	0.552	35.52	25
18698	CCTTCGGGCGGCTGAGGGA	0.466	32.37	33.33
18699	TTTCATATTTTCTTTAGAC	0.753	87.32	66.67

18700	TTCTTTAGACATCATTAGG	0.951	80.67	75
18701	TCTTTAGACATCATTAGGC	0.945	84.26	83.33
18702	AGACATCATTAGGCGCCGA	0.788	31.56	41.67
18703	TTAGGCGCCGAAGCTCTTG	0.841	78.24	66.67
18704	TAGGCGCCGAAGCTCTTGC	0.826	61.06	58.33
18705	CGAAGCTCTTGCAGGACAA	0.519	36.15	33.33
18706	CTCTTGCAGGACAACTTTG	0.715	45.95	50
18707	TCTTGCAGGACAACTTTGA	0.898	58.18	83.33
18709	GGACAACTTTGATGCTATA	0.470	14.57	33.33
18710	ACAACTTTGATGCTATATG	0.777	84.26	66.67
18711	CAACTTTGATGCTATATGA	0.490	42.45	58.33
18712	CTTTGATGCTATATGAATT	0.700	51.62	41.67
18713	TGATGCTATATGAATTCTG	0.900	77.97	75
18714	GATGCTATATGAATTCTGC	0.820	74.1	50
18715	TGCTATATGAATTCTGCCA	0.897	47.39	75
18716	GAATTCTGCCATTTTGCTA	0.630	55.4	58.33
18717	TTCTGCCATTTTGCTAGCA	0.714	40.38	58.33
18718	TGCCATTTTGCTAGCACTG	0.875	58.27	58.33
18720	TTGCTAGCACTGATATGGC	0.921	77.52	66.67
18721	GCTAGCACTGATATGGCTC	0.909	67.81	33.33
18722	CTAGCACTGATATGGCTCT	0.903	29.77	50
18723	TAGCACTGATATGGCTCTT	0.844	48.65	66.67
18724	TATGGCTCTTGGGTCCACC	0.841	84.53	58.33
18725	TTGGGTCCACCACTCCATT	0.835	40.02	41.67
18726	CCACCACTCCATTAGAACT	0.568	14.57	25
18727	GTTACAAATCTTACAAAGG	0.638	74.46	50
18728	GGTCCACATTCTATTTTAA	0.388	20.86	33.33
18729	TATTTTAAGGCTGTATATT	0.829	71.4	75
18731	TTTTAAGGCTGTATATTCG	0.894	94.96	91.67
18732	TAAGGCTGTATATTCGGTT	0.961	67.99	75
18733	CATAAATTGTTCTTGGAGG	0.631	77.97	58.33
18734	ATAAATTGTTCTTGGAGGC	0.765	88.67	75
18735	AATTGTTCTTGGAGGCCCA	0.571	66.1	66.67
18736	GGAGGCCCAATTATCATCC	0.705	45.68	33.33
18737	TTATCATCCCTGTCCATCT	0.900	66.1	83.33
18738	CATCCCTGTCCATCTTGTA	0.527	42.45	41.67
18739	TCATGTCTTCGTCATCTTC	0.950	60.79	66.67
18740	TGTCTTCGTCATCTTCTAG	0.888	60.43	66.67
18741	GTCTTCGTCATCTTCTAGA	0.580	46.85	58.33
18742	TCTTCGTCATCTTCTAGAC	0.920	75.72	66.67
18743	TTCGTCATCTTCTAGACCC	0.963	74.01	66.67
18744	CGTCATCTTCTAGACCCCA	0.388	14.57	25
18745	CATCTTCTAGACCCCAGCT	0.730	27.52	33.33
18746	ATCTTCTAGACCCCAGCTA	0.828	57.55	75
18747	GCTAACTGTGCCATCTCCT	0.609	42.45	41.67
18748	TAACTGTGCCATCTCCTAC	0.888	88.31	83.33
18749	TGTGCCATCTCCTACTCCT	0.923	49.28	50
18750	CCATCTCCTACTCCTTTCT	0.586	23.47	50
18751	TACTCCTTTCTGGCCTTCT	0.861	55.31	75
18753	GGCCTTCTTCGAGTTCTTC	0.538	32.64	25
18754	CCTTCTTCGAGTTCTTCCA	0.599	38.4	50
18755	GTTCTTCCAACAGTCGGAA	0.523	36.06	50
18756	TTCCAACAGTCGGAAATTG	0.953	56.29	66.67
18757	CAACAGTCGGAAATTGCGA	0.856	48.74	50
18758	CAGTCGGAAATTGCGAGGG	0.845	59.35	41.67
18759	TCGGAAATTGCGAGGGACT	0.960	38.49	50
18760	ATTGCGAGGGACTTTTACT	0.910	68.53	58.33
18761	TGCGAGGGACTTTTACTCC	0.887	64.57	58.33
18762	GAGGGACTTTTACTCCTTT	0.656	23.02	33.33
18764	TTTACTCCTTTGCAGTGAA	0.814	51.17	66.67
18765	TACTCCTTTGCAGTGAAAT	0.950	61.6	66.67
18766	CTCCTTTGCAGTGAAATTT	0.458	33.9	58.33
18767	CCTTTGCAGTGAAATTTTC	0.837	43.79	50
18768	TTGCAGTGAAATTTTCTAG	0.960	79.77	83.33
18769	GCAGTGAAATTTTCTAGGT	0.365	32.28	41.67
18770	CAGTGAAATTTTCTAGGTT	0.494	47.39	41.67
18771	GTGAAATTTTCTAGGTTCA	0.430	38.31	58.33
18772	GAAATTTTCTAGGTTCAAG	0.535	77.97	58.33
18773	ATTTTCTAGGTTCAAGTCT	0.714	66.1	83.33
18774	TCTAGGTTCAAGTCTTCCT	0.780	55.4	75
18775	AGTCTTCCTTCATCACTCA	0.623	31.56	58.33
18776	GCTTGGTTCGTTTCCAAGG	0.858	58.72	33.33
18777	CAAGGGAACACAATGGGCA	0.954	53.78	41.67
18778	GGTGGGGCAGGCTCTGGTG	0.904	48.29	25
18779	GTGGGGCAGGCTCTGGTGT	0.728	19.24	25
18780	GTGTGAGTTTCCACTCTGG	0.953	60.88	41.67
18781	GTGAGTTTCCACTCTGGTG	0.928	60.88	33.33
18782	GTTTCCACTCTGGTGACCT	0.943	58.18	41.67
18783	CTCTGGTGACCTTTGACTC	0.859	67.18	33.33
18784	ACCTTTGACTCCCGTAGGC	0.915	61.15	50
18785	TTGACTCCCGTAGGCGTTT	0.936	40.38	58.33
18786	CACGGGTGTGCATACCCTG	0.706	50.45	25

18788	GGTGTGCATACCCTGTTGC	0.725	41.91	41.67
18789	CATACCCTGTTGCTCTCTC	0.855	63.04	33.33
18790	CCTGTTGCTCTCTCCCGCT	0.435	25.72	33.33
18791	GTTGCTCTCTCCCGCTGCC	0.619	46.31	25
18793	CTCTCTCCCGCTGCCCCTG	0.662	42.18	25
18795	CACTCCGACCCCTGACAGG	0.725	57.1	33.33
18796	ACCCCTGACAGGCAGGGGA	0.422	34.17	41.67
18797	GGGAAAGAAACTGTAGTCT	0.556	33.9	58.33
18798	AAAGAAACTGTAGTCTGCC	0.860	91.1	66.67
18799	AACTGTAGTCTGCCAGATT	0.817	58.09	58.33
18800	CAGATTCCACACCATGTGG	0.402	45.95	41.67
18801	CACACCATGTGGCAGAGAG	0.467	59.89	33.33
18802	ATGTGGCAGAGAGACAGAG	0.623	65.2	50
18803	GTGGCAGAGAGACAGAGCC	0.458	52.7	33.33
18804	TGGCAGAGAGACAGAGCCA	0.765	34.71	50
18805	GCAGAGAGACAGAGCCACC	0.715	51.08	33.33
18807	CCTGCTGCCTGTCCCCTGG	0.456	48.29	33.33
18809	CCCTGGGCTATCTCCTGCC	0.677	46.4	33.33
18810	CTGCCACAGACCAGCTTCA	0.444	15.2	33.33
18811	CTTCAGAGAAGGAAGGAGT	0.507	47.48	58.33
18812	AGAGAAGGAAGGAGTCCTC	0.812	71.31	58.33
18813	GCTAGAGATGGAAGTCTCA	0.329	36.15	50
18814	GAAGTCTCAGAAGCAGGGA	0.679	42.45	41.67
18815	AGGGAAGGTGGATGAAGGC	0.857	54.5	41.67
18816	GCAGAAGGGCTCCAAAGCC	0.737	48.29	33.33
18817	GCTCCAAAGCCTATGGGGA	0.358	34.8	25
18818	TGGGGATATTGCTCAGAAG	0.930	58.27	50
18819	TATTGCTCAGAAGTTAAGT	0.907	72.39	75
18820	GTTAAGTAAGGAGCTCATT	0.708	49.1	58.33
18821	CTCATTCCTCAGAGGCAAG	0.598	45.95	33.33
18822	AAGAATTCCTGGGTGTTCT	0.845	61.6	75
18823	TGGGTGTTCTGTAAAGGGT	0.865	35.7	58.33
18824	TAAAGGGTGGGGCGGATTG	0.802	84.89	75
18826	CCTAAGTGGGCACCCTAAA	0.488	36.15	41.67
18827	AGACGCCTCAAGGGAGGCA	0.341	30.04	41.67
18828	GACGCCTCAAGGGAGGCAA	0.373	30.13	33.33
18830	CTGAATCTCCAACTGCAAA	0.486	29.68	41.67
18831	CTTCTAAACTGGTGGAATC	0.840	67.81	50
18832	AATCTGGAGCAAGTTTCTT	0.790	65.74	66.67
18833	TCTGGAGCAAGTTTCTTCT	0.900	55.04	75
18834	TTTCTTCTCAGAGTCCACA	0.855	53.06	75
18835	TCAGAGTCCACAAGGAACA	0.943	48.74	75
18836	GTCCACAAGGAACATCAGT	0.552	34.44	41.67
18837	ACGAACTTTCTCAGCCCCG	0.952	61.15	50
18838	GAACTTTCTCAGCCCCGGG	0.854	61.24	41.67
18839	TCTCAGCCCCGGGATCACT	0.949	27.79	50
18840	GGTACAACTCTCCCTCAAG	0.634	61.51	33.33
18841	CAACTCTCCCTCAAGCCTA	0.767	42.45	41.67
18842	TCTCCCTCAAGCCTAAATC	0.908	77.61	66.67
18843	TCCCTCAAGCCTAAATCCA	0.814	38.49	58.33
18844	CCAGAAAGAGGGGACTAAG	0.875	54.86	33.33
18845	CTAAGGGCTCCTCAGGGAC	0.858	67.9	41.67
18846	GGGAGACTCCAGAGAGCAA	0.584	6.65	16.67
18847	ACTCCAGAGAGCAAGAGTA	0.579	48.74	58.33
18848	AGAGTAGATGGAGGTCACA	0.968	46.49	58.33
18849	ATGGAGGTCACAGGAGGGC	0.890	69.69	58.33
18850	ATGGGAGAGGAAGCACACA	1.042	48.65	41.67
18851	CACAGTTCACAGGAACAAT	0.769	38.31	41.67
18852	CAGTTCACAGGAACAATGA	0.667	41.1	58.33
18853	TCACAGGAACAATGAAACG	0.542	69.06	66.67
18854	TGAAACGACCACGAACGAC	0.393	75.72	66.67
19013	TTTGTTTTCAACATGGTAA	0.913	64.21	83.33
19014	CAACATGGTAAGGGTTTTG	0.703	67.27	66.67
19015	CATGGTAAGGGTTTTGACC	0.845	74.1	41.67
19016	ATGGTAAGGGTTTTGACCT	0.857	57.73	58.33
19017	CCTCAAATGCTTATCAGGT	0.468	38.58	41.67
19018	CGCCTCTTCCTTGAAGTTT	0.663	18.71	41.67
19019	ATTGCAGGTTTTCCTTCTC	0.791	82.01	58.33
19020	ACACAAAGGGTATTGCAAG	0.741	78.15	58.33
19021	CAAAGGGTATTGCAAGGAT	0.663	49.1	50
19022	CCTCAAATATATGGGGATA	0.486	32.28	41.67
19023	TCAAATATATGGGGATACC	0.763	78.51	75
19024	CAACTTGTACAGACGAACA	0.848	42.45	50
19025	AACTTGTACAGACGAACAA	0.555	57.55	75
19026	ACTTGTACAGACGAACAAG	0.543	78.51	58.33
19027	TACAGACGAACAAGACCTC	0.727	65.2	66.67
19028	GACCTCATCTCCCATACCG	0.848	53.24	33.33
19029	TCTCCCATACCGGGTTCCG	0.498	68.08	50
19030	AAAATGAGTTAGGCTGTCC	0.628	91.46	83.33
19031	CAAACTCATCAGGCAGGTT	0.520	34.17	41.67
19032	GCAGGTTATAATAGATAGT	0.422	31.74	41.67
19033	AGTGTAGCACATAAAATTT	0.468	42.72	58.33

19034	ATACATCTCTCCTCCTTTG	0.992	67.09	66.67
19035	TCCTCCTTTGGTCTCACAC	0.980	71.22	50
19036	AGAAACAATACAATCCTCC	1.047	87.05	83.33
19037	AAACAATACAATCCTCCGA	0.547	61.69	75
19038	TCCGACCCTTCTCCCAGGT	0.776	17	41.67
19039	CCCTTCTCCCAGGTCATCA	0.332	27.88	41.67
19041	GTCCTAGCCGTGTAAGGCC	0.600	50.45	33.33
19043	ATTGCTGCATGGAGTCCTC	0.448	82.01	50
19044	GGTGAGCTCCAACTCCAAG	0.552	39.66	25
19045	TCCAACTCCAAGTCTCAGT	0.887	44.6	75
19046	AAGTCTCAGTGTCACAGGG	0.659	62.95	41.67
19047	GTCTCAGTGTCACAGGGAC	0.430	57.1	33.33
19048	AGTGTCACAGGGACCCAAA	0.508	49.28	50
19049	GTCACAGGGACCCAAAGTT	0.268	40.56	41.67
19050	GCACCTCAAATTCAGGAAC	0.231	39.39	33.33
19051	CTCAAATTCAGGAACACAC	0.327	63.13	58.33
19052	CCAGGAATGAACGTTATTC	0.668	63.4	50
19053	ATGTGAGCGCATTTTCCAA	0.387	61.6	58.33
19054	TGTGAGCGCATTTTCCAAA	0.476	40.11	66.67
19055	CATTTTCCAAATCAAAGTT	0.503	32.64	50
19056	TTCCAAATCAAAGTTATTT	0.702	56.21	75
19057	TCCAAATCAAAGTTATTTT	0.699	47.12	75
19058	AAACTCATATCGAGGTCGT	0.700	62.23	66.67
19059	AGACCATAGCAGAAACGTA	0.627	46.49	50
19060	GAAGACAGAGGGGAAAAGA	0.353	45.23	50
19061	GAAAACAAAGGCTCTCTGG	0.896	74.46	66.67
19062	AAGGCTCTCTGGTTTCAGC	1.020	62.59	33.33
19063	TTTCAGCTTCTCCAGGGTG	0.667	67.09	58.33
19064	AGGGTGTCATGTCAGCCAA	0.654	35.7	41.67
19065	TCGCCATCACTCAACCAGA	0.757	35.7	41.67
19066	TGGAAACCGCGGACGGCTC	0.701	46.22	50
19067	GAAACCGCGGACGGCTCAC	0.722	67.9	41.67
19068	CGCGGACGGCTCACGAAGA	0.354	0	8.33
19069	ATCCGCCGCACGAGCTGCA	0.337	32.19	41.67
19070	CGAGCTGCACCAGCATCTC	0.322	48.29	25
19071	CCAGCATCTCGGCCATCTT	0.331	25.72	25
19072	GGCCATCTTCGCCATCTCC	0.724	22.57	25
19073	CCATCTTCGCCATCTCCTG	0.819	54.95	33.33
19075	CGCCCTGCAGGTGGTTGAG	0.432	37.5	16.67
19076	GAGAGCGCTCTCCTCAGAG	0.488	63.4	33.33
19077	GCTCTCCTCAGAGGCCTCT	0.693	13.04	41.67
19078	ACCTGCGTCTTGATCTGGA	0.546	48.65	41.67
19079	CATATTGTCCATCTCCCAC	0.797	77.97	58.33
19080	TCTCCCACAGCTTGCCCCG	0.696	68.08	50
19081	CTCCCACAGCTTGCCCCGG	0.449	35.52	16.67
19082	GCGCACGGGCGCGCGTCCG	0.197	37.5	25
19083	CGCGCTCGCGCGTCAGCGA	0.140	0	8.33
19084	TCTCGTGGATGACGGGCAG	0.708	65.29	50
19085	ATGACGGGCAGCAGAAACT	0.662	57.55	58.33
19086	AGCAGAAACTGCTCGAAGT	0.514	45.14	50
19087	AGCACCTCCACCTCCTCGG	0.589	63.4	50
19089	GCCGCATCTCCGCTGCTTT	0.356	21.22	25
19090	CGCATCTCCGCTGCTTTGC	0.748	44.15	41.67
19091	TCTCCGCTGCTTTGCTTGT	0.626	31.56	50
19092	TATTAACATGTAAGATTTT	0.765	47.39	75
19093	TAAGGGTTTTGACCTCAAA	0.826	59.44	66.67
19094	ACATTTATAGCTTGACTAC	0.883	78.51	75
19095	TATAGCTTGACTACATATG	1.013	90.92	83.33
19096	TTGACTACATATGAACCTT	0.854	58.09	66.67
19097	GACGCCTCTTCCTTGAAGT	0.825	37.95	25
19098	GTTTTGGGTCCATTGCAGG	0.862	77.97	50
19099	GGTATTGCAAGGATCTTTC	0.826	67.27	66.67
19101	ATATGGGGATACCAACTTG	0.625	88.67	75
19102	GAGTTAGGCTGTCCCAACA	0.856	38.31	41.67
19103	ATCAGGCAGGTTATAATAG	0.880	69.24	58.33
19104	GGCAGGTTATAATAGATAG	0.904	47.93	41.67
19106	CATAGAAACAATACAATCC	0.933	76.71	66.67
19107	TCCCAGGTCATCAGTCCTA	0.704	37.95	58.33
19108	CCCAGGTCATCAGTCCTAG	0.631	50.45	33.33
19109	CATCAGTCCTAGCCGTGTA	0.618	42.45	41.67
19110	CACAGTCCCAGATGAAGTC	0.824	48.2	33.33
19112	TTCTTCTGCCATTGCTGCA	0.729	55.31	75
19113	CTTCTGCCATTGCTGCATG	0.791	56.38	33.33
19114	TGCATGGAGTCCTCTTCTA	0.686	42.36	66.67
19116	GAACTTTCAGAGGCGGTGA	0.455	42.45	50
19117	GGCGGTGAGCTCCAACTCC	0.500	37.5	25
19118	CTCCAACTCCAAGTCTCAG	0.815	45.59	25
19119	CCAACTCCAAGTCTCAGTG	0.776	52.34	33.33
19120	GGGACCCAAAGTTCAGGGA	0.533	8.9	25
19121	GTTCAGGGAGCTATCAGGT	0.685	48.74	41.67
19122	GGGAGCTATCAGGTCACCC	0.871	50.45	25
19123	GCTATCAGGTCACCCAAGA	0.456	38.94	41.67

19124	AGGTCACCCAAGAAAGAGC	0.958	52.25	41.67
19126	GAACACACCAAAGCCAGGA	0.669	47.48	50
19127	ACACCAAAGCCAGGAATGA	0.526	49.28	58.33
19128	GTATGTGAGCGCATTTTCC	0.867	77.97	58.33
19129	AGCGCATTTTCCAAATCAA	0.681	35.7	41.67
19130	CCATAGCAGAAACGTAGTG	0.580	46.04	50
19131	AGCAGAAACGTAGTGTCAG	0.993	74.01	50
19132	AACGTAGTGTCAGTGAGAA	0.722	54.95	58.33
19133	CGTAGTGTCAGTGAGAACA	0.689	38.4	50
19134	CAGAGTGAAGACAGAGGGG	0.875	57.1	33.33
19135	AAGGAAAACAAAGGCTCTC	1.064	76.26	66.67
19136	ACAAAGGCTCTCTGGTTTC	0.860	75.72	75
19137	TCAGCCAATCGCCATCACT	0.858	49.28	50
19138	TTCTCTATCCGCCGCACGA	0.872	54.32	58.33
19139	TCTATCCGCCGCACGAGCT	0.797	34.44	58.33
19140	CCGCCGCACGAGCTGCACC	0.884	28.87	8.33
19141	TCTCGGCCATCTTCGCCAT	0.787	27.79	41.67
19142	GCGCCTTCTCGTCGGCCTT	0.631	14.93	16.67
19143	CGTCGGCCTTCTCCGCCCT	0.239	10.79	16.67
19144	CGGCCTTCTCCGCCCTGGG	0.656	37.5	16.67
19147	GCGGGGCTCGGCGCCGCCC	0.749	22.57	8.33
19148	GCCGCCCGCGCCCTGCAGG	0.609	28.87	8.33
19151	GGCCTCTACCTGCGTCTTG	0.732	37.5	33.33
19152	CTCTACCTGCGTCTTGATC	0.720	52.25	33.33
19153	TGGATCAGCATATTGTCCA	0.915	53.69	75
19154	TATTGTCCATCTCCCACAG	0.829	73.74	66.67
19155	CCATCTCCCACAGCTTGCC	0.810	42.27	33.33
19156	CCCGGTTGCGCACGGGCGC	0.553	37.5	16.67
19158	TCAGCGACGCGATGTCCTC	0.779	68.08	50
19159	CGTGGATGACGGGCAGCAG	0.395	48.29	25
19160	GTGGATGACGGGCAGCAGA	0.353	27.88	33.33
19161	CAGCAGAAACTGCTCGAAG	0.599	63.31	33.33
19162	TCCGGCTCCAGCACCTCCA	0.691	34.17	50
19163	TCCGCCAGCTCCACTGCGT	0.764	34.71	41.67
19164	CCGCCAGCTCCACTGCGTC	0.655	43.79	16.67
19165	GGCCGCATCTCCGCTGCTT	0.428	17.72	16.67
19166	GCTTTGCTTGTCTGCACGA	0.676	21.22	33.33
19167	GCTTGTCTGCACGACACTC	0.648	40.02	25
19168	GACCGAGCTCGGGCCGAGT	0.615	27.88	25
19169	GTCTTCAAAACTTCCGCCG	0.948	65.92	50
19170	AAAACTTCCGCCGCTGTTC	0.843	88.67	66.67
19330	CTGCCTCTCAGCGGATGTA	0.395	15.2	25
19332	CAGCCCTCCAGCTCATTCT	0.753	30.13	41.67
19333	AGCCCTCCAGCTCATTCTT	0.542	20.77	25
19334	TTGCGCCCCAGAACCGGCC	0.796	54.77	50
19335	GGCCCACCACCCTCCGCAC	0.524	22.57	8.33
19336	CCTCCTCTTCCCAGGAGCC	0.467	33.36	16.67
19339	GAGTCTCCTGGCTCCGTGT	0.488	19.6	33.33
19340	AGTCTCCTGGCTCCGTGTA	0.549	27.79	41.67
19341	CCGTGTAGACAGAGTTTCG	0.440	50.72	33.33
19342	TCAAAGGTGTCTTGGCGGG	0.576	71.94	66.67
19343	AGGTGTCTTGGCGGGATAG	0.707	46.22	41.67
19346	GGCCAGGTGGGTGGTTGCT	0.316	14.93	25
19347	GGGGCTGGCCCAGCTCCCC	0.192	37.5	8.33
19349	GGTAGCCATGGTGCGGTTC	0.490	40.02	33.33
19350	TCAGCAGCGGATTGGGTGG	0.815	65.29	58.33
19352	CTTCCAGTAGGCTTCCAAG	0.609	71.31	33.33
19354	AGGCATCTTCCAATTGGTT	0.500	27.07	41.67
19355	CAATTGGTTCTCGTCCAGG	0.802	77.97	50
19359	TGATGAGCCTCTGCAGTAC	0.736	75.72	66.67
19360	AGCGAGGTTTTAGAGAGCT	0.561	35.7	50
19362	GATCCGCTCGATTTCACTC	0.849	56.38	25
19363	ATTTCACTCTGTACCTCAG	0.591	73.74	58.33
19364	CACTCTGTACCTCAGCCAG	0.348	57.1	33.33
19365	GCGAGTGTTGGAGCGCTCG	0.152	44.15	33.33
19367	ATAATGTGTTTGCTGGGGT	0.634	72.39	75
19370	TTGAGCGATGGTCCCACCA	0.712	51.53	58.33
19372	GGGGCACGTGCTCTGTCGA	0.399	6.29	8.33
19373	TTCTGCTTCTGTTTGGCAC	0.821	84.17	66.67
19374	TGCTGGAGCTGAGGCGGTT	0.462	41.37	50
19375	GAGCTGAGGCGGTTTTGGC	0.541	59.53	33.33
19376	AGCAGGCGAAGGCTGTCCA	0.395	32.19	41.67
19381	GACAGGGCTGGGGATGAAA	0.164	34.53	33.33
19384	TCCTTCACCAGCCTCCCGA	0.737	49.91	66.67
19385	CAGCCTCCCGATCCACCAG	0.407	35.52	16.67
19386	TTCTCCTTGATGTGCAGGA	0.940	57.55	75
19387	ATGTGCAGGAAGTCCTTGG	0.632	82.91	66.67
19388	ACTGGCACCTCATCTCCTG	0.834	68.08	41.67
19390	ACGGATTGTAGCCAACATT	0.909	37.95	58.33
19394	CTTTCTCGAGCTGGGCTAA	0.637	34.17	41.67
19395	TTCTCGAGCTGGGCTAAGG	0.768	76.89	58.33
19396	GACACGTCGGAGTCTGACG	0.770	57.1	33.33

19397	CGGAGTCTGACGGCCGGCT	0.442	8.9	25
19398	GGCTCGTGTAGGACTCTGC	0.794	46.4	41.67
19403	GACCTTTTGAACCGCCCTT	0.577	33.9	41.67
19406	CTGTATTTGCCCTGTGGGC	0.758	57.1	41.67
19407	AGACCTCCATAGGGATCTC	0.715	54.14	33.33
19408	TGTCGTCCTTTGTAAGACT	0.987	31.56	50
19409	TCCTTTGTAAGACTGAAGT	1.119	50.9	75
19410	CCTTTGTAAGACTGAAGTC	0.929	58.72	50
19411	AAGACTGAAGTCTTCTGAA	0.925	61.6	58.33
19412	ACTGAAGTCTTCTGAATGA	0.547	46.49	66.67
19413	TGAATGATCTCCTTGATAG	1.153	84.8	83.33
19414	TGATCTCCTTGATAGGTAG	1.144	60.79	66.67
19415	ATCTCCTTGATAGGTAGGG	1.025	80.13	66.67
19416	ATAGGTAGGGTACATCACA	1.074	62.23	58.33
19417	AGGTAGGGTACATCACAGA	0.552	44.6	66.67
19418	GTAGGGTACATCACAGAAC	1.048	67.27	50
19419	TAGGGTACATCACAGAACT	1.164	51.44	58.33
19420	TCACAGAACTGGGAACAGA	0.996	49.28	66.67
19421	GAACTGGGAACAGAAGAGT	0.814	44.69	58.33
19422	CAGAAGAGTGACCCAGACC	0.738	59.89	41.67
19423	AAGAGTGACCCAGACCCAG	0.876	74.1	50
19424	GTGACCCAGACCCAGAGAG	0.731	44.42	33.33
19425	TGACCCAGACCCAGAGAGT	0.618	45.5	50
19427	CCCAGACCCAGAGAGTGGC	0.633	31.47	25
19428	CCAGACCCAGAGAGTGGCC	0.414	33.36	25
19429	GGCCAAGTTCATGAGTGGC	0.622	37.5	25
19430	TCATGAGTGGCAACAAACA	1.048	53.15	66.67
19431	GAGTGGCAACAAACAGGAA	0.376	23.38	33.33
19432	GGCAACAAACAGGAAGTTC	0.536	50.72	50
19433	GCAACAAACAGGAAGTTCA	0.337	41.19	58.33
19434	AAACAGGAAGTTCACGCCT	0.911	59.44	66.67
19435	AGGAAGTTCACGCCTGAGT	0.731	44.6	58.33
19436	GAAGTTCACGCCTGAGTCC	0.704	46.31	41.67
19437	TTCACGCCTGAGTCCTCAC	0.894	59.17	50
19438	TCACGCCTGAGTCCTCACC	0.946	52.61	50
19439	CACGCCTGAGTCCTCACCA	0.698	34.17	25
19440	ACGCCTGAGTCCTCACCAT	0.657	31.92	33.33
19441	CGCCTGAGTCCTCACCATC	0.618	40.29	25
19442	GAGTCCTCACCATCCGTCC	0.811	57.1	41.67
19443	AGTCCTCACCATCCGTCCA	0.525	27.79	33.33
19444	TCCTCACCATCCGTCCAGT	0.465	29.95	41.67
19445	CCTCACCATCCGTCCAGTA	0.326	10.79	25
19446	TCACCATCCGTCCAGTACT	0.905	46.49	50
19447	ACCATCCGTCCAGTACTCA	0.631	33.72	50
19448	CCATCCGTCCAGTACTCAT	0.419	36.15	33.33
19449	CATCCGTCCAGTACTCATC	0.874	67.27	41.67
19450	CCGTCCAGTACTCATCCTT	0.758	30.4	33.33
19451	CATCCTTGTCAAAGTGAGC	1.017	65.02	41.67
19452	TCAAAGTGAGCATCTCCGC	1.085	75.72	66.67
19453	AAAGTGAGCATCTCCGCCG	1.184	83.27	66.67
19454	GTGAGCATCTCCGCCGAGG	0.721	59.89	33.33
19455	TGAGCATCTCCGCCGAGGC	0.902	65.29	50
19456	AGCATCTCCGCCGAGGCCT	0.685	38.58	50
19457	ATCTCCGCCGAGGCCTGGC	0.361	74.1	50
19459	TCCGCCGAGGCCTGGCCCC	0.508	54.5	41.67
19460	GCCGAGGCCTGGCCCCGGT	0.110	14.93	16.67
19461	CTGGCCCCGGTGCAAAGGC	0.326	35.52	16.67
19462	TGGCCCCGGTGCAAAGGCA	0.591	17	33.33
19463	AAAGGCATGGCCTAGAGTG	1.008	84.8	50
19464	GCATGGCCTAGAGTGTTTC	0.537	52.34	41.67
19465	CATGGCCTAGAGTGTTTCC	0.897	50.09	33.33
19466	GGCCTAGAGTGTTTCCTGG	0.338	43.79	33.33
19467	CCTAGAGTGTTTCCTGGCC	0.545	54.95	33.33
19468	CTAGAGTGTTTCCTGGCCC	0.835	67.54	41.67
19469	GTTTCCTGGCCCATCAAAT	0.105	55.4	50
19470	TGGCCCATCAAATGGGAAG	0.850	63.31	50
19471	GCCCATCAAATGGGAAGTT	0.002	6.03	33.33
19472	GTTGTCTCCGTGATCTCCC	0.942	67.54	41.67
19473	TGTCTCCGTGATCTCCCCT	1.018	34.08	50
19474	CTCCGTGATCTCCCCTTGC	0.905	50.45	33.33
19475	TCCGTGATCTCCCCTTGCG	0.973	57.28	50
19476	CGTGATCTCCCCTTGCGAA	0.268	17.09	25
19477	TGATCTCCCCTTGCGAAGC	1.053	57.01	50
19478	CCTTGCGAAGCCAATTATG	0.631	58.72	41.67
19479	CTTGCGAAGCCAATTATGA	0.755	51.53	50
19481	GAAGCCAATTATGATGTCT	0.994	45.23	50
19482	AAGCCAATTATGATGTCTG	1.027	82.55	58.33
19483	AGCCAATTATGATGTCTGC	0.959	58.27	58.33
19484	CCCCAACTAACCCTCTTGA	0.408	12.32	33.33
19486	AGTGGGATTTGCATACTCC	0.967	78.15	58.33
19487	CTGGAATGCCACTTAGGAC	1.095	60.52	33.33
19488	GAATGCCACTTAGGACTGT	0.657	34.17	50

19489	TGCCACTTAGGACTGTTTG	0.812	64.57	66.67
19490	TTAGGACTGTTTGGCATTA	1.050	44.51	66.67
19491	TGGCATTAGTGAGAATTCT	1.069	35.7	66.67
19492	GCATTAGTGAGAATTCTGC	0.943	67.27	66.67
19493	CATTAGTGAGAATTCTGCA	0.729	55.4	58.33
19494	TTAGTGAGAATTCTGCAAC	1.221	93.35	83.33
19495	AGTGAGAATTCTGCAACAT	0.802	49.28	58.33
19496	GTGAGAATTCTGCAACATC	1.194	63.67	41.67
19497	GAATTCTGCAACATCTGGC	1.001	80.22	66.67
19498	TTCTGCAACATCTGGCACT	1.150	60.34	75
19499	TCTGCAACATCTGGCACTC	1.040	71.85	50
19500	TGCAACATCTGGCACTCCA	0.913	45.14	58.33
19501	AACATCTGGCACTCCACAC	0.939	77.88	58.33
19502	TCTGGCACTCCACACCTGG	0.856	68.08	58.33
19503	CTGGCACTCCACACCTGGG	0.607	35.52	16.67
19504	GCACTCCACACCTGGGCTT	0.475	13.04	33.33
19505	CACTCCACACCTGGGCTTC	0.940	59.89	41.67
19506	CCACACCTGGGCTTCTGCA	0.613	17.09	25
19507	ACACCTGGGCTTCTGCATT	0.861	52.79	41.67
19508	CTTCTGCATTATCTCCATG	0.975	56.38	41.67
19509	TCTGCATTATCTCCATGAC	1.150	69.06	58.33
19510	TGCATTATCTCCATGACAC	1.122	74.01	66.67
19511	GGACAGCTTTCCAGTCTCC	0.887	33.36	33.33
19512	GCTTTCCAGTCTCCGGCAA	0.019	17.45	33.33
19513	TTCCAGTCTCCGGCAAACC	1.028	67.45	58.33
19514	TCCGGCAAACCGAAGAACT	0.538	38.49	41.67
19515	CCGGCAAACCGAAGAACTT	0.018	21.49	16.67
19518	GAACTTCTGCATTTCCCTC	0.975	56.38	41.67
19519	AACTTCTGCATTTCCCTCA	1.037	57.55	83.33
19520	ACTTCTGCATTTCCCTCAG	0.834	75.72	58.33
19521	TCTGCATTTCCCTCAGTTT	1.161	46.49	66.67
19522	CATTTCCCTCAGTTTGTCC	0.830	63.04	58.33
19523	CCCTCAGTTTGTCCACTGC	0.323	44.15	33.33
19524	TCAGTTTGTCCACTGCACT	1.101	52.79	66.67
19525	CAGTTTGTCCACTGCACTG	1.000	60.88	41.67
19526	CCACTGCACTGGTGGCCTT	0.223	10.79	25
19527	GCACTGGTGGCCTTCTTTG	0.382	54.59	41.67
19529	ACTGGTGGCCTTCTTTGTT	0.871	52.79	50
19530	TGGTGGCCTTCTTTGTTTT	0.457	33.72	58.33
19531	TGGCCTTCTTTGTTTTAGA	0.957	42	50
19532	CTTCTTTGTTTTAGAGTCG	1.133	65.02	58.33
19533	CTTTGTTTTAGAGTCGTGA	1.080	57.64	66.67
19534	TGTTTTAGAGTCGTGAAGG	1.262	84.8	75
19535	TTTTAGAGTCGTGAAGGTA	1.100	68.88	83.33
19536	TTTAGAGTCGTGAAGGTAA	1.308	66.1	83.33
19537	TTAGAGTCGTGAAGGTAAA	1.100	59.44	75
19538	GAGTCGTGAAGGTAAAATT	0.761	40.56	50
19539	AGTCGTGAAGGTAAAATTT	0.744	46.49	58.33
19540	TCGTGAAGGTAAAATTTCC	1.269	67.72	66.67
19541	GTGAAGGTAAAATTTCCTA	0.862	46.85	58.33
19542	AGGTAAAATTTCCTAAGAT	0.796	47.66	50
19543	GGTAAAATTTCCTAAGATA	0.284	35.16	41.67
19544	AAATTTCCTAAGATAATTC	1.162	78.51	75
19545	TTTCCTAAGATAATTCTGC	1.316	93.35	83.33
19546	CCTAAGATAATTCTGCGCC	1.341	70.05	58.33
19547	CTAAGATAATTCTGCGCCT	0.660	49.1	50
19548	AAGATAATTCTGCGCCTGT	0.756	58.09	75
19549	ATAATTCTGCGCCTGTTCC	1.041	80.04	75
19550	TAATTCTGCGCCTGTTCCC	0.936	88.67	75
19551	AATTCTGCGCCTGTTCCCA	0.468	72.39	58.33
19553	CTGTTCCCACTGAAGTGCG	0.911	42.18	33.33
19554	TGTTCCCACTGAAGTGCGG	0.916	57.01	50
19555	CACTGAAGTGCGGTCACTT	0.797	47.39	33.33
19556	ACTGAAGTGCGGTCACTTC	0.405	69.06	58.33
19557	CTGAAGTGCGGTCACTTCT	0.329	38.31	50
19558	AGTGCGGTCACTTCTCCGG	0.944	67.54	50
19559	GGTCACTTCTCCGGCTTCC	0.830	48.29	41.67
19560	CACTTCTCCGGCTTCCTGG	0.722	63.4	50
19561	ACTTCTCCGGCTTCCTGGG	0.642	57.01	41.67
19562	GGCTTCCTGGGACAGTGGC	0.660	29.23	25
19563	TTCCTGGGACAGTGGCAGG	0.679	67.45	58.33
19564	TGGGACAGTGGCAGGGCCA	0.936	34.71	50
19565	AGTGGCAGGGCCAGGCAGC	0.643	68.08	41.67
19566	GGCCGAGCGGCCTCGGTCC	0.507	37.5	16.67
19567	GGCCTCGGTCCCGAAGTCC	0.538	37.5	33.33
19568	CCTCGGTCCCGAAGTCCTT	0.587	25.72	25
19569	TCGGTCCCGAAGTCCTTGA	0.613	19.24	58.33
19570	CGGTCCCGAAGTCCTTGAG	0.626	31.47	25
19571	GTCCCGAAGTCCTTGAGCT	0.647	36.96	25
19572	CCCGAAGTCCTTGAGCTCC	0.750	37.5	33.33
19573	CGAAGTCCTTGAGCTCCGA	0.618	17.45	25
19574	GAGCTCCGACTGGTTGTGG	0.808	41.82	33.33

19575	AGCGGGTGAGGCGCTTGCA	0.662	31.92	33.33
19576	GGTGAGGCGCTTGCACTTG	0.688	48.29	41.67
19578	ACTTGCACGAGGCCACCAG	0.525	76.98	58.33
19579	GAGGCCACCAGGCGCACCT	0.712	32.91	33.33
19580	CCAGGCGCACCTTGCGCGC	0.413	48.29	25
19581	CGCACCTTGCGCGCGCGCG	0.606	44.15	25
19582	CACCTTGCGCGCGCGCGGC	0.571	50.45	33.33
19583	GCGCGCGCGGCGCCTCACC	0.260	37.5	16.67
19584	GCGCGCGGCGCCTCACCAC	0.652	37.5	16.67
19585	CGCGGCGCCTCACCACCGG	0.358	37.5	16.67
19586	CGCCTCACCACCGGGACAC	0.629	42.54	33.33
19587	CCACCGGGACACAGCAGCT	0.660	25.72	25
19589	CACGCGCTGCGCGCGGTAG	0.505	35.52	25
19590	ACGCGCTGCGCGCGGTAGC	0.518	54.5	33.33
19592	GTCGGGCCCACTAGGTCGC	0.660	37.77	25
19593	CCACTAGGTCGCCACCACT	0.789	25.72	33.33
19594	ACTAGGTCGCCACCACTTG	0.645	71.94	58.33
19595	CACCACTTGCCGCGGCCGA	0.660	27.88	33.33
19596	GCGGCCGATGGCGTTGGGC	0.643	43.79	16.67
19597	TGGCGTTGGGCAGCAGGCG	0.646	54.5	41.67
19598	TTGGGCAGCAGGCGCGCCG	0.846	72.48	58.33
19599	GCAGGCGCGCCGGGCCGCA	0.521	25.72	25
19600	GGGCCGCACTGGCCGGAGC	0.530	22.57	8.33
19601	CACACCAGCTCGGTGACCG	0.522	66.19	33.33
19602	CTCGGTGACCGGCTTGGCG	0.591	56.74	25
19603	GGTGACCGGCTTGGCGCTG	0.505	33.36	25
19604	GACCGGCTTGGCGCTGCGG	0.513	35.52	16.67
19605	CGGCTTGGCGCTGCGGCAC	0.496	37.5	25
19606	GCTTGGCGCTGCGGCACGG	0.505	40.02	25
19607	GCTGCGGCACGGCCCATCG	0.510	48.29	33.33
19608	CTGCGGCACGGCCCATCGG	0.543	35.52	16.67
19609	CCCATCGGTCACGTAGCGG	0.647	50.45	33.33
19610	CCATCGGTCACGTAGCGGG	0.776	57.19	41.67
19611	CGGTCACGTAGCGGGTGAA	0.575	8.9	25
19612	GGTCACGTAGCGGGTGAAG	0.594	48.29	33.33
19613	GTAGCGGGTGAAGTGCAGC	0.754	67.54	33.33
19614	GGGTGAAGTGCAGCTCGCG	0.548	46.94	25
19615	GGTGAAGTGCAGCTCGCGG	0.668	54.59	33.33
19617	GCAGCTGTACTCGGACACG	0.548	48.29	25
19618	CAGCTGTACTCGGACACGT	0.749	27.88	33.33
19619	AGCTGTACTCGGACACGTC	0.758	63.94	41.67
19620	CTGTACTCGGACACGTCTT	0.871	38.31	41.67
19621	CTTTGGTCTCAAAGGGGTG	0.722	71.67	41.67
19622	GGTGGTGGGGAGGCCGCCC	0.643	48.29	25
19623	GGGAGGCCGCCCTCCGTTC	0.526	29.23	25
19624	TCTCCGCCCGGTTCATGGT	0.695	27.79	41.67
19625	TCCGCCCGGTTCATGGTCT	0.788	23.29	41.67
19626	CCCGGTTCATGGTCTTGTT	0.697	18.71	16.67
19627	CGGTTCATGGTCTTGTTGT	0.522	34.44	50
19628	GTTGTTCTCCAGCTCCGGT	0.833	30.04	33.33
19629	TGTTCTCCAGCTCCGGTGG	0.867	57.01	58.33
19630	TCTCCAGCTCCGGTGGAGG	0.688	71.58	50
19631	GCTCGGGGTACTCTCCGAG	0.444	56.83	33.33
19632	CTCTCCGAGCTCGGGGATG	0.509	57.1	33.33
19633	CCGAGCTCGGGGATGATTT	0.498	27.88	33.33
19634	TCCGTGGCATCATTCTTGA	0.687	42	66.67
19635	TGGCATCATTCTTGAACGC	0.934	43.35	50
19636	CATCATTCTTGAACGCCTG	0.824	65.02	50
19637	CTTGAACGCCTGCCACCCC	0.487	46.31	33.33
19638	AACGCCTGCCACCCCTGGC	0.777	67.45	41.67
19639	CCTGCCACCCCTGGCCCTC	0.711	51.08	25
19640	GCCACCCCTGGCCCTCCAC	0.528	29.23	16.67
19641	CACCCCTGGCCCTCCACTA	0.720	27.88	25
19642	ACCCCTGGCCCTCCACTAC	0.635	54.5	41.67
19643	CCTGGCCCTCCACTACACG	0.607	39.66	16.67
19644	CTGGCCCTCCACTACACGG	0.552	35.52	16.67
19645	CTGACAGCGCCACCGCAGC	0.805	57.1	33.33
19646	CCACCGCAGCACCGTGAGG	0.701	33.36	16.67
19647	ACCGCAGCACCGTGAGGTG	0.563	54.5	33.33
19649	GAGGTGCACGGACTTGGAC	0.557	41.82	25
19650	GTGCACGGACTTGGACGGC	0.661	50.45	33.33
19651	GGACTTGGACGGCTTGCAC	0.754	54.59	33.33
19652	TGGACGGCTTGCACACCAT	0.862	38.58	50
19653	ACGGCTTGCACACCATGCC	0.791	56.74	41.67
19654	GGCTTGCACACCATGCCCT	0.757	15.2	33.33
19655	CGGAGCACGAGCGCTTACT	0.510	26.62	33.33
19656	CACGAGCGCTTACTGAAGC	0.847	41.82	25
19657	GCGCTTACTGAAGCAGCTG	0.565	40.29	25
19658	CGCTTACTGAAGCAGCTGC	0.631	31.47	41.67
19659	CGCGGCCAAAAGCGGCTGC	0.464	24.82	25
19660	AAAAGCGGCTGCCCAGGTC	0.771	84.89	58.33
19661	AAGCGGCTGCCCAGGTCGT	0.511	29.95	33.33

19662	GCGGCTGCCCAGGTCGTTC	0.591	43.79	25
19663	CGGCTGCCCAGGTCGTTCC	0.515	24.82	33.33
19664	CCAGGTCGTTCCACGCGTA	0.518	10.79	16.67
19665	TCGTTCCACGCGTACAGCA	0.852	23.65	50
19666	TCCACGCGTACAGCACGGG	0.603	48.47	50
19667	GCGTACAGCACGGGGCAGA	0.610	26.62	41.67
19668	GTACAGCACGGGGCAGAAT	0.510	23.74	33.33
19669	ACAGCACGGGGCAGAATGT	0.600	27.79	41.67
19670	ACGGGGCAGAATGTCTGCG	0.530	48.11	33.33
19671	CGGGGCAGAATGTCTGCGA	0.476	19.96	25
19672	GGCAGAATGTCTGCGACCA	0.405	24.37	25
19673	CAGAATGTCTGCGACCACA	0.575	38.31	41.67
19674	TGTCTGCGACCACAGCCAC	0.833	50.36	50
19675	CTGCGACCACAGCCACATC	0.817	35.52	16.67
19677	TAACTTCCTCCGCAGCTTC	1.020	67.09	66.67
19678	TCCTCCGCAGCTTCTTGCT	0.977	38.58	50
19679	CGCAGCTTCTTGCTTAGGC	0.857	50.45	25
19680	GCTTCTTGCTTAGGCGCTG	0.626	54.95	33.33
19681	CTTGCTTAGGCGCTGCTTC	0.830	67.54	41.67
19682	TTAGGCGCTGCTTCTTGCC	0.913	78.24	58.33
19683	TAGGCGCTGCTTCTTGCCC	0.933	58.81	41.67
19684	GCTTCTTGCCCTGGGCCAA	0.411	32.37	33.33
19685	CAAGCCCTCGGAGAACTCT	0.551	23.74	33.33
19686	GAACTCTAGCCCTTTGATC	0.783	71.31	50
19687	TCTAGCCCTTTGATCTCGC	0.967	67.09	50
19688	CCCCGACGGCCGCTGCCGC	0.705	28.87	8.33
19689	CCGCTGCCGCAGCAGCTGG	0.783	22.57	25
19690	GCTGCCGCAGCAGCTGGTC	0.552	48.29	25
19691	GCAGCAGCTGGTCCAGCTC	0.718	48.29	25
19692	GCTGGTCCAGCTCCGCCAG	0.587	33.36	16.67
19694	GCGAGGTGGCCATGAAGCC	0.646	44.15	25
19695	TGGCCATGAAGCCTGGGTC	0.826	63.04	50
19696	CCCGAGCAGCGAGCGCAGC	0.671	22.57	16.67
19697	GAGCAGCGAGCGCAGCAGC	0.788	35.52	25
19698	AGCAGCGAGCGCAGCAGCG	0.734	61.15	41.67
19699	CAGCGAGCGCAGCAGCGTC	0.709	50.45	25
19701	GCGCAGCAGCGTCTCGTTC	0.741	28.87	25
19703	GCAGCAGCGTCTCGTTCAG	0.469	48.29	25
19705	GTTCAGATCCTTTTCCTTG	0.951	74.1	58.33
19706	CCTTGGGGTCAAAGATAGG	0.891	58.72	33.33
19708	GGGTCAAAGATAGGGTCTG	0.888	52.97	33.33
19709	GGGTCTGGGTGTTCGATGA	0.678	21.58	33.33
19710	GTCTGGGTGTTCGATGAGG	0.793	57.1	33.33
19711	TCTGGGTGTTCGATGAGGT	1.018	52.79	50
19712	TGTTCGATGAGGTCCACCA	0.964	58.18	66.67
19713	TTCGATGAGGTCCACCAGG	0.817	67.45	58.33
19714	GGGGCAGGTTGTCGCTGGG	0.681	37.5	16.67
19716	TTGTCGCTGGGTGCCGGGC	0.884	59.17	50
19717	TCGCTGGGTGCCGGGCGGA	0.720	31.92	50
19718	CGCTGGGTGCCGGGCGGAT	0.427	21.58	25
19719	GGGTGCCGGGCGGATGTGG	0.514	29.23	25
19720	GCCGGGCGGATGTGGAGAT	0.602	2.25	16.67
19721	GGGCGGATGTGGAGATAGT	0.528	17.72	16.67
19722	GGAGATAGTGCTGGCCGCC	0.874	51.08	33.33
19723	GATAGTGCTGGCCGCCGGC	0.822	67.9	41.67
19724	TTCCAGAGCTCGGCAGCAT	0.845	47.66	58.33
19725	TCCAGAGCTCGGCAGCATG	0.905	61.15	50
19726	AGCTCGGCAGCATGTGTGT	0.780	38.58	50
19727	CTCGGCAGCATGTGTGTTC	0.896	55.49	41.67
19728	TCGGCAGCATGTGTGTTCA	0.865	42	50
19729	ATGTGTGTTCAAGGGACTA	0.761	55.31	58.33
19730	TTCAAGGGACTATCAATGT	0.908	55.31	83.33
19731	TCAAGGGACTATCAATGTT	0.931	53.15	66.67
19732	CTATCAATGTTGGGTTCTC	0.663	74.46	50
19733	ATCAATGTTGGGTTCTCCT	0.942	61.6	66.67
19734	AATGTTGGGTTCTCCTAGA	0.888	59.44	66.67
19735	TGTTGGGTTCTCCTAGAAG	0.861	82.01	66.67
19736	TTGGGTTCTCCTAGAAGGC	0.928	71.22	50
19737	GTTCTCCTAGAAGGCTCTG	0.755	50.09	33.33
19738	TCCTAGAAGGCTCTGGATG	0.786	74.01	58.33
19739	TAGAAGGCTCTGGATGGAG	0.819	77.88	66.67
19742	CTGGATGGAGAGCAGAATG	0.797	54.23	33.33
19744	GGAGAGCAGAATGGTCCTG	0.454	35.61	33.33
19745	AGCAGAATGGTCCTGACAT	0.565	51.44	50
19746	GCAGAATGGTCCTGACATC	0.488	52.07	33.33
19747	GAATGGTCCTGACATCATA	0.321	49.1	50
19748	GGTCCTGACATCATACAGG	0.420	60.61	33.33
19749	CCTGACATCATACAGGGCA	0.508	34.53	41.67
19750	ACATCATACAGGGCAGACC	0.555	77.97	58.33
19751	CATCATACAGGGCAGACCA	0.286	45.23	41.67
19752	TCATACAGGGCAGACCACT	0.611	55.94	66.67
19753	GGCAGACCACTTTTCCTTC	0.633	39.3	25

19754	GACCACTTTTCCTTCAGGA	0.718	37.95	41.67
19755	CACTTTTCCTTCAGGATGT	0.462	38.31	58.33
19756	TTCCTTCAGGATGTCCAGG	0.742	62.59	50
19757	TCCTTCAGGATGTCCAGGC	0.858	66.19	66.67
19758	CCTTCAGGATGTCCAGGCA	0.492	32.37	33.33
19759	AGGATGTCCAGGCATATGT	0.757	44.6	75
19760	GGCATATGTTACCCTGGGT	0.396	25.36	33.33
19761	TTACCCTGGGTGTCCACGT	0.830	55.67	58.33
19762	GGGTGTCCACGTTGGGGTG	0.116	29.23	16.67
19763	GTCCACGTTGGGGTGATAG	0.772	56.74	41.67
19764	TCCACGTTGGGGTGATAGC	0.894	61.15	50
19765	CGTTGGGGTGATAGCAGGG	0.531	54.95	33.33
19766	GTTGGGGTGATAGCAGGGC	0.634	63.49	41.67
19767	TAGCAGGGCGTGAGGAACT	0.844	44.87	58.33
19768	AGCAGGGCGTGAGGAACTT	0.510	38.58	41.67
19769	GCAGGGCGTGAGGAACTTC	0.409	33.36	25
19770	GTGAGGAACTTCACTGTGG	0.743	63.67	41.67
19771	GAGGAACTTCACTGTGGGC	0.467	35.52	25
19772	GGAACTTCACTGTGGGCGC	0.709	54.95	33.33
19773	ACTTCACTGTGGGCGCATT	0.576	38.22	41.67
19774	TTCACTGTGGGCGCATTGT	0.937	55.31	66.67
19775	GTGGGCGCATTGTAAGGGT	0.474	27.88	25
19776	CGCATTGTAAGGGTAGCCA	0.613	33.9	41.67
19777	GCATTGTAAGGGTAGCCAC	0.878	58.72	41.67
19778	CATTGTAAGGGTAGCCACT	0.769	51.89	50
19779	TGTAAGGGTAGCCACTGGG	0.857	74.19	75
19780	GTAAGGGTAGCCACTGGGG	0.677	67.9	41.67
19781	GTAGCCACTGGGGAACTCT	0.870	41.46	41.67
19782	TAGCCACTGGGGAACTCTA	0.672	33.72	41.67
19783	GCCACTGGGGAACTCTAGC	0.690	50.45	25
19784	CCACTGGGGAACTCTAGCG	0.869	50.54	41.67
19785	GAACTCTAGCGAGAGCTTA	0.602	42.45	58.33
19786	TCTAGCGAGAGCTTATACC	0.913	84.26	75
19787	CTAGCGAGAGCTTATACCT	0.924	45.23	41.67
19788	AGCGAGAGCTTATACCTCA	0.806	38.49	58.33
19789	GCGAGAGCTTATACCTCAG	0.758	47.93	25
19790	GAGAGCTTATACCTCAGGT	0.809	44.6	41.67
19791	GAGCTTATACCTCAGGTCT	0.717	34.44	50
19792	AGCTTATACCTCAGGTCTT	0.464	42.36	58.33
19793	CCTCAGGTCTTCATATACT	0.772	35.79	41.67
19794	CTCAGGTCTTCATATACTG	0.822	60.88	41.67
19795	TCAGGTCTTCATATACTGT	0.881	37.86	58.33
19796	AGGTCTTCATATACTGTTC	0.869	64.93	58.33
19797	CTTCATATACTGTTCCAGC	0.904	74.1	50
19798	TTCATATACTGTTCCAGCT	0.975	55.31	75
19799	TATACTGTTCCAGCTGCTC	0.852	88.67	75
19800	ACTGTTCCAGCTGCTCCAT	0.845	31.56	41.67
19801	CCAGCTGCTCCATGGATGG	0.807	48.29	33.33
19802	CAGCTGCTCCATGGATGGT	0.621	12.95	25
19806	TGGGTTGGTCAAAGCCGCG	0.844	54.5	50
19810	CACTCCCACAGAAGTAAAG	0.677	48.2	41.67
19817	AAGCCTTCAGGAGGGTGCG	0.679	67.45	41.67
19819	AACCTGAAGTTGTAGCCTG	0.955	74.01	58.33
19822	AAACGCTGTCGAGCCGGGT	0.941	55.67	50
19823	TCGAGCCGGGTGAAGGAGT	0.802	23.65	41.67
19825	CCCAGGCCTTGTCCAAGTC	0.930	29.23	16.67
19833	TTGACCACGTCCCCTACCC	0.949	76.89	58.33
19834	AGATGGGGCAGAAAGGGTC	0.658	74.19	58.33
19835	TGTCCGGGTGGAAGCGGCA	0.937	42.72	50
19841	AACGGATGCTGTTCTTGAT	0.706	48.65	50
19843	TTGATGAAAATAGTGAAGT	0.929	62.86	75
19846	CATCATGATGGGCGTTTCC	0.748	65.02	50
19847	GTTTCCACTGTGTCCACCT	0.914	51.89	41.67
19848	CCACCTCCGTGGGGCACCA	0.135	10.79	16.67
19852	GCCCGCACTGGCTGTCTGA	0.238	17.72	25
19854	ACACAGCGGTATTTCTCCT	0.718	37.86	50
19855	CACAGCGGTATTTCTCCTC	0.674	63.13	41.67
19856	CCTCACTCTCTGGGCAGAA	0.443	25.72	33.33
19857	TCACTCTCTGGGCAGAATC	0.969	69.06	58.33
19858	TCTCTGGGCAGAATCCTTG	1.018	77.61	75
19859	AGAATCCTTGCATCTGATT	0.792	38.22	58.33
19861	TCAGTAACAATCATCTTGG	0.989	80.4	83.33
19863	ATGACAAAGACCGAGGTGC	0.859	82.91	66.67
19864	GAGGTGCCCTGAGGTGGCG	0.130	35.52	25
19865	GGCGTCACGTAATCAGACA	0.431	21.49	25
19866	GTCACGTAATCAGACACAT	0.339	38.31	41.67
19868	CATGACTCTGTTGGCGTAG	0.534	65.02	50
19870	AATGGCTGTGTCCCGTACC	0.612	78.24	50
19873	TGCAAGAAAACCCACCCTA	0.822	47.39	75
19874	GAGATGATCAGAAGCTGAA	0.468	43.35	58.33
19876	CTGAACTACTCGGTTGATG	0.747	67.18	50
19877	CTCGGTTGATGATCCCGAT	0.393	31.65	25

19878	GGTTGATGATCCCGATGGT	0.251	36.15	33.33
19881	CTCTTCACAACCACCGACT	0.649	43.35	50
19882	CGGTGGACTGCTTCTCCGC	0.818	46.94	25
19883	GACTGCTTCTCCGCTGTGG	0.627	57.1	41.67
19885	GCGATTTTCAGCGTAGTCT	0.771	33.9	58.33
19886	CAGCGTAGTCTCATTCACC	0.873	63.31	25
19887	AACTTCTTGGCTTTGTACT	0.995	61.6	66.67
19888	TTCTTGGCTTTGTACTGGT	0.829	55.31	75
19889	GGCTTTGTACTGGTCGGCC	0.680	50.45	33.33
19891	TTGAGGAAGTTGAGCAGGA	0.903	58.09	66.67
19893	TCCCACAGAAGTAAAGGCC	0.802	63.31	58.33
19894	CCACAGAAGTAAAGGCCGC	0.614	51.08	33.33
19895	GATGATGGTGGGGATGATG	0.385	65.02	41.67
19897	CTTCAGGAGGGTGCGGTAC	0.371	61.24	50
19899	GGGACACGCTGCTTTTCTC	0.683	35.52	16.67
19900	TCTCAGAAACGCTGTCGAG	0.642	71.85	58.33
19901	CAGAAACGCTGTCGAGCCG	0.860	42.18	33.33
19902	AGAAACGCTGTCGAGCCGG	0.980	71.94	58.33
19903	AGGAGTATTTGGGGATGCA	0.563	45.14	50
19904	AAGTCGCACACCCAGCCGA	0.771	38.85	50
19905	CACACCCAGCCGATCTTAA	0.554	29.68	33.33
19907	CCAGCCGATCTTAATGCCC	0.597	48.29	25
19909	TGCGCGCCAGTTTGGCAAA	0.373	17	33.33
19910	TGGCAAAATCCTGCCCCGC	0.678	57.28	50
19911	GCAAAATCCTGCCCCGCAA	0.334	32.37	41.67
19912	GACCACGTCCCCTACCCGC	0.372	50.45	33.33
19913	ACGTCCCCTACCCGCAAGA	0.759	25.9	41.67
19914	CCGCAAGATGGGGCAGAAA	0.352	14.93	25
19916	GGTGGAAGCGGCAGGTCTT	0.399	28.51	25
19917	TCTTCATGTCCCTGGCTGT	0.781	53.15	66.67
19918	TTCATGTCCCTGGCTGTCA	0.905	55.31	75
19919	CATGTCCCTGGCTGTCAGG	0.334	46.31	33.33
19920	TGGCTGTCAGGTTGGGAAG	0.663	54.5	50
19922	TTCTTGATGAAAATAGTGA	0.796	62.86	83.33
19923	TAGTGAAGTTCTCAGCTTC	0.888	80.67	75
19924	CCATCATGATGGGCGTTTC	0.487	58.72	41.67
19926	AGCCCTGGATCTCACAGGT	0.502	31.92	33.33
19927	ACAGGTCCGGAGCACAGAG	0.419	50.36	33.33
19928	GTCCGGAGCACAGAGCTGT	0.592	32.91	41.67
19929	CACAGAGCTGTAGTTCACG	0.420	67.18	33.33
19930	TGTAGTTCACGCAGCGGCC	0.723	71.94	58.33
19931	ACGCAGCGGCCAGTGAGGA	0.164	23.29	33.33
19937	CCTTGCATCTGATTTTCAG	0.855	67.81	41.67
19938	ATCTGATTTTCAGTAACAA	0.541	57.82	50
19939	TCTGATTTTCAGTAACAAT	0.746	42.72	58.33
19940	TGATGATGACAAAGACCGA	0.728	53.15	66.67
19941	CCGAGGTGCCCTGAGGTGG	0.702	44.15	33.33
19942	AGGTGCCCTGAGGTGGCGT	0.086	29.95	33.33
19944	GGTGGCGTCACGTAATCAG	0.500	50.54	33.33
19947	TCCATGACTCTGTTGGCGT	0.892	51.44	58.33
19948	TTGGCGTAGAGTCCGGAGC	0.873	69.69	58.33
19953	GAGCCCTTCACCTTGGTTA	0.162	40.2	41.67
19955	AAAACCCACCCTACAAAGT	0.894	51.17	58.33
19957	GATGATCAGAAGCTGAACT	0.723	36.06	50
19958	ATCAGAAGCTGAACTACTC	0.918	80.67	58.33
19959	GAAGCTGAACTACTCGGTT	0.620	48.74	41.67
19960	GATGATCCCGATGGTCCAG	0.607	46.31	33.33
