# Supplementary material for: Design and evaluation of genome-wide libraries for RNA interference screens
Source: Genome Biol. 2010 Jun 15;11(6):R61. doi: 10.1186/gb-2010-11-6-r61 (PMC2911109; doi:10.1186/gb-2010-11-6-r61)
Supplement: Additional file 3 — NEXT-RNAi summary HTML page for the design of a genome-wide RNAi library for the Drosophila genome. This page provides information about the number of successful designs (here, about 94% of the 74,907 query-sequences could be covered with long dsRNA designs). The 'Links to HTML results' link to detailed reports (Additional file 4) for each design (the full list of links was cut for this figure). 'Links to result files' directly link to NEXT-RNAi output files, such as the tab-delimited result file (the main output file) summarizing all calculations done in one line per design, a FASTA file only containing the final reagent sequences as well as GFF (generic feature file) and AFF (annotation file format) output files for visualization and direct upload of reagents to a genome browser, respectively. Further, links to the user-input text files and to report files (for example, reports about failed designs) are provided. [file gb-2010-11-6-r61-S3.PDF]

NEXT-RNAi results for Dmel_r524 design(s)

Number of queries: 74907
Queries covered by design(s): 70149 (93.65 %)
Queries not coverered by design(s): 4758 (6.35 %)

More statistics on designs are here

Links to HTML results

FBgn0260990_cr7 FBgn0024277_cre5 FBgn0025683_cr5_1 FBgn0260464_cr2_2 FBgn0085196_cr1
FBgn0035648_cr3 FBgn0033062_cr3_2 FBgn0010909_cr17 FBgn0002873_cre5 FBgn0036663_cr5
FBgn0261363_cr3_1 FBgn0036890_cr1 FBgn0015546_cr6_1 FBgn0034703_cr2 FBgn0027582_cr7_2
FBgn0003392_cre4 FBgn0035793_cr5 FBgn0261279_cr5_2 FBgn0259203_cr1 FBgn0034390_cre3
FBgn0032976_cr3_2 FBgn0015320_cr3 FBgn0037677_cr1 FBgn0086442_cr2 FBgn0030098_cr1_1
FBgn0065084_cre2 FBgn0031522_cr6 FBgn0036882_cr2_2 FBgn0033667_cr5 FBgn0038300_cr1_1
FBgn0086046_cre1 FBgn0039543_cr3 FBgn0085395_cre2 FBgn0038295_cr4 FBgn0032166_cr1_2

Links to result files

Tab-delimited result file
Statistics result file
FASTA result file
GFF result file
Annotations result file
Location(s) of mapped reagents
Oligo(s) that could not be mapped
dsRNAs that could not be mapped
Homology of RNAi reagents
miRNA seeds in RNAi reagents

Links to input text files

Database file used for off-target evaluation: dmel-all-txn-miRNA-miscRNA-ncRNA-pseudogene-tRNA-r5.24.fasta
Database file used for mapping of reagents: dmel-all-chromosome-r5.24
Reagent sequence input file (FASTA)
Validated reagent sequence input file (FASTA)
Options input file
Targetgroups input file
Input file for intended targets
File used for calculation of feature contents: dmel-all-UTRs-r5.24.tab
Database file for homology evaluation: dmel-all-txn-miRNA-miscRNA-ncRNA-pseudogene-tRNA-r5.24.fasta

Links to output report files

Error log file
NEXT-RNAi report file
Failed design(s)
