# Supplementary material for: Design and evaluation of genome-wide libraries for RNA interference screens
Source: Genome Biol. 2010 Jun 15;11(6):R61. doi: 10.1186/gb-2010-11-6-r61 (PMC2911109; doi:10.1186/gb-2010-11-6-r61)
Supplement: Additional file 4 — Detailed output for a long dsRNA that targets the Drosophila gene csw (FBgn0000382). The box 'dsRNA information' provides information about the primers (for example, sequence, melting temperature, GC content) required for the synthesis. 'Primer pair penalty' is an overall quality score for the primer pair. The lower this score is, the higher is the predicted quality of the primer pair. Further, the full amplicon sequence, its length and location in the genome (in the format chromosome:start..end(orientation)) are presented. The 'Target information' box shows the intended target(s) and transcript(s) as well as other (unintended) targets and transcripts ('NA' means that no target was found). The intended transcripts are those with most siRNA hits (here, all 203 19-nucleotide siRNAs target the 4 isoforms of csw). The intended gene is then defined over the intended transcripts. The 'Reagent quality' box shows the overall number of siRNAs (here 19-nucleotide siRNAs) contained within the long dsRNA sequence, the number of siRNAs that are 'On-target' (the intended target) and those that are 'Off-target' or have 'No-target'. Further quality features computed for this run were the number of conserved miRNA seeds ('mirSeed') in this dsRNA, the number of 'Efficient siRNAs' (here equal to the overall number of siRNAs, since the efficiency cutoff was set to 0), the 'Average efficiency score' (mean efficiency score of all siRNAs contained in the long dsRNA), and the number of 'Low complexity regions' and 'CAN' repeats contained in the long dsRNA. Additionally, the overlap to UTRs (this long dsRNA completely overlaps with annotated UTRs) and the sequence homology to all transcripts (here only to the intended target) were analyzed in this run. The 'Genome Browser' box visualizes the long dsRNA in its genomic context. [file gb-2010-11-6-r61-S4.PDF]

Query ID: FBgn0000382_cr8_1

Design 1: FBgn0000382_cr8_1_1

dsRNA information

Primer forward

**Sequence**	TAAGTGTTCCCTGCACACGA
**Length [nt]**	20
**Tm[°C]**	60.301
**GC[%]**	50.000

Primer reverse

**Sequence**	AATTCAAGCGGTTTGGAACA
**Length [nt]**	20
**Tm[°C]**	60.481
**GC[%]**	40.000

Primer pair penalty 0.7822

Amplicon sequence

TAAGTGTTCCCTGCACACGAAAAGCAGTAACCCCACCACCACCCCAAAAC
CCAAAACCCAGCATATTTATGTAGTAGTATTGTAAGAATCCCCGAACCGG
TAGTTGAGGATACAATCTGGGCGGAACTGCAGCAGATCCGCCAAAAAACA
AAAACACATTTTTAAGCAGAATCGCAGCAACAAAATATAACACCCGTTCC
CTGTTCCAAACCGCTTGAATT

Amplicon length [nt]

221

Amplicon location

X:2006113..2006333(-)

Target information

**Intended target gene**	FBgn0000382
**Intended target transcripts (hits)**	FBtr0070378 (203), FBtr0070379 (203), FBtr0070380 (203)
**Other targeted gene(s)**	NA
**Other targeted transcripts (hits)**	NA

Reagent quality

siRNAs [19 nt]	On-target	Off-target	No-target	mirSeed	Efficient siRNAs	Avg efficiency score	LowComplexRegions	CAN
203	203	0	0	0	203	54.58	0	0

Additional quality evaluation

**UTR**	221
**Sequence homology (e-value)**	FBgn0000382(1e-122)

Genome Browser

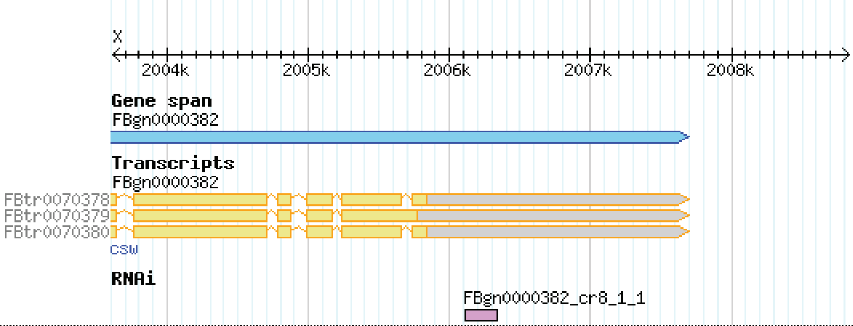

Additional file 4
Horn et al.
