# Supplementary material for: Design and evaluation of genome-wide libraries for RNA interference screens
Source: Genome Biol. 2010 Jun 15;11(6):R61. doi: 10.1186/gb-2010-11-6-r61 (PMC2911109; doi:10.1186/gb-2010-11-6-r61)
Supplement: Additional file 5 — Summary statistics of RNAi reagents designed by NEXT-RNAi for different organisms. NEXT-RNAi was used to design RNAi reagents for all annotated transcripts included in the latest available genome release. CAN = CA[ACGT] repeats; UTR = untranslated region; SNP = single nucleotide polymorphism. [file gb-2010-11-6-r61-S5.PDF]

Additional file 5	NEXT-RNAi designs for different model organisms							
	A. gambiae		***T. castaneum***		***D. melanogaster***		***H. sapiens***	
Annotation database	VectorBase AGAMP3.5		BeetleBase 3.0		FlyBase r5.24		NCBI RefSeq release 40	
Annotated genes	13254		16529		14898		29822	
RNAi reagent type	long dsRNA		long dsRNA		long dsRNA		esiRNA	siRNA
NEXT-RNAi designs	68855		71293		70149		82516	100264
… with CAN repeats (6x)	45	0.1%	29	0.0%	86	0.1%	30	0.0%	8	0.0%
… with low complexity	98	0.1%	43	0.1%	198	0.3%	0	0.0%	0	0.0%
… targeting UTR	-	-	-	-	20513	29.2%	45388	55.0%	42628	42.5%
… targeting SNPs	-	-	-	-	-	-	45901	55.6%	11841	11.8%
… homology to unintended transcripts*	4363	6.3%	5849	8.2%	4438	6.3%	22039	26.7%	29847	29.8%
… 19 nt siRNA 'off-targets'										
0	64702	94.0%	66526	93.3%	65441	93.3%	62929	76.3%	83085	82.9%
1+	4153	6.0%	4767	6.7%	4708	6.7%	19587	23.7%	17179	17.1%
1	416	0.6%	286	0.4%	470	0.7%	546	0.7%	17179	17.1%
2-10	663	1.0%	724	1.0%	568	0.8%	1543	1.9%	-	-
11-50	1316	1.9%	1714	2.4%	865	1.2%	3240	3.9%	-	-
51-100	1434	2.1%	1565	2.2%	2089	3.0%	9146	11.1%	-	-
101+	324	0.5%	478	0.7%	716	1.0%	5112	6.2%	-	-
Gene models covered	12593	95.0%	16423	99.4%	14815	99.4%	29171	97.8%	29805	99.9%
… with no 19 nt 'off-targets'	11822	89.2%	15359	92.9%	13511	90.7%	21996	73.8%	24866	83.4%
… by independent designs	11940	90.1%	13747	83.2%	13210	88.7%	26352	88.4%	28927	97.0%

*E-value < 1e-10 for long dsRNAs, < 0.1 for siRNAs

CAN=CA[ACGT], UTR=untranslated region, SNP=single nucleotide polymorphism
