# Supplementary material for: Design and evaluation of genome-wide libraries for RNA interference screens
Source: Genome Biol. 2010 Jun 15;11(6):R61. doi: 10.1186/gb-2010-11-6-r61 (PMC2911109; doi:10.1186/gb-2010-11-6-r61)
Supplement: Additional file 6 — Summary statistics for Drosophila and human RNAi libraries re-annotated by NEXT-RNAi. CAN = CA[ACGT] repeats; UTR = untranslated region; SNP = single nucleotide polymorphism. [file gb-2010-11-6-r61-S6.PDF]

**Additional file 6**	**NEXT-RNAi evaluation of public / commercial RNAi libaries**																			
	**Ambion**		**HD2**		**HFA/DRSC v1.0**		**DRSC v2.0**		**OpenBiosystems v1,v2**		**MRC**		**NIGFLY**		**VDRC**		**Ambion**		**Qiagen**	
Organism	*D.melanogaster*		*D.melanogaster*		*D.melanogaster*		*D.melanogaster*		*D.melanogaster*		*D.melanogaster*		*D.melanogaster*		*D.melanogaster*		*H.sapiens*		*H.sapiens*	
Annotation database	FlyBase r5.24		FlyBase r5.24		FlyBase r5.24		FlyBase r5.24		FlyBase r5.24		FlyBase r5.24		FlyBase r5.24		FlyBase r5.24		NCBI RefSeq r40		NCBI RefSeq r40	
Annotated genes	14898		14898		14898		14898		14898		14898		14898		14898		29822		29822	
RNAi reagent type	long dsRNA		long dsRNA		long dsRNA		long dsRNA		long dsRNA		long dsRNA		long dsRNA		long dsRNA		siRNA		siRNA	
Number of reagents	13071		19708		21306		23669		15466		13089		9874		15073		64781		70308	
… with CAN repeats (6x)	81	0.6%	90	0.5%	1120	5.3%	424	1.8%	342	2.2%	710	5.4%	422	4.3%	540	3.6%	0	0.0%	0	0.0%
… with low complexity	235	1.8%	152	0.8%	843	4.0%	367	1.6%	369	2.4%	681	5.2%	700	7.1%	196	1.3%	0	0.0%	0	0.0%
… targeting UTR	3124	23.9%	4478	22.7%	1074	5.0%	3364	14.2%	4033	26.1%	905	6.9%	667	6.8%	945	6.3%	6292	9.7%	24316	34.6%
… targeting SNPs	-	-	-	-	-	-	-	-	-	-	-	-	-	-	-	-	5897	9.1%	6350	9.0%
… homology to unintended transcripts**	700	5.4%	1813	9.2%	1519	7.1%	1736	7.3%	1354	8.8%	1494	11.4%	983	10.0%	1784	11.8%	5322	8.2%	12912	18.4%
… miRNA-seeds / dsRNA (average)	1.9		2.7		2.9		2.9		3.5		4.4		5.5		2.3		-		-	
… 19 nt siRNA 'off-targets'																				
0	9197	70.4%	13110	66.5%	9165	43.0%	15044	63.6%	8729	56.4%	6296	48.1%	4452	45.1%	8861	58.8%	54536	84.2%	64924	92.3%
1+	3251	24.9%	5248	26.6%	7903	37.1%	7361	31.1%	5435	35.1%	6736	51.5%	5378	54.5%	6126	40.6%	3698	5.7%	2864	4.1%
1	1376	10.5%	2093	10.6%	2120	10.0%	2214	9.4%	2082	13.5%	1767	13.5%	1439	14.6%	1709	11.3%	3698	5.7%	2864	4.1%
2-10	1399	10.7%	1800	9.1%	3579	16.8%	3446	14.6%	1976	12.8%	3023	23.1%	2622	26.6%	2641	17.5%	-	-	-	-
11-50	113	0.9%	285	1.4%	1402	6.6%	784	3.3%	667	4.3%	1130	8.6%	849	8.6%	898	6.0%	-	-	-	-
51-100	68	0.5%	417	2.1%	217	1.0%	124	0.5%	143	0.9%	207	1.6%	123	1.2%	128	0.8%	-	-	-	-
101+	295	2.3%	653	3.3%	585	2.7%	793	3.4%	567	3.7%	609	4.7%	345	3.5%	750	5.0%	-	-	-	-
… no transcript target found	623	4.8%	1350	6.9%	4238	19.9%	1264	5.3%	1302	8.4%	57	0.4%	10	0.1%	86	0.6%	6521	10.1%	2406	3.4%
Gene models covered	11906	79.9%	14587	97.9%	13226	88.8%	13799	92.6%	12960	87.0%	11747	78.8%	5312	35.7%	12948	86.9%	22379	75.0%	19479	65.3%
… all transcripts covered	11514	77.3%	14485	97.2%	12980	87.1%	13675	91.8%	12562	84.3%	11506	77.2%	5170	34.7%	12746	85.6%	22129	74.2%	19210	64.4%

* The HFA library was designed for the Heidelberg Collection of genes (HDC, Hild et al. 2003), which contains ~7000 genes not covered by FlyBase annotations

**E-value < 1e-10 for long dsRNAs, < 0.1 for siRNAs

CAN=CA[ACGT], UTR=untranslated region, SNP=single nucleotide polymorphism
