# Supplementary material for: Design and evaluation of genome-wide libraries for RNA interference screens
Source: Genome Biol. 2010 Jun 15;11(6):R61. doi: 10.1186/gb-2010-11-6-r61 (PMC2911109; doi:10.1186/gb-2010-11-6-r61)
Supplement: Additional file 7 — Raw data for comparison of Drosophila RNAi libraries in Figure 4, including number of genes targeted by each library, number of genes targeted by both the compared libraries and number of genes targeted with independent designs (with no sequence-overlap at all). [file gb-2010-11-6-r61-S7.PDF]

Additional file 7 **Comparison of eight *Drosophila* RNAi libaries**

Library	Number of genes targeted (FlyBase r5.24)
Ambion (AMB)	11906
DRSC version 2 (DRSCv2)	13799
Heidelberg 2 (HD2)	14587
Heidelberg Fly Array / DRSC version 1 (HFA)	13226
Cyclacel (MRC)	11747
NIG-FLY (NIGFLY)	5312
Open Biosystems version 1,2 (OBS)	12960
Vienna GD library (VDRC)	12948

Library comparison

Library 1 vs Library 2	Number of genes targets by both libraries	Number of genes targeted by independent designs
AMB-DRSCv2	11438	6515
AMB-HD2	11431	6203
AMB-HFA	11043	5243
AMB-MRC	10202	4546
AMB-NIGFLY	4751	2283
AMB-OBS	11117	5076
AMB-VDRC	10945	5786
DRSCv2-HD2	13197	6623
DRSCv2-HFA	12546	3814
DRSCv2-MRC	11321	4146
DRSCv2-NIGFLY	5188	2468
DRSCv2-OBS	12511	5123
DRSCv2-VDRC	12146	5474
HD2-HFA	12504	5361
HD2-MRC	11307	4429
HD2-NIGFLY	5188	2351
HD2-OBS	12505	5066
HD2-VDRC	12137	5498
HFA-MRC	11237	2593
HFA-NIGFLY	5100	2003
HFA-OBS	12069	3786
HFA-VDRC	11759	3849
MRC-NIGFLY	4873	1730
MRC-OBS	11102	2632
MRC-VDRC	10958	3161
NIGFLY-OBS	5099	1992
NIGFLY-VDRC	5071	2055
OBS-VDRC	11891	3923
