# Supplementary material for: Design and evaluation of genome-wide libraries for RNA interference screens
Source: Genome Biol. 2010 Jun 15;11(6):R61. doi: 10.1186/gb-2010-11-6-r61 (PMC2911109; doi:10.1186/gb-2010-11-6-r61)
Supplement: Additional file 8 — Primer sequences and target gene information for the independent long dsRNAs designed against 49 Drosophila phosphatases for the knock-down validation study presented in Figure 5. [file gb-2010-11-6-r61-S8.PDF]

Additional file 8

PCR and quantitative RT-PCR primer as well as annotations for RNAi reagents targeting *Drosophila* phosphatases

DesignID	IndependentDesign	Length[nt]	RNAiPrimerFor	RNAiPrimerRev	qPCRPrimerFor	qPCRPrimerRev	FBgnID	AnnotationSymbol	Name	Symbol
SGI00042	1	175	ACGGCGACCTGTACAACAT	GCACAGTTGCTATACGGCAG	CATTCAACGCCACACGAATCACG	TTCCTCCCAGAAACCTCCCTTG	FBgn0000382	CG3954	corkscrew	csw
SGI00043	1	171	CAAACGAAAACCGTTGGACT	GTGAGCGCGAAGATTTAAGG	TGCACTACGAGCTCTACTGGAATG	CTCCGAGTTGGAAATGCGCTTG	FBgn0000464	CG10443	Leukocyte-antigen-related-like	Lar
SGI00045	1	166	TGCCCATCGATGAAATAACA	AGGCGCGAGATGATACTGTC	GCTTCACATTTGGAGCAGAGGTTG	AGCCCGACAGATCAGGTCAAATTC	FBgn0003134	CG6593	Protein phosphatase 1alpha at 96A	Pp1alpha-96A
SGI00046	1	175	ACCCCTCATTAGCAACGACA	GCTCAGCTCTTTGCCGATAC	TGCAATGTGTCCAAGGTGCTCTG	GGTTCTTCAGCGTCAAGAAAGGTG	FBgn0003138	CG9181	Protein tyrosine phosphatase 61F	Ptp61F
SGI00047	1	175	GATACCGTACAAGGGTGCCT	CTCGTTGACCAGCTGATGTG	TTTGCTTACCATCGCTGCCATC	TTGTACGGTATCTCGCCGTTGC	FBgn0003139	CG12217	Protein phosphatase V	PpV
SGI00048	1	175	ATGGAGCTCTTCGAGATGGA	TGTTGCTCTCCGTCATGCT	ATGTCCCGCTTCCTGCGTAATC	AGATCTCGGGATAGTGCAACGC	FBgn0003525	CG1395	string	stg
SGI00049	1	175	CTCCAAGGAGTCCAATGTGC	CACGGTCTCCACGGAGTAGT	CACAACTGCGATGGAGGATAAAGC	TGGAGAGAATCTCCTTGGCCTTG	FBgn0004177	CG7109	microtubule star	mts
SGI00050	1	175	ATCGATCAGGGTCTCATGCT	CGTGCACCTTCACCTCATAA	CGTGTTTGCCATGCGAAAGGAG	AGCAGACACTGATGGATGCACAC	FBgn0004368	CG6899	Protein tyrosine phosphatase 4E	Ptp4E
SGI00051	1	175	GTACGCGAAGCTGTACCACA	TTTGCTATAGAGATCCGGCG	TGACTACAACCTGGCCGTTCTG	CGCATGGAAGCACTTTCTGCTC	FBgn0004369	CG11516	Protein tyrosine phosphatase 99A	Ptp99A
SGI00052	1	175	AAGGGCGTACAACATATCCG	AAATTCGGATATCCCCTTGG	CTAGTCGCAACTTCACCACAAAGC	ACCAGCAGTGTCGTCTCATTACG	FBgn0004370	CG1817	Protein tyrosine phosphatase 10D	Ptp10D
SGI00053	1	175	AGCGCGATATATTACGCCAA	AGTCACAAAGGGCTTGCTTG	CGCGGCAACCATGAAAGCATTAAC	TGGTGTACTTCGCTGTCACCTC	FBgn0005777	CG8402	Protein phosphatase D3	PpD3
SGI00054	1	175	CTCAAGTGGCTTAAGGACGG	TGGAGGTAATTTCGAGTCCG	GGAAACCCAACGAGGGTAAATGC	TGCTGTGGCCAGTAGTTTCTCG	FBgn0014007	CG10975	Protein tyrosine phosphatase 69D	Ptp69D
SGI00055	1	175	CTACCTGCAGGCTCGCAT	CACGACCAGTGCGCTTATG	TGGTCGTGGCCAACTATGTCTC	TGCACTGTACGCGCCAAATAGG	FBgn0016078	CG8804	wunen	wun
SGI00056	1	172	GAACACGCAGCGCTACATAA	CTCATTCAATTCGATGGGGA	TACATCAAGGGACCCGACTACGTG	CTCGAAGATTGTATTGGGCAGTGG	FBgn0016641	CG9856	Protein tyrosine phosphatase-ERK/Enhancer of Ras1	PTP-ER
SGI00057	1	175	GAAGACGAGAATCCAGCGTT	GTAGCTCCAGAGATCGCCC	TCTGGTCTGATCAGGATGAGGATG	CAGTGCGTGGCCATTTCTCTTG	FBgn0022768	CG2984	Protein phosphatase 2C	Pp2C1
SGI00058	1	173	ACAGCAAGAGCTCGATCACC	GAAGTTAAGCACCGTCTCGG	TCAACAAGGTGTTCGCAATCGAC	TGATCGAGCTCTTGCTGTCCTG	FBgn0023508	CG3573	-	CG3573
SGI00059	1	159	GAGCTAAAGAAGAACAATGTCAACAC	TAAGACCTCAAACCACTCGTCA	GTTGCCTTGGCACTGATTGAATTG	ATGGCTCCGCGTCGTTTATC	FBgn0024734	CG4993	PRL-1	PRL-1
SGI00060	1	175	TGAATCCACTCTTTCCCTCG	AGCTGGTCCTGCTTGGATAG	GGCCGAAGATCTCAAGCACAAGAC	AATACTGGTCCAGCGACGAGTTG	FBgn0025742	CG9115	myotubularin	mtm
SGI00061	1	164	TTTCAATCGTTTTTGAGGCA	ATCATTGACGATCGCAGTTAAG	AGGGCCTATTCAGAAACCGTCTG	CGAACGGATAAACAGCTACTCTCC	FBgn0026379	CG5671	Pten	Pten
SGI00062	1	175	GCACATGAAAATCCTGTCCA	GCTGCGACTCCACACATCTA	CAAGAGAGCGACCAACATTGCG	TTGGCCACAATCAGCTTTGAGC	FBgn0027515	CG7115	-	CG7115
SGI00063	1	175	ATCACCCTGGACATTTGCAT	CCGTCTGTAGCATTCCACG	TTCCTGCAGAAGGTGCGTGAGAAG	CCAGGGTGATGAATGTACCTGTGC	FBgn0028341	CG32697	lethal (1) G0232	l(1)G0232
SGI00064	1	175	GCATGTACAGTCGCTTCGAG	GTGGCGTCTTGCAGTTTCTT	TGAAACTCTTCCAGCCGGTGTC	TGTTCGGCTGGTAGTTGAAGCAG	FBgn0028497	CG3530	-	CG3530
SGI00065	1	175	GCGTGTACGACGATGAGAAG	TGGAACTCCCACTGGTAAGC	TCCAGCTCCACCTGCAATCAATG	TGTGACTCCAACTTGACAGCCATC	FBgn0029157	CG6238	slingshot	ssh
SGI00066	1	162	GCACCGACTATGGGATTGAG	CAGTGACCTCCGTTTGATCC	ATGAACGTGGCCCTTTCTGGAG	AGCTTCTTTGAGTGCCACTGCTG	FBgn0029958	CG12151	Pyruvate dehydrogenase phosphatase	Pdp
SGI00067	1	174	GGACTTCTGGGACATTTGGA	CGAAAGTCCACCGAGTTGAG	TGCACCTTTCAACCCGAGATGC	CCAGAGGCCCAGTTTCACCTTTAG	FBgn0030556	CG1810	mRNA-capping-enzyme	mRNA-capping-enzyme
SGI00068	1	173	CAAAGATGCAAATGCGAATG	AGCTCGAGCCGAAGGTATTT	AAGACAGAGCGGACACAATGGC	TGGATTCAACGGTCAGGTTGGG	FBgn0030735	CG3632	-	CG3632
SGI00069	1	173	CCGAGCCGTAAACTCACCT	ATGTCGATCATCGTCTTTTCC	CCAAGCGCAGCACAATATCGAC	AGTTTACGGCTCGGCGGAAATG	FBgn0030758	CG9819	Calcineurin A at 14F	CanA-14F
SGI00071	1	170	CAGTCGCAGCTCATCCACT	AGTTGGGGTCAATCTTGCAG	AGGGCTGCGTTGACTTTATCAGC	TAGCAGTGCACAAGGACATTGCC	FBgn0031044	CG14211	MAPK Phosphatase 4	MKP-4
SGI00072	1	175	ATCCCGGTTTGCCAGTATTA	CTTCGCCTTCCTCTTCCTCT	GATACGCCGAATGCGTCAACTC	AAAGAACTTGCCGGCAGAAGGC	FBgn0031194	CG17598	-	CG17598
SGI00073	1	175	TGACTCGCAGCTATTCATGG	AACGTAACCATGCCGATTGT	AGAAGTTCCGCCGGGACATAAC	AACTCGAGGTATATCCAGGTCCTC	FBgn0031799	CG9493	Pez	Pez
SGI00074	1	175	CGGGTAGATACGATGCACCT	TGATCGTGTCCGTATCGTTG	TCAGCAGCAGTGGATGGAAGAC	TCAGCTCCAGGCTGTTGATACC	FBgn0031952	CG7134	cdc14	cdc14
SGI00075	1	174	CACTCTGGCTCTTTATCCGC	GCTGGTGATCTTCTTCTGGG	ACTCCATCGAAGCACACAGCAG	ACGGTGACTTCACACTCCTTGG	FBgn0032702	CG10376	-	CG10376
SGI00076	1	175	ACTGCCGTAATCGTCCAGTT	GAATTTCGTCGTCAGCATCA	ACTTCATCCGCAGAAAGCTCTGAC	TTACCATCTTCGTCACCGTCGTC	FBgn0033021	CG10417	-	CG10417
SGI00077	1	175	ATCCCGCACATGAAAGAAAT	GATAGTCTTCGATGCGCTCC	TTTGCTGATGCTGCCAAATCCG	ACGTCACAAGGTGGCTGTTGATG	FBgn0034179	CG6805	-	CG6805
SGI00078	1	175	ATCATTTCCCGACTGAGCTG	CTGGCTGTTCCCAGAAGAGT	TACTGGACAACTCAAAGCAGGAAG	GCGCTTGCATAGCTCTCTCAAC	FBgn0034691	CG6562	synaptojanin	synj
SGI00079	1	175	TACCAGTACGTCCCGGAGAG	GAGCCTCAGTAATGGCCAGA	TGCCAGCTACACTGTGCAATCC	TAGCCCGGCTGGTGTTCTAATG	FBgn0035133	CG1228	Ptpmeg	Ptpmeg
SGI00080	1	175	AAGTGCTCGAGTTCTGTCGC	ATCAGATCGCTGTAGGGACG	ACCGGAAGATCAAATCGTCACAGC	TCCGCGTTGCTCATTACATCCC	FBgn0035425	CG17746	-	CG17746
SGI00083	1	175	AGCTTTATTTGCAACCGAGC	CGAGCTGTAAAAGTGCTCCC	AAAGCTGGACAAGCAGACCGATG	ACAGCCGGCACTCAAGTTCTTC	FBgn0036448	CG9311	myopic	mop
SGI00084	1	173	CTCTGATGCAGGAGGGCTAT	CTGTGAAACTGCCGACTCCT	AGCCTATCTGAATGGCGAACCC	CAATCACTGCTTGCTTGATCGTC	FBgn0036551	CG17029	-	CG17029
SGI00085	1	152	TGTCCTTCGAGAGCCAACTG	GAACTCGATGCCGGAGTCT	TCAGACGCACAGCAAAGAGATCG	TGGAAATGCGCAGGGACCTAAG	FBgn0036844	CG14080	Mitogen-activated protein kinase phosphatase 3	Mkp3
SGI00086	1	175	GAATTCACGCCTGGGATGT	GGAAGGGGATTAAACTGCGT	CCACTTTCCGGAGCACAAGTTC	AGATGCAAGAGTGCGGAAAGGC	FBgn0037063	CG9391	-	CG9391
SGI00087	1	175	AATGACTGCGAGCAAGGAAG	TCGTTCCTCTCGCTTTTCTG	TTCCGGGCGACATAAACGAAGG	ACCGAAACTCGCAAAGGCGAAC	FBgn0037341	CG12746	-	CG12746
SGI00088	1	175	CTTCGAGCTCAGCCGTTAGT	CAGGACATTGTACAGCAGGG	CGTGGGTTCTGTCTATGTGCACTG	TCCGGAGTCCATCCGTTCTTCATC	FBgn0039111	CG10371	PTEN-like phosphatase	Plip
SGI00091	1	175	ATCATCCGACCAACAAGAGC	GGTGACGTGGTGAAGTGATG	ACCGGACTTTCGGTACTCCATC	TTGATGAGGCCAGGATCCACTG	FBgn0043903	CG14226	domeless	dome
SGI00092	1	175	TGAAGGAGAATCTTCGGCTG	AGCTTATGCGAGGGTGTCTG	GGTCAAGGACATACCAGACAGATG	CAGCGGCACCTTAAAGGCTATG	FBgn0062449	CG13197	-	CG13197
SGI00093	1	167	GGATTCCTGGATAAGCCGA	ACGGCAAAGAAGCTCCAGT	GTGGATCTGCTGAAGTGTCTGC	GTGCGCTCGATAACGTGATATTTG	FBgn0086361	CG1906	alphabet	alph
SGI00094	1	175	TATAGAAACACACCCCGCCT	CGTATCGCTGGCAGGTATTT	ATAAAGCGAGAAGCGCCGTGTC	TGGCACCAATGCGATTGTTGTTG	FBgn0243512	CG7850	puckered	puc
SGI00095	1	175	GCTGGAGCCACTTAAAGACG	CCAATCATCGCTCTGGATAA	CGACTTCACCTGCGTACACATC	ACTTCCAAGGGCCGTAAAGTGC	FBgn0259178	CG42283	5PtaseI	5PtaseI
SGI00096	1	175	CCACTCGATCTTCCGATCAT	AAGGAAACTGCAGTGGTTGG	TGTGAGGACAAGAACCGCTACG	TTTGTCATCGTCGCCTTGCC	FBgn0259227	CG42327	-	CG42327
SGI00138	2	172	GACATCCAGCGCACCATT	TTATATTGGTGTACTCGCGGC	CATTCAACGCCACACGAATCACG	TTCCTCCCAGAAACCTCCCTTG	FBgn0000382	CG3954	corkscrew	csw
SGI00139	2	175	ATCGACTTCATCGGACAGGT	GTATGCGCACTGTCTGGAAG	TGCACTACGAGCTCTACTGGAATG	CTCCGAGTTGGAAATGCGCTTG	FBgn0000464	CG10443	Leukocyte-antigen-related-like	Lar
SGI00141	2	161	TGTCCGTGGACGACACATT	GTTCGAGGTGTGCAGTGTTT	GCTTCACATTTGGAGCAGAGGTTG	AGCCCGACAGATCAGGTCAAATTC	FBgn0003134	CG6593	Protein phosphatase 1alpha at 96A	Pp1alpha-96A
SGI00142	2	175	ATGGGAGCAGAAGTCCAGAG	CCGCACAAAGTTCTGGTAGG	TGCAATGTGTCCAAGGTGCTCTG	GGTTCTTCAGCGTCAAGAAAGGTG	FBgn0003138	CG9181	Protein tyrosine phosphatase 61F	Ptp61F
SGI00143	2	161	GCGACGTGGACAAGTGGATA	CTGGCCATGGATGTCACC	TTTGCTTACCATCGCTGCCATC	TTGTACGGTATCTCGCCGTTGC	FBgn0003139	CG12217	Protein phosphatase V	PpV
SGI00144	2	175	GGAACTGATGAGCATGGACC	TGATACTCCCATAGCTGGCA	ATGTCCCGCTTCCTGCGTAATC	AGATCTCGGGATAGTGCAACGC	FBgn0003525	CG1395	string	stg
SGI00145	2	162	TTCGGAAACCTTTAACAACACA	AGTTCCATAAGAGCCGCTTG	CACAACTGCGATGGAGGATAAAGC	TGGAGAGAATCTCCTTGGCCTTG	FBgn0004177	CG7109	microtubule star	mts
SGI00146	2	175	AGTCGATTACTTCCAGCCCA	CTTTCCCTGGGTAGCATTCA	CGTGTTTGCCATGCGAAAGGAG	AGCAGACACTGATGGATGCACAC	FBgn0004368	CG6899	Protein tyrosine phosphatase 4E	Ptp4E
SGI00147	2	175	ACCCTACACCGACTACCGTG	CTCATAGGGTCTTCGCCATC	TGACTACAACCTGGCCGTTCTG	CGCATGGAAGCACTTTCTGCTC	FBgn0004369	CG11516	Protein tyrosine phosphatase 99A	Ptp99A
SGI00148	2	175	ATCCGCTAGCTTTACGACCA	TGCGTCCGTGAGATTATTGA	CTAGTCGCAACTTCACCACAAAGC	ACCAGCAGTGTCGTCTCATTACG	FBgn0004370	CG1817	Protein tyrosine phosphatase 10D	Ptp10D
SGI00149	2	174	TATGTGCGAGTTGCTGTGGT	TGAGCCACTTCATAGCCCAT	CGCGGCAACCATGAAAGCATTAAC	TGGTGTACTTCGCTGTCACCTC	FBgn0005777	CG8402	Protein phosphatase D3	PpD3
SGI00150	2	173	AACGTGGAAGGACTTCATGG	CCTCCAGCTGTTGGATTAGG	GGAAACCCAACGAGGGTAAATGC	TGCTGTGGCCAGTAGTTTCTCG	FBgn0014007	CG10975	Protein tyrosine phosphatase 69D	Ptp69D
SGI00151	2	175	CATTTCACAGAACAAGGCCA	GCCGATGGAGTACTTGGCTA	TGGTCGTGGCCAACTATGTCTC	TGCACTGTACGCGCCAAATAGG	FBgn0016078	CG8804	wunen	wun
SGI00152	2	175	CCAGCCATCGACAAGTGTTA	GCGGTGGTACATAAGCCAGT	TACATCAAGGGACCCGACTACGTG	CTCGAAGATTGTATTGGGCAGTGG	FBgn0016641	CG9856	Protein tyrosine phosphatase-ERK/Enhancer of Ras1	PTP-ER
SGI00153	2	175	GTTGTTGTTGTTGAACCGGC	TTAAAGGCTTGGCTGGTGAT	TCTGGTCTGATCAGGATGAGGATG	CAGTGCGTGGCCATTTCTCTTG	FBgn0022768	CG2984	Protein phosphatase 2C	Pp2C1
SGI00154	2	175	ATCATGGAGATTCGGCAGAG	AATCGATGACCGTCTTCTCG	TCAACAAGGTGTTCGCAATCGAC	TGATCGAGCTCTTGCTGTCCTG	FBgn0023508	CG3573	-	CG3573
SGI00155	2	172	ACAGCAGCGGCATCAATTAG	CAGTTCCCGTTTTGTTTTCG	GTTGCCTTGGCACTGATTGAATTG	ATGGCTCCGCGTCGTTTATC	FBgn0024734	CG4993	PRL-1	PRL-1
SGI00156	2	175	ACAGCAGCTCCAAGTCCAGT	TTGGTTATGGTAAGAGCGCC	GGCCGAAGATCTCAAGCACAAGAC	AATACTGGTCCAGCGACGAGTTG	FBgn0025742	CG9115	myotubularin	mtm
SGI00157	2	174	CTGCGCATATTTAGTGTTCAGTG	AGGCTTACTTTCGAATATGGAACA	AGGGCCTATTCAGAAACCGTCTG	CGAACGGATAAACAGCTACTCTCC	FBgn0026379	CG5671	Pten	Pten
SGI00158	2	175	GTCCAAGCTTCTGAAGACGG	AATAGCACTACAATCCGCCG	CAAGAGAGCGACCAACATTGCG	TTGGCCACAATCAGCTTTGAGC	FBgn0027515	CG7115	-	CG7115
SGI00159	2	171	GGGCGATAATAATACCACCGT	CTGCTACTGCTGTTATTCGCC	TTCCTGCAGAAGGTGCGTGAGAAG	CCAGGGTGATGAATGTACCTGTGC	FBgn0028341	CG32697	lethal (1) G0232	l(1)G0232
SGI00160	2	174	AACTACCAGCCGAACAAGGA	CTCCTTGGGCACGTAAATCT	TGAAACTCTTCCAGCCGGTGTC	TGTTCGGCTGGTAGTTGAAGCAG	FBgn0028497	CG3530	-	CG3530

SGI00161	2	173	AAAGTGTCGAAAAAGGCTCG	TTGTTTCGTATGGCATCAGG	TCCAGCTCCACCTGCAATCAATG	TGTGACTCCAACTTGACAGCCATC	FBgn0029157	CG6238	slingshot	ssh
SGI00162	2	175	AGCCAGTCGTTCCTCAAGTG	CCTGCGAGATCTCTTCATCC	ATGAACGTGGCCCTTTCTGGAG	AGCTTCTTTGAGTGCCACTGCTG	FBgn0029958	CG12151	Pyruvate dehydrogenase phosphatase	Pdp
SGI00163	2	172	GAACATAAAGCGGCTAAGCG	ATGCTCGTTTAGGTTCCTGC	TGCACCTTTCAACCCGAGATGC	CCAGAGGCCCAGTTTCACCTTTAG	FBgn0030556	CG1810	mRNA-capping-enzyme	mRNA-capping-enzyme
SGI00164	2	174	AGGTGCGAGATCTGTTCCAG	CGCAGGTCCAGGAGTAGAAG	AAGACAGAGCGGACACAATGGC	TGGATTCAACGGTCAGGTTGGG	FBgn0030735	CG3632	-	CG3632
SGI00165	2	175	CAAAAGGCCAATGTCAACAA	ATATTGTGCTGCGCTTGGTT	CCAAGCGCAGCACAATATCGAC	AGTTTACGGCTCGGCGGAAATG	FBgn0030758	CG9819	Calcineurin A at 14F	CanA-14F
SGI00167	2	171	CCGTCCGGATCCAGATATAA	AGCTTAGCTGCAGCGACTTC	AGGGCTGCGTTGACTTTATCAGC	TAGCAGTGCACAAGGACATTGCC	FBgn0031044	CG14211	MAPK Phosphatase 4	MKP-4
SGI00168	2	166	AAGGAGCGTATTATTTCGGC	GGGCATAGGGGAACTTGTC	GATACGCCGAATGCGTCAACTC	AAAGAACTTGCCGGCAGAAGGC	FBgn0031194	CG17598	-	CG17598
SGI00169	2	175	AGAATGTACCCCTGCCACAG	TCGGGTGGAGAATTGACAGT	AGAAGTTCCGCCGGGACATAAC	AACTCGAGGTATATCCAGGTCCTC	FBgn0031799	CG9493	Pez	Pez
SGI00170	2	173	CAACACCCACTACTTCAGCG	CCGGATTCATGGAGGTGTAG	TCAGCAGCAGTGGATGGAAGAC	TCAGCTCCAGGCTGTTGATACC	FBgn0031952	CG7134	cdc14	cdc14
SGI00171	2	175	CCTGATGGAATTCAAGCGAT	TCGAGTCTCCAGTTGTTTCCA	ACTCCATCGAAGCACACAGCAG	ACGGTGACTTCACACTCCTTGG	FBgn0032702	CG10376	-	CG10376
SGI00172	2	174	TAATAAAGGCAGGTGGTCGG	ACTCGTCCTCTGGCGTGATA	ACTTCATCCGCAGAAAGCTCTGAC	TTACCATCTTCGTCACCGTCGTC	FBgn0033021	CG10417	-	CG10417
SGI00173	2	175	CAGAGCAACATTTATCCGGG	CAGATCCGTGCGTCATTTC	TTTGCTGATGCTGCCAAATCCG	ACGTCACAAGGTGGCTGTTGATG	FBgn0034179	CG6805	-	CG6805
SGI00174	2	175	GAAAGGAACGCTGACTACGC	ACTCGAGGACGGTTGAGAGA	TACTGGACAACTCAAAGCAGGAAG	GCGCTTGCATAGCTCTCTCAAC	FBgn0034691	CG6562	synaptojanin	synj
SGI00175	2	175	CACAGGCGACTACATCAACG	ACCAGACTCCATTACGGTCG	TGCCAGCTACACTGTGCAATCC	TAGCCCGGCTGGTGTTCTAATG	FBgn0035133	CG1228	Ptpmeg	Ptpmeg
SGI00176	2	174	CGACTACGTTATGCTACGCAA	TTATTGGGCTTGTGGTCCAG	ACCGGAAGATCAAATCGTCACAGC	TCCGCGTTGCTCATTACATCCC	FBgn0035425	CG17746	-	CG17746
SGI00179	2	175	CTGCTGGTCTTTCAGCAACA	CCACTGGACCATCATCTCCT	AAAGCTGGACAAGCAGACCGATG	ACAGCCGGCACTCAAGTTCTTC	FBgn0036448	CG9311	myopic	mop
SGI00180	2	175	AGGGACAAGAATGTGAAGCG	CTTCGGTCAGAATGATGGCT	AGCCTATCTGAATGGCGAACCC	CAATCACTGCTTGCTTGATCGTC	FBgn0036551	CG17029	-	CG17029
SGI00181	2	169	GTGACGCTCGCCTACTTGAT	AGCTGCACGAGAACCTGG	TCAGACGCACAGCAAAGAGATCG	TGGAAATGCGCAGGGACCTAAG	FBgn0036844	CG14080	Mitogen-activated protein kinase phosphatase 3	Mkp3
SGI00182	2	175	GCTGGGCTTGGTCTACAATC	ATGCACGACCTTCATCTTGG	CCACTTTCCGGAGCACAAGTTC	AGATGCAAGAGTGCGGAAAGGC	FBgn0037063	CG9391	-	CG9391
SGI00183	2	175	GGGTGATAGTGGCTCCCTTT	CAGCGGTAAAAATAGTCGGG	TTCCGGGCGACATAAACGAAGG	ACCGAAACTCGCAAAGGCGAAC	FBgn0037341	CG12746	-	CG12746
SGI00184	2	175	GAGGACTATGAGCTGACCGC	GGCCACCAATTCTTTGCTT	CGTGGGTTCTGTCTATGTGCACTG	TCCGGAGTCCATCCGTTCTTCATC	FBgn0039111	CG10371	PTEN-like phosphatase	Plip
SGI00187	2	175	ATTCCAAGCACATCCGAATC	TCGTAGTCCAGGCAGTTGAA	ACCGGACTTTCGGTACTCCATC	TTGATGAGGCCAGGATCCACTG	FBgn0043903	CG14226	domeless	dome
SGI00188	2	164	ATGAAATCGAACGCGACAAT	GTGGTTGTTTTTCTGATGCAAA	GGTCAAGGACATACCAGACAGATG	CAGCGGCACCTTAAAGGCTATG	FBgn0062449	CG13197	-	CG13197
SGI00189	2	175	ATCTATAATGCGGGTGGCAG	GGAACTCATCGCTATCCTGG	GTGGATCTGCTGAAGTGTCTGC	GTGCGCTCGATAACGTGATATTTG	FBgn0086361	CG1906	alphabet	alph
SGI00190	2	175	AAGATGCACGGAAAACGG	CCCATGAAGTTCAGATTGGG	ATAAAGCGAGAAGCGCCGTGTC	TGGCACCAATGCGATTGTTGTTG	FBgn0243512	CG7850	puckered	puc
SGI00191	2	162	TGTAAATGGTCCCGAAAAGG	ATGAACTAGGGCTCTTCTGCG	CGACTTCACCTGCGTACACATC	ACTTCCAAGGGCCGTAAAGTGC	FBgn0259178	CG42283	5PtaseI	5PtaseI
SGI00192	2	175	CCGGTACCATTATAGCGTCG	GTGATCTTGGCCGCATACAT	TGTGAGGACAAGAACCGCTACG	TTTGTCATCGTCGCCTTGCC	FBgn0259227	CG42327	-	CG42327
