# Supplementary material for: Design and evaluation of genome-wide libraries for RNA interference screens
Source: Genome Biol. 2010 Jun 15;11(6):R61. doi: 10.1186/gb-2010-11-6-r61 (PMC2911109; doi:10.1186/gb-2010-11-6-r61)
Supplement: Additional file 9 — RPKM (reads per kilobase gene per million reads) values for 49 Drosophila phosphatases from RNA-sequencing of D.Mel-2 cells and knock-downs measured after RNAi with two independent designs by quantitative RT-PCR (Figure 5; Additional file 10). [file gb-2010-11-6-r61-S9.PDF]

Additional file 9 **Expression values (RNA-Seq RPKMs) and knock-down values for *Drosophila* phosphatases**

FBgnID	AnnotationSymbol	Symbol	RPKM_D.Mel-2	KD_Design1	StdErr-Design1	KD_Design2	StdErr-Design2
FBgn0000382	CG3954	csw	30.13	77.60%	1.50%	73.40%	9.00%
FBgn0000464	CG10443	Lar	5.37	45.20%	6.00%	42.10%	21.60%
FBgn0003134	CG6593	Pp1alpha-96A	48.51	91.10%	1.40%	96.60%	1.20%
FBgn0003138	CG9181	Ptp61F	29.50	74.10%	4.80%	72.10%	5.50%
FBgn0003139	CG12217	PpV	27.33	96.20%	0.30%	90.40%	5.50%
FBgn0003525	CG1395	stg	96.04	-25.40%	7.20%	-10.00%	18.20%
FBgn0004177	CG7109	mts	172.89	92.30%	0.40%	95.90%	0.20%
FBgn0004368	CG6899	Ptp4E	13.12	69.80%	3.20%	70.30%	9.60%
FBgn0004369	CG11516	Ptp99A	12.09	61.90%	7.90%	78.40%	2.10%
FBgn0004370	CG1817	Ptp10D	6.86	61.40%	12.10%	70.70%	20.40%
FBgn0005777	CG8402	PpD3	71.82	90.70%	1.60%	58.30%	29.70%
FBgn0014007	CG10975	Ptp69D	33.39	78.10%	1.50%	89.20%	0.80%
FBgn0016078	CG8804	wun	8.75	75.60%	7.80%	79.60%	5.10%
FBgn0016641	CG9856	PTP-ER	8.65	65.10%	14.10%	77.30%	3.70%
FBgn0022768	CG2984	Pp2C1	10.72	78.60%	2.80%	64.30%	4.70%
FBgn0023508	CG3573	CG3573	11.03	73.60%	8.60%	70.10%	10.70%
FBgn0024734	CG4993	PRL-1	152.25	89.80%	1.80%	94.40%	1.20%
FBgn0025742	CG9115	mtm	14.26	91.90%	1.80%	80.80%	3.60%
FBgn0026379	CG5671	Pten	21.59	79.60%	8.50%	91.50%	2.50%
FBgn0027515	CG7115	CG7115	10.94	60.90%	13.00%	79.10%	4.90%
FBgn0028341	CG32697	l(1)G0232	25.52	79.30%	1.60%	82.70%	2.20%
FBgn0028497	CG3530	CG3530	18.50	89.30%	1.50%	94.30%	0.60%
FBgn0029157	CG6238	ssh	9.04	74.20%	8.00%	68.50%	8.90%
FBgn0029958	CG12151	Pdp	6.52	81.50%	2.00%	74.70%	2.70%
FBgn0030556	CG1810	mRNA-capping-enzyme	23.27	83.6%	2.3%	70.3%	ND
FBgn0030735	CG3632	CG3632	16.11	72.40%	0.60%	73.30%	11.80%
FBgn0030758	CG9819	CanA-14F	13.11	73.90%	8.20%	48.80%	13.80%
FBgn0031044	CG14211	MKP-4	7.38	83.10%	2.60%	81.60%	2.30%
FBgn0031194	CG17598	CG17598	13.96	79.50%	0.60%	80.60%	5.60%
FBgn0031799	CG9493	Pez	8.31	-0.20%	6.60%	21.70%	15.00%
FBgn0031952	CG7134	cdc14	9.24	84.70%	2.20%	84.50%	7.40%
FBgn0032702	CG10376	CG10376	86.83	90.30%	0.40%	86.70%	1.00%
FBgn0033021	CG10417	CG10417	88.69	89.60%	0.20%	93.70%	0.90%
FBgn0034179	CG6805	CG6805	58.34	87.60%	3.70%	89.80%	1.40%
FBgn0034691	CG6562	synj	24.68	93.50%	1.90%	92.30%	0.90%
FBgn0035133	CG1228	Ptpmeg	12.53	78.20%	2.10%	76.80%	3.60%
FBgn0035425	CG17746	CG17746	78.46	93.30%	0.70%	73.50%	22.10%
FBgn0036448	CG9311	mop	36.70	84.90%	2.40%	77.90%	3.30%
FBgn0036551	CG17029	CG17029	6.64	89.10%	0.00%	97.70%	0.70%
FBgn0036844	CG14080	Mkp3	58.55	38.90%	30.90%	83.40%	7.60%
FBgn0037063	CG9391	CG9391	23.91	94.40%	0.30%	96.20%	0.50%
FBgn0037341	CG12746	CG12746	12.39	90.80%	0.10%	86.00%	1.70%
FBgn0039111	CG10371	Plip	16.74	80.30%	2.30%	91.20%	0.00%
FBgn0043903	CG14226	dome	13.90	82.10%	3.80%	93.40%	2.30%
FBgn0062449	CG13197	CG13197	21.32	86.10%	2.30%	87.50%	2.10%
FBgn0086361	CG1906	alph	88.24	88.80%	0.40%	93.20%	1.10%
FBgn0243512	CG7850	puc	64.90	11.30%	15.10%	22.00%	27.70%
FBgn0259178	CG42283	5PtaseI	17.41	66.30%	0.30%	91.50%	3.70%
FBgn0259227	CG42327	CG42327	6.28	75.10%	10.90%	69.10%	8.80%
