# Supplementary material for: Design and evaluation of genome-wide libraries for RNA interference screens
Source: Genome Biol. 2010 Jun 15;11(6):R61. doi: 10.1186/gb-2010-11-6-r61 (PMC2911109; doi:10.1186/gb-2010-11-6-r61)
Supplement: Additional file 11 — Descriptions and default values of design parameters used for NEXT-RNAi version 1.31. [file gb-2010-11-6-r61-S11.PDF]

Additional file 11 **NEXT-RNAi (v1.31) default parameter settings**

Parameter	Description*	Default
**Program start parameters**		
-i	Inputfile containing target sequences (in FASTA format).	-
-s	<int> number of features (FASTA sequences) from input file that are processed at once (optional).	4000
-r	Reagent type (d = long dsRNA, s = short interfering RNA) for designs or evaluations.	-
-d	Location of Bowtie database/index file (pre-build with bowtie-build), multiple inputs are allowed (separated by '+')(optional, if set to 'nodb' NEXT-RNAi will run without 'off-target' evaluation).	-
-e	NO: de novo design of RNAi reagents<br>OLIGO: evaluation of primers for long dsRNAs (-r d) or siRNAs (-r s)<br>DSRNA: evaluation of long dsRNAs (-r d)<br>DSRNA+OLIGO: evaluation of long dsRNAs and underlying primers (-r d)	-
-o	File containing further settings for RNAi reagent design/evaluation in a TAG=VALUE format (optional).	-
-n	Name tag for files generated by NEXT-RNAi (optional).	Probe
-h	Show help (optional).	-
-p	Start interactive setting of NEXT-RNAi options (optional).	-
**Design settings**		
SIRNALENGTH	Set length [nt] of siRNAs used for off-target evaluation.	19
DESIGNWINDOW	Set minimal and maximal length [nt] of desired RNAi reagents separated by comma.	80,500
DESIGNNUM	Set number of RNAi reagents to be designed for each identified specific region in the queried target sequences.	50
OUTPUTNUM	Set number of RNAi reagents to be returned for each queried target sequence.	1
PRIMER3OPT	Set location of file with options for PRIMER3 program in 'TAG=VALUE' format default settings are used otherwise.	disabled
PRIMERTAG	Set sequence to be added 5' to both, forward and reverse primer sequences (for the design of long dsRNAs), e.g. a T7- or SP6-tag for in vitro transcription.	disabled
EFFICIENCY	Set efficiency calculation method and efficiency cutoff score separated by comma. Available calculation methods are 'RATIONAL' for calculations according to Reynolds et al. (requires VIENNA software), or 'SIR' according to Shah et al. The efficiency cutoff defines the minimal required efficiency for a siRNA to be selected (only for de novo designs, -e NO).	disabled
TARGETSEQ	If set to FULL NEXT-RNAi is forced to use the complete input target sequences as design template, if set to CALC only calculated specific regions are considered.	CALC for de novo designs, FULL for evaluations
FEATURE	Set location of file containing feature location information. A tab-delimited file with headers 'FeatureName', 'FeatureLoc' (location in GENOMEBOWTIE / GENOMEFASTA database), 'FeatureStart' (start of feature in GENOMEBOWTIE / GENOMEFASTA) and 'FeatureEnd' (end of feature in GENOMEBOWTIE / GENOMEFASTA database). Requires mapping of reagents to GENOMEBOWTIE or GENOMEFASTA databases.	disabled
LOWCOMPEVAL	Set location of mdust program for the evaluation of low-complexity regions in the input sequences.	disabled
CANEVAL	Option for calculation of CA[ACGT] tandem trinucleotide repeats in target or reagent sequences. This option is enabled by setting the minimal number of CAN repeats (e.g. 6) to be detected.	disabled
SEEDMATCH	Calculation of seed matches from siRNA sense strand (starting at position 2) to a defined FASTA file OR a Bowtie database/index file (if a FASTA file was provided, NEXT-RNAi expects the bowtie-build script for building the bowtie index in the BOWTIE folder). This option requires setting the length of the seed region (between 6 and 8), the maximal seed complement frequency allowed (for filtering of target sequences) and the location of the FASTA file or Bowtie database/index (pre-build with bowtie-build) separated by comma.	disabled
MIRSEED	Calculation of (e.g. miRNA-) seeds within a long dsRNA or siRNA from a given FASTA file containing miRNA sequences. Requires length of seed region (between 6 and 8, starting from position 2 in miRNA sense sequences) and location of FASTA file (separated by comma), siRNAs containing seeds will be excluded from designs.	disabled
POOL	Results for siRNA evaluations can be summarized for pools of sequences. This option requires setting of the location of a tab-delimited file containing the headers 'siRNAID' and 'POOLID' to define connections between query siRNA identifiers and corresponding siRNA-pool identifiers. This options is only available for the evaluation of siRNAs.	disabled
INDEPENDENT	Set location of FASTA file or Bowtie database/index containing sequences that should be avoided for independent reagent designs (file is appended to the off-target database). In case a FASTA file was provided the 'bowtie-build' script is required in the location defined for BOWTIE (to build the Bowtie index).	disabled
INTRON	Percentage nt of a long dsRNA allowed to target intronic regions.	25
RANKD	Long dsRNA designs are by default ranked for percent specificity in first place and number of contained siRNAs predicted to be efficient in second place (RANKD = EFF). NEXT-RNAi can be forced to rank designs for the absolute number of specific siRNAs contained in the long dsRNAs in second place (RANKD = SPEC), which maximized the length of long dsRNA designs.	EFF
REDESIGN	Define whether NEXT-RNAi is allowed to enter a (re-)design method (REDESIGN = ON) to enable the design of RNAi reagents for input sequences that do not meet the user-defined quality measures (specificity (SIRNALENGTH), EFFICIENCY, LOWCOMPEVAL, CANEVAL, SEEDMATCH and MIRSEED).	OFF
OTEEVAL	For evaluation of designed RNAi reagents for 'off-target' effects in additional databases. This options requires the location of a Bowtie database/index; the siRNA length [nt] for mappings; whether off-target effects should be evaluated by positional information ('pos', database has to be the same as in GENOMEBOWTIE / GENOMEFASTA) or by target information ('target' uses targetgroups defined in TARGETGROUPS). Database, siRNA length and evaluation option are separated by comma. Multiple evaluations can be queried.	disabled

**Program locations**		
PRIMER3	Set location of primer3_core script required for primer designs during the design and evaluation of long dsRNAs. Primer3 settings can be influenced in an additional options file (see PRIMER3OPT below).	/usr/bin/
BOWTIE	Set location of bowtie script required by NEXT-RNAi. Bowtie is used for mappings to determine the specificity of an RNAi reagent (against the database defined with -d) and for mappings to determine the location of an RNAi reagent in the genome. The mapping of RNAi reagents is a prerequisite for generation of GFF and AFF output files and for the calculation of FEATURE contents and requires the definition of a mapping database (GENOMEBOWTIE).	/usr/bin/
LOWCOMPEVAL	Set location of mdust program for the evaluation of low-complexity regions in the input sequences.	disabled
BLAT	Set location of blat program for mapping RNAi reagents to the genome. By default BOWTIE is used for mappings. However, if reagents were designed on CDS (SOURCE=CDS) Blat is required to allow for gapped alignments to the genome. BLAT mapping can be influenced by a set of further options (see GENOMEFASTA, BLATALIGN, BLATSPLIT, BLATPROGRAM, BLATHOST, BLATPORT below). The mapping of RNAi reagents is a prerequisite for generation of GFF and AFF output files and for the calculation of FEATURE contents.	disabled
HOMOLOGY	Set location of blastall program, the FASTA database used to determine homology (needs prior formatting to a FASTA database with formatdb command included in Blast package) and the e-value cutoff for homology (e.g. 1e-10). These three input parameters are separated by comma.	disabled
VIENNA	Set location of RNAfold.pl script that belongs to the Vienna RNA package. This program is required only for efficiency predictions using the RATIONAL method (see EFFICIENCY below).	/usr/bin/
**Mapping reagents to the 'off-target' (-d) database**		
TARGETGROUPS	Location of file defining which sequences in the database file (-d option) belong to one group (e.g. splice variants of a gene). A tab-delimited file containing the headers 'Target' (e.g. transcripts) and 'TargetGroup' (e.g. the gene the transcript belongs to) is required. NEXT-RNAi will then consider e.g. siRNAs that target multiple transcripts of the same gene as specific for this gene. Multiple files containing targetgroups can be defined in the options file.	disabled
EXCLUDED	Location of file containing identifiers from the off-target database (-d option) that should be excluded as target sites, but not considered as real off-targets in case they were hit (e.g. UTR regions). A text file with the header 'Exclude' listing identifiers to be excluded is required. Multiple 'EXCLUDED' files can be queried.	disabled
INTENDED	Location of file containing sequence identifiers from the input file connected to their intended target (same as 'TargetGroup' identifier in TARGETGROUPS file) that forces NEXT-RNAi always to output this gene as the primary, intended target of the reagent. A tab-delimited file with the headers 'Query' and 'Intended' listing the identifiers is required. Multiple 'INTENDED' files can be queried.	disabled
**Mapping reagents to the genome using Bowtie**		
GENOMEBOWTIE	Set location of mapping database/index for Bowtie. Bowtie needs mapping databases (indices) that were build with the bowtie-build script from FASTA files. The mapping of RNAi reagents is a prerequisite for generation of GFF and AFF output files and for the calculation of FEATURE contents.	disabled
**Mapping reagent to the genome using blat or gfClient**		
SOURCE	Set type of source where target sequences were retrieved from ('GENOMIC' for genomic (unspliced) sources, 'CDS' for spliced sources). It affects the type of mapping: for 'CDS' sources BLAT is required, for 'GENOMIC' sources BOWTIE is used.	GENOMIC
BLATPROGRAM	Set either to 'blat' for local Blat alignments or to 'gfClient' for alignments using a running Blat server. The 'blat' option requires setting of a FASTA database with the GENOMEFASTA option, the 'gfClient' option requires BLATHOST and BLATPORT settings to connect to the Blat server.	blat
GENOMEFASTA	Set location of FASTA mapping database for Blat. The mapping of RNAi reagents is a prerequisite for generation of GFF and AFF output files and for the calculation of FEATURE contents.	disabled
BLATHOST	Name of server that runs the Blat server (gfServer), required to run Blat mappings using the BLATPROGRAM gfClient.	disabled
BLATPORT	Port to connect to a particular instance (database) of the Blat server defined in BLATHOST. Required to run Blat mappings using the BLATPROGRAM gfClient.	disabled
BLATSPLIT	Split parameter for a large FASTA database defined in GENOMEFASTA (using blat as BLATPROGRAM). The FASTA database will be splitted in parts only containing the defined number of sequences.	0 (no splitting)
BLATALIGN	If set to 'PERFECT', NEXT-RNAi only allows perfect matches during mapping of sequences to the genome with blat or gfClient. If set to 'PARTIAL', also partial mappings are evaluated.	PERFECT
TXNFASTA	Set location of off-target database (as used for -d option) in FASTA format. This option is required for gapped alignments using Blat (e.g. to map siRNAs spanning exon-exon boundaries). NEXT-RNAi can use the mapping information from the off-target database to extend the reagent's sequence and re-map it to the genome.	disabled
**Output settings**		
OUTPUT	Set output folder for files created by NEXT-RNAi.	Input file location
GFF	Enables output of general feature format (GFF) file by choosing either 'GFF2' or 'GFF3' format and requires prior mapping of reagents (see BOWTIE and BLAT options).	disabled
GBROWSEBASE	Set URL to a generic genome browser (GBrowse) instance for visualization of designed reagents in their genomic context. The URL needs to be a link to the 'gbrowse_img' script of the GBrowse instance, e.g. for accessing our Drosophila melanogaster genome browser use http://www.dkfz.de/signaling/cgi-bin/gbrowse_img/flybase/. The visualization requires prior mapping of reagents (see BOWTIE and BLAT options) and further tracks can be added by setting of the GBROWSETRACK option.	disabled

GBROWSETRACK	Set generic genome browser (GBrowse) tracks to be visualized with the designed RNAi reagents. Multiple tracks can be enabled by '+' concatenation (e.g. 'GENE+TXN' for showing genes and transcripts in our Drosophila melanogaster GBrowse, see GBROWSEBASE option). The visualization requires prior mapping of reagents (see BOWTIE and BLAT options) and setting of the GBROWSEBASE URL.	disabled
AFF	Set to 'YES' for generation of an annotations file that allows for the direct upload of design results to GBrowse. This requires prior mapping of reagents (see BOWTIE and BLAT options).	disabled
**Primer3 default settings (can be adjusted using the PRIMER3OPT parameter)**		
PRIMER_OPT_SIZE	Desired (optimal) primer length [nt].	20
PRIMER_MIN_SIZE	Minimal allowed primer length [nt].	18
PRIMER_MAX_SIZE	Maximal allowed primer length [nt].	27
PRIMER_PRODUCT_SIZE_RANGE	Desired product length [nt], defined by DESIGNWINDOW parameter.	80,500
PRIMER_NUM_RETURN	Number of primer designs per optimal target region, defined by DESIGNNUM parameter.	1
PRIMER_PAIR_PENALTY	Maximal allowed primer pair penalty.	-

*Detailed descriptions including examples are available in the NEXT-RNAi Wiki at http:/www.nextrnai.org/
